# Epithelial cells in chronic obstructive pulmonary disease exacerbations: Targets for lung repair

**DOI:** 10.1016/j.pharmr.2026.100131

**Published:** 2026-03-19

**Authors:** Wei Ji, Hayriye Akel Bilgic, Maarten van den Berge, Huib A.M. Kerstjens, Gert Folkerts, Reinoud Gosens, Saskia Braber

**Affiliations:** 1 Division of Pharmacology, Utrecht Institute for Pharmaceutical Sciences, Faculty of Science, Utrecht University, Utrecht, The Netherlands; 2 Groningen Research Institute for Asthma and COPD (GRIAC), University Medical Center Groningen, University of Groningen, Groningen, The Netherlands; 3 Department of Pulmonary Diseases, University of Groningen, University Medical Center, Groningen, The Netherlands; 4 Department of Molecular Pharmacology, University of Groningen, Groningen, The Netherlands; 5 Danone Nutricia Research, Utrecht, The Netherlands

## Abstract

Chronic obstructive pulmonary disease (COPD) is a heterogeneous disease characterized by chronic respiratory symptoms and persistent, often progressive, loss of lung function. Patients, particularly those with severe disease, frequently experience exacerbations triggered by viral and bacterial infections. Exacerbations are closely linked to the rapid decline in lung function, accounting for more than 50% of the accelerated loss over the lifetime of a patient with COPD. Airway epithelial cells play a central role in the development of COPD exacerbations. As the first line of defense, these cells form tight intercellular junctions, creating a protective barrier against pathogens and producing mucus and antimicrobial peptides to eliminate bacteria and viruses. Upon pathogen recognition via pattern recognition receptors, epithelial cells initiate inflammatory responses by releasing cytokines and chemokines that recruit immune cells. This inflammation can become dysregulated in COPD, contributing to tissue damage and exacerbation severity. Furthermore, airway epithelial cells are particularly important in promoting lung repair and restoring homeostasis. They modulate inflammation resolution, promote extracellular matrix remodeling, and support lung regeneration. In COPD, chronic inflammation and repeated exacerbations impair epithelial function, disrupt repair mechanisms, and hinder lung regeneration, contributing to irreversible damage. This review highlights the crucial role of airway epithelial cells in COPD exacerbations, focusing on their regulatory functions in maintaining barrier integrity, managing inflammation and promoting epithelial lung repair. Moreover, current and future therapeutic strategies aimed at enhancing epithelial barrier function, controlling airway inflammation, and supporting lung recovery and regeneration are discussed, highlighting key directions for future investigation.

**Significance Statement:**

Chronic obstructive pulmonary disease exacerbations represent a pharmacologically actionable period characterized by epithelial barrier disruption, dysregulated inflammation, and impaired repair. This review positions airway epithelial cells as central orchestrators of these processes, regulating host defense, immune interactions, and regenerative responses. By targeting epithelial dysfunction during exacerbations, this review highlights the potential therapies that go beyond symptom relief and support lung repair and modify disease progression.

## I Introduction

Chronic obstructive pulmonary disease (COPD) is a heterogeneous inflammatory lung disease with persistent symptoms such as shortness of breath, coughing, and excessive sputum production.^1^ COPD is characterized by inflammation, emphysema, airway remodeling, and impaired lung function. These pathological features arise from environmental exposures and genetic or lifestyle factors that cause tissue injury and disrupt normal lung repair and regeneration.^2^^,^^3^ Although multiple exposures including ambient air pollution, biomass fuel smoke, and occupational hazards contribute to disease development, tobacco smoking is widely recognized as the primary exposure risk factor.^4^^,^^5^

COPD is usually a progressive disease, and many patients, especially those with severe disease, experience exacerbations, acute episodes of worsening respiratory symptoms.^1^ It is estimated that about 80% of the exacerbations are caused by viral or bacterial infections or coinfections.^6^ In addition, environmental exposures, such as air pollution, trigger inflammatory responses that may provoke COPD exacerbations.^7^^,^^8^ Progression of COPD is closely linked to the frequency and severity of exacerbations, which have significant clinical and economic implications, including reduced productivity, diminished quality of life, deteriorating lung function, impaired exercise capacity, and increased mortality.^1^^,^^9^ Exacerbations are estimated to account for more than 50% of the decline in lung function over the lifetime of a patient with COPD. Importantly, lung function decline is not linear. Instead, it reflects cumulative losses driven by recurrent exacerbations, which increase in frequency and severity as the disease progresses.^10^

At the mechanistic level, airway epithelial cells play a central role in COPD exacerbations by acting as both a physical barrier and active regulators of immune responses. As part of the physical defense, these cells support mucociliary clearance and produce antimicrobial peptides (AMPs) and proteins.^11^^,^^12^ Additionally, these cells release a range of molecules such as cytokines, chemokines, and growth factors, which recruit and activate cells such as phagocytes, thereby initiating and coordinating immune and inflammatory responses.^13^^,^^14^ The inflammatory response activates epithelial repair mechanisms, including epithelial-to-mesenchymal transition (EMT), altered migration and proliferation of basal or progenitor cells, and dysregulated redifferentiation, which can result in aberrant epithelial phenotypes such as goblet cell hyperplasia and squamous metaplasia, hallmarks of airway remodeling.^15^^,^^16^ In the lung, bacterial and viral infections trigger activation of epithelial progenitor cells, aiding in the recovery from (severe) lung injury.^17^^,^^18^ However, in COPD, although this recovery phase is initiated, it remains incomplete, and persistent lung inflammation develops, resulting in a net decline in lung function.^10^ Although current treatments such as bronchodilators, oral steroids, and antibiotics can mitigate the frequency and severity of exacerbations, these medications mainly focus on symptom relief and exacerbation prevention without directly addressing tissue repair or halting the decline in lung function.^1^ In fact, only 2 new classes have been introduced for COPD treatment over the past 2 decades: phosphodiesterase (PDE) 4 inhibitors^19^ and, more recently, interleukin (IL) 4 receptor *α* blockers.^20^ Therefore, it is urgent to seek treatment strategies that focus on the abnormal interaction between inflammation and lung repair processes, aiming to prevent lung function decline or improve lung function by promoting tissue repair after exacerbations.

In this review, the role of airway epithelial cells in COPD exacerbations is discussed, with a focus on their functions in maintaining barrier integrity and regulating inflammation. We highlight the epithelial cell interaction with immune cells, as well as their potential for lung regeneration and repair. Finally, we focus on the current and future therapies aimed at enhancing barrier function, reducing inflammation, and promoting lung regeneration to prevent lung function decline.

## II Chronic obstructive pulmonary disease exacerbations

Given the diverse causes and varying levels of severity associated with acute COPD exacerbations, there has been debate regarding the accurate definition of exacerbations. Following the updated 2025 GOLD COPD Report, COPD exacerbations are described as “an event characterized by increased dyspnea and/or cough and sputum that worsens in <14 days, which may be accompanied by tachypnea and/or tachycardia and is often associated with increased local and systemic inflammation caused by infection, pollution, or other insults to the airways.”^1^

Acute exacerbations of COPD represent critical clinical events in the natural history and management of the disease, exerting a substantial adverse impact on health status, healthcare utilization, and long-term outcomes.^9^^,^^21^ These episodes are associated with an increased risk of hospitalization and readmission and contribute to an accelerated decline in lung function and overall disease progression.^22^^,^^23^ Pathophysiologically, exacerbations are characterized by amplified airway and systemic inflammation, enhanced mucus hypersecretion, and pronounced gas trapping with dynamic hyperinflation.^1^ Collectively, these pathophysiologic alterations provide the mechanistic basis for worsening dyspnea, the hallmark clinical feature of an exacerbation. Additional manifestations commonly include increased sputum volume and purulence, together with aggravated cough and wheeze.^24^^,^^25^ During exacerbations, patients are also at heightened risk of concurrent acute conditions, notably decompensated heart failure, pneumonia, and pulmonary embolism.^26^, ^27^, ^28^ Such comorbid events may either mimic an exacerbation or exacerbate its clinical expression, thereby complicating diagnosis and management.

Although exacerbation-related symptoms typically persist for 7–10 days, a substantial proportion of patients experience a more prolonged course. Notably, up to 20% of individuals do not recover to their pre-exacerbation clinical baseline within 8 weeks.^29^ Exacerbations are increasingly recognized as critical drivers of disease progression, particularly in the context of incomplete or delayed recovery.^30^ Furthermore, exacerbations demonstrate temporal clustering, whereby the occurrence of one episode confers a significantly elevated risk of subsequent events.^31^

Viral and bacterial infections represent the most common triggers of COPD exacerbations.^11^ Approximately 22%–80% of COPD exacerbations are related to viruses.^32^^,^^33^ *Rhinovirus* is one of the most common viruses associated with COPD exacerbations worldwide.^33^ In a systematic review of 19 studies involving 1728 patients, *Rhinovirus* showed the highest detection rate among the identified viruses at 16.39%. Other viruses, such as respiratory syncytial virus (9.90%) and *influenza* (7.83%), were also associated with exacerbations but were less prevalent.^34^ Furthermore, other viruses including human metapneumovirus*,* coronavirus, and adenovirus are also implicated in provoking COPD exacerbations.^32^^,^^35^ However, the predominant viruses differed among studies, in some cohorts influenza was more frequently detected, whereas in others respiratory syncytial virus was more common. These differences may be associated with factors such as study population, sampling time, and detection methods.^36^

Bacterial infections are associated with COPD exacerbations as well, with a prevalence ranging from 26% to 81%.^37^ Multiple studies on COPD exacerbations have found that the lung microbiota mainly consists of nontypeable *Haemophilus influenzae* (*NTHI*), *Streptococcus pneumoniae*, and *Klebsiella pneumoniae*^38^ but many more can be found. *NTHI* is the most frequently found bacterium in the distal airways of patients with COPD during exacerbation.^39^

Importantly, approximately 25% of patients hospitalized for COPD exacerbations have detectable coinfection with both viruses and bacteria, which is associated with more severe lung function impairment, prolonged hospitalization, increased exacerbation severity, and a higher risk of readmission.^40^^,^^41^

Environmental factors may also play a role in provoking COPD exacerbations. Short-term exposure to air pollutants, such as PM_2.5_, contributes to the risk of exacerbations.^7^ The effects of extremely cold and heat,^42^ the occurrence of floods, tornadoes, volcanic eruptions, dust storms, and earthquakes increase the incidence of COPD exacerbations.^7^ Furthermore, exposure to cigarette smoke, a major environmental risk factor, increases the risk of exacerbations by damaging lung tissue, increasing inflammation, and enhancing susceptibility to respiratory infections.^43^^,^^44^

## III The role of airway epithelial cells in chronic obstructive pulmonary disease exacerbations

Airway epithelial cells are key drivers of COPD exacerbations, acting not only as structural barriers but also as active players in immune regulation and tissue repair.^11^^,^^45^ However, during the exacerbation of COPD, the dysregulated immune response caused by airway epithelial cell injury leads to lung tissue destruction and abnormal remodeling of the airways.^11^ Airway epithelial cells can influence the pathophysiology of COPD exacerbations through several mechanisms, including (1) maintenance of the physical barrier, (2) regulation of inflammatory responses, (3) interactions with immune cells, and (4) lung tissue repair.

### A Airway epithelial cells as regulators of barrier function

Airway epithelial cells are essential regulators of barrier function within the respiratory system. This function is coordinated by a combination of key components, including mucociliary clearance, the apical junctional complex (AJC), and the secretion of AMPs. Together, these elements form a robust defense mechanism, actively working to eliminate inhaled pathogens, such as bacteria and viruses, and maintain tissue homeostasis while minimizing inflammatory responses. However, individuals with COPD experience impairments in 1 or more of these critical barrier functions. Such impairments increase susceptibility to viral or bacterial infections and foster excessive and prolonged immune responses to pathogens.^46^ Consequently, the inflammatory response intensifies, leading to acute COPD exacerbations. Understanding the intricate role of airway epithelial cells in regulating barrier function is essential for developing targeted therapeutic interventions to mitigate the impact of COPD exacerbations.

#### 1 Mucociliary clearance

Mucociliary clearance serves as a primary defense mechanism in the airways, removing pathogens and debris through mucus secretion and ciliary motion.^47^ Goblet cells and submucosal glands secrete mucus, which contains mucin glycoproteins (eg, MUC5AC and MUC5B)^48^ that trap pathogens, while cilia propel mucus toward the pharynx for expulsion, thereby maintaining respiratory health and function.^49^ Beyond mucins and cilia, airway surfactant contributes to mucociliary transport. A thin film of surface-active phospholipids, primarily dipalmitoyl-phosphatidylcholine and anionic phosphatidylglycerol, together with the hydrophobic proteins surfactant protein (SP)-B/SP-C, lowers interfacial tension at the air–mucus interface and reduces mucus adhesion, thereby facilitating ciliary propulsion.^50^ The collectins SP-A and SP-D further opsonize microbes and modulate epithelial/macrophage responses, linking mucociliary transport to innate immunity. However, this critical defense mechanism is often impaired in patients with COPD,^45^ with impairments worsening during exacerbations, primarily due to excessive mucus production and defective clearance.^51^ Cigarette smoke and oxidative stress can perturb surfactant composition, and circulating SP-D is elevated in COPD and tends to rise further during exacerbations, consistent with barrier injury.^52^ Studies have shown that during COPD exacerbations, MUC5AC and MUC5B protein levels are elevated compared with those observed in stable COPD.^53^^,^^54^ As a result, mucus accumulation obstructs the airways and reduces the efficiency of pathogen clearance, creating favorable conditions for microbial colonization and infection, further exacerbating inflammation and tissue damage.^55^

This dysfunction in mucociliary clearance is further aggravated by changes in mucus composition and ciliary function, particularly during acute COPD exacerbations. Patients with acute exacerbations of COPD often exhibit excessive mucus secretion and altered mucus composition, resulting in thicker and more viscous mucus that is difficult to clear.^48^^,^^55^ Key ciliary characteristics such as structure, number, ciliary beat frequency, wave pattern, and orientation are essential for normal function. However, these properties are often disrupted in acute COPD exacerbations, further contributing to mucus accumulation.^11^ Moreover, external factors such as smoking exacerbate mucociliary dysfunction by impairing ciliary function and reducing the number of ciliated cells, thereby diminishing mucus clearance efficiency.^11^^,^^56^

In addition, the Notch signaling pathway plays a key regulatory role in maintaining the balance between airway mucus secretion and ciliary clearance. This pathway activates Notch receptors through *γ*-secretase–mediated cleavage, releasing their intracellular domains to translocate into the nucleus and regulate epithelial cell fate determination.^57^, ^58^, ^59^ Activation of Notch signaling tends to drive differentiation into secretory cells, whereas the absence of Notch activation favors the formation of multiciliated cells.^57^^,^^58^ In COPD, this pathway is often aberrantly activated, with upregulation of Notch3 closely associated with goblet cell hyperplasia and airway remodeling,^60^^,^^61^ further disrupting the dynamic balance between mucus production and clearance. In airway epithelial cells derived from patients with COPD, Notch3 has been shown to drive excessive mucus production during *Rhinovirus* infection. This process relies on a *γ*-secretase–dependent Notch3 signaling cascade and may contribute to mucus retention and impaired mucociliary clearance during acute exacerbations.^62^

Collectively, impaired mucociliary clearance reflects disrupted epithelial differentiation and ciliary function, weakening epithelial defense and predisposing to infection-driven inflammatory amplification during COPD exacerbations.

#### 2 Apical junctional complex

AJC is a dynamic structure essential for maintaining epithelial integrity, ensuring cell polarity, barrier function, and structural stability. It consists of tight junctions (eg, occludins and zona occludens-1 [ZO-1]), adherens junctions (eg, cadherins), gap junctions (eg, connexins), and desmosomes (eg, desmogleins), which collectively regulate permeability and preserve airway epithelial integrity. In healthy individuals, components of the AJC work together to create a strong and selective barrier, protecting the airways from inhaled pathogens and environmental pollutants.^63^ In COPD, the AJC is disrupted by chronic inflammation, oxidative stress, and cigarette smoke exposure, leading to impaired epithelial function.^63^^,^^64^ This disruption compromises key junctional proteins responsible for maintaining epithelial integrity, including occludins, E-cadherin, and claudins (CLDNs), whose altered expression is implicated in COPD pathogenesis.^65^ Patients with COPD exhibit reduced occludin, ZO-1 and E-cadherin expression in airway epithelial cells, with the most severe decline in those with frequent exacerbations.^66^, ^67^, ^68^, ^69^ Notably, reduced E-cadherin expression has also been observed in lung tissue from patients with COPD compared with control subjects with matched smoking histories.^70^

In addition to occludin and E-cadherin, members of the CLDN family, which are crucial components of tight junctions, have been implicated in COPD pathogenesis. CLDN5, a tight junction protein expressed in both endothelial and epithelial cells, is significantly lower in the plasma of patients with COPD compared with healthy individuals, with a slight increase during exacerbations.^71^ The increase in plasma CLDN5 levels during exacerbations indicates its role in inflammatory responses, positioning it as a potential biomarker for COPD severity.^71^ Similarly, CLDN4 has also been linked to the progression of COPD. A study comparing lung function and plasma CLDN4 levels in patients with COPD and healthy controls revealed that stable patients with COPD had significantly lower CLDN4 levels, whereas levels increased markedly during exacerbations. Furthermore, during exacerbations, higher plasma CLDN4 levels were found to be associated with impaired lung function as reflected by lower forced vital capacity and forced expiratory volume in 1 second (FEV1). The elevation of CLDN4 and CLDN5 in plasma during exacerbations compared with stable COPD likely reflects epithelial barrier disruption, leading to the leakage of tight junction proteins into the circulation. This increase may also represent part of a compensatory inflammatory response aimed at limiting further tissue damage and preserving remaining barrier function.^72^ Future research should investigate the role and mechanisms of CLDN4 and CLDN5 in lung tissues, sputum, and bronchoalveolar lavage fluid (BALF) samples to better understand their biological significance in COPD exacerbations.

Disruption of epithelial junctions facilitates pathogen invasion and amplifies inflammation, highlighting the airway epithelium as an active contributor to disease progression during COPD exacerbations and a critical determinant of impaired recovery.

#### 3 Antimicrobial peptides

AMPs are small, bioactive molecules predominantly produced by airway epithelial cells as part of the respiratory system’s innate immune defense.^73^ These peptides exert broad-spectrum antimicrobial activity by disrupting microbial membranes, inhibiting protein synthesis, and modulating immune responses. Among the key AMPs, defensins and cathelicidins play a central role, whereas additional antimicrobial proteins, such as lactoferrin and secretory leukocyte protease inhibitor (SLPI), along with antimicrobial SPs SP-A and SP-D, further enhance host defense mechanisms.^74^

During COPD exacerbations, the expression and secretion of these AMPs are often dysregulated, increasing the risk of airway infections and exacerbating inflammatory responses. Patients with COPD exhibit altered AMP levels compared with healthy individuals.^75^ In particular, cathelicidin levels increase, whereas SLPI levels decrease in sputum during infectious exacerbations and bacterial colonization compared with stable COPD, suggesting that this imbalance may contribute to airway vulnerability and disease progression.^76^, ^77^, ^78^ Additionally, human *β*-defensin-2 production was significantly reduced in airway epithelial cells obtained from both healthy smokers and patients with COPD during in vitro viral and bacterial coinfections, compared with cells from healthy nonsmokers.^79^ This impaired response may compromise epithelial defense and contribute to more severe exacerbations. Furthermore, plasma SP-A and serum SP-D levels are increased during COPD exacerbations compared with the stable disease phase, reflecting continued dysregulation of surfactant-associated immune mediators.^80^^,^^81^ Interestingly, several studies have observed an inverse relationship between SP-D levels in BALF and blood in COPD, although the underlying mechanism remains uncertain.^82^^,^^83^ Sin et al^84^ observed that decreased SP-D concentrations in BALF may reflect its translocation from the alveolar space into the circulation. This phenomenon may result from inflammation-induced disruption of SP-D multimeric aggregates into lower molecular weight forms that more readily diffuse into the circulation, as well as from increased alveolar-capillary permeability.^52^ However, direct evidence supporting this mechanism remains limited.

AMP dysregulation has been the focus of long-term clinical studies investigating its effects on airway infections and COPD exacerbations. A 3-year study from the Bergen COPD Cohort, involving 433 patients with COPD and 325 controls, investigated the role of AMPs in stable COPD and acute exacerbations. Researchers analyzed sputum and plasma levels of cathelicidin (human cationic antimicrobial protein of 18 kilodaltons [hCAP18]/LL-37) and SLPI. Sputum AMP levels were higher in stable patients with COPD than in controls and further altered during exacerbations, with hCAP18/LL-37 levels increasing and SLPI levels decreasing. Similar patterns were observed in plasma hCAP18/LL-37 levels. Notably, higher sputum hCAP18/LL-37 levels in stable COPD were linked to an increased risk of exacerbations, airway colonization by *NTHI*, and elevated inflammatory markers, suggesting a role for AMPs in COPD progression and airway defense.^78^ In a 6-year study involving 153 sputum samples from 11 patients with COPD, samples were categorized based on the presence of pathogens, including nonpathogenic, colonized, and exacerbated states due to *NTHI* and *Moraxella catarrhalis.* Levels of lysozyme, lactoferrin, LL-37, and SLPI were measured, revealing significant reductions in lysozyme and SLPI during colonization and exacerbation when compared with stable COPD, particularly in cases involving *NTHI* and *M catarrhalis*. In contrast, LL-37 levels increased during exacerbations compared with stable COPD.^85^ Together, these studies indicate that exacerbation-associated shifts in epithelial AMP profiles are linked to impaired antimicrobial defense and sustained inflammatory burden. Thus, epithelial AMP dysregulation contributes to impaired host defense and infection-driven inflammatory amplification during COPD exacerbations.

**Fig. 1:**
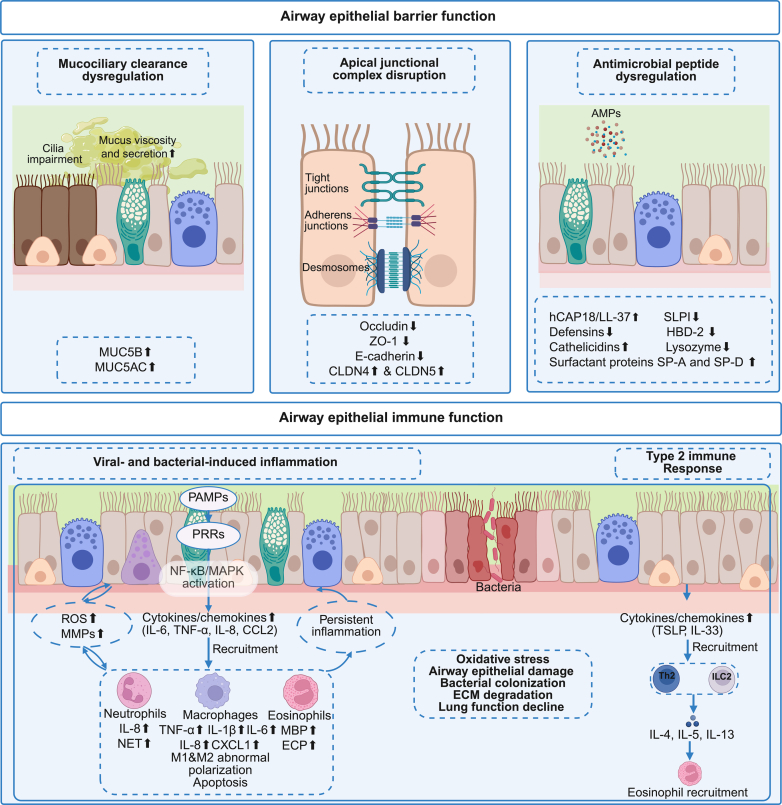
The role of airway epithelial cells in COPD exacerbations. Airway epithelial cells regulate barrier and immune function during and after COPD exacerbations through mucociliary clearance, the AJC, and the secretion of AMPs. Dysregulation in these mechanisms, such as impaired ciliary function, excessive mucus production (MUC5AC and MUC5B), decreased expression of tight junctions (occludin and ZO-1), adherens junctions (E-cadherin) in airway epithelial cells and increased levels of CLDNs (CLDN4 and CLDN5) in serum, or dysregulated release of AMPs (defensins, cathelicidins, lysozyme, SLPI, surfactant-associated proteins SP-A and SP-D) in sputum and plasma/serum, compromise epithelial integrity, increasing susceptibility to infections and inflammatory responses. Furthermore, airway epithelial cells release cytokines (IL-6, TNF-*α*, and IL-8) and chemokines (C-C motif chemokine ligand 2 [CCL2]), which recruit immune cells (neutrophils, macrophages, and eosinophils). These recruited cells, in turn, produce additional cytokines and chemokines, further amplifying the inflammatory response. The generation of ROS and upregulation of MMPs contribute to ECM degradation, perpetuating tissue damage and sustaining a chronic inflammatory cycle. Additionally, the type 2 immune response, characterized by the release of cytokines such as IL-4, IL-5, and IL-13, promotes eosinophil recruitment and activation. These combined effects lead to increased oxidative stress and bacterial colonization, resulting in airway epithelial damage, ECM degradation, and a decline in lung function.

During acute COPD exacerbations, airway epithelial barrier function is impaired through combined defects in mucociliary clearance, epithelial junction integrity, and AMP secretion. Reduced pathogen clearance, increased epithelial permeability, and weakened innate defense promote persistent inflammation and increase susceptibility to infection. These epithelial abnormalities also interfere with normal repair processes, limiting recovery after exacerbations and contributing to disease progression. Figure 1 provides a conceptual summary of airway epithelial barrier dysfunction and epithelial immune responses during COPD exacerbations.

### B Airway epithelial cells as regulators of immune function

#### 1 Inflammation

Airway epithelial cells actively regulate inflammatory responses by producing and releasing various mediators, such as cytokines and chemokines, in response to pathogens or inflammatory stimuli.^86^ These molecules attract immune cells to the infection site, orchestrating the immune response to ensure efficient pathogen clearance.^87^ Both viral and bacterial infections significantly contribute to COPD exacerbations, triggering inflammation through different but overlapping mechanisms.^11^ Although some inflammatory pathways are shared between viral and bacterial infections, each pathogen type triggers distinct immune responses that contribute to the severity and progression of exacerbations. Furthermore, a subset of patients with COPD exhibits type 2 inflammatory responses, characterized by cytokines such as IL-4 and IL-13, particularly in response to environmental irritants or viral infections during exacerbations, adding complexity to the inflammatory profile.^88^, ^89^, ^90^, ^91^ Notably, in patients with prior exacerbation history, this type 2 phenotype is strongly associated with an increased risk of future acute exacerbations.^20^^,^^92^

##### a Shared mechanisms of viral- and bacterial-induced inflammation

Viral and bacterial infections significantly drive COPD exacerbations by triggering inflammatory responses mediated by the airway epithelium. Both types of infections activate pattern recognition receptors on epithelial cells, such as Toll-like receptors (TLRs), including TLR3, TLR4 and nucleotide-binding oligomerization domain-like receptors (NOD-like receptors or NLRs), such as NOD-like receptor family, pyrin domain containing 3 (NLRP3).^93^ These receptors recognize pathogen-associated molecular patterns, leading to the activation of downstream signaling cascades, including the nuclear factor-*κ*B and mitogen-activated protein kinase pathways, which amplify inflammatory responses.^94^^,^^95^ As a result of this activation, epithelial cells release proinflammatory cytokines such as IL-6 and tumor necrosis factor-*α* (TNF-*α*), which further stimulate the recruitment of immune cells, including neutrophils and macrophages.^96^^,^^97^ Additionally, chemokines such as IL-8 and C-C motif chemokine ligand 2 are secreted, attracting these immune cells to the site of infection and exacerbating local inflammation.^98^^,^^99^ Reactive oxygen species (ROS), reactive nitrogen species and matrix metalloproteinases (MMPs) are also produced by inflammatory cells, further damaging epithelial cells and the extracellular matrix (ECM).^100^, ^101^, ^102^, ^103^, ^104^ These shared pathways highlight the central role of airway epithelial cells as regulators of inflammation during COPD exacerbations and underscore the importance of targeting these mechanisms to alleviate airway inflammation and promote lung repair.

##### b Differences between viral- and bacterial-induced inflammation

Despite their similarities, viral- and bacterial-induced inflammation exhibit distinct characteristics in COPD exacerbations. Viral infections primarily provoke type I interferon (IFN) (IFN-*α*/*β*) and type III IFN (IFN-*λ*) responses in epithelial cells, which are essential for antiviral defense.^105^^,^^106^ Integrated evidence from in vivo*,* ex vivo, and clinical studies demonstrates characteristic antiviral immune defects in COPD. Animal models reveal that cigarette smoke exposure exacerbates airway inflammation during *NTHI* infection while concurrently impairing IFN-*γ* production in antigen-specific T cells.^107^ These findings correlate with clinical observations showing a 50% reduction in IFN-*α*/*λ* secretion from BALF macrophages upon *Rhinovirus* challenge in patients with COPD versus healthy controls,^108^ as well as persistently decreased expression of type I/III interferons and interferon-stimulated genes in sputum cells from frequent exacerbators during both stable and acute exacerbation phases.^109^ In the lung organ culture model, cigarette smoke extract (CSE) exposure similarly suppressed both IFN-inducible protein-10 production and its mRNA expression.^110^ IFN-*β* provides durable antiviral protection against *Rhinovirus* in both human alveolar epithelial cells and primary bronchial epithelial cells,^111^ and pretreatment significantly reduced viral replication in these models.^111^^,^^112^ Because the airway epithelium is the primary site of viral infection and replication,^11^ a weakened interferon response may result in uncontrolled viral replication and an excessive inflammatory reaction, contributing to virus-induced COPD exacerbations.^97^ Viral infections also promote the recruitment and activation of various leukocytes, primarily cytotoxic lymphocytes such as CD8^+^ T cells and natural killer cells, which collectively contribute to antiviral immune responses and inflammation in the airways.^113^^,^^114^ Beyond immune dysfunction, most respiratory viruses directly target airway epithelial cells, disrupting barrier integrity, promoting microvascular dilation, edema, and immune cell infiltration.^69^^,^^115^^,^^116^ These effects worsen inflammation and facilitate bacterial colonization and secondary infections,^117^ amplifying the inflammatory burden and accelerating lung function decline.^33^

Compared with viral infections, bacterial infections more consistently and robustly stimulate the release of IL-1*β* and granulocyte-macrophage colony–stimulating factor by airway epithelial cells, thereby promoting neutrophil-dominated inflammation.^118^, ^119^, ^120^, ^121^ This inflammatory response is closely linked to chronic bacterial colonization in COPD. Interestingly, the sputum glucose level, a marker of bacterial activity and inflammation, is significantly increased in patients with COPD compared with healthy individuals.^122^ This glucose-rich environment, combined with excessive mucus production and impaired mucociliary clearance, may foster bacterial proliferation and potentially reshape the lung microbiome.^122^^,^^123^ This can trigger persistent airway inflammation, exacerbate disease progression, and contribute to long-term bacterial colonization. This self-perpetuating cycle of infection and inflammation, often referred to as the “vicious circle” hypothesis, indicates that chronic bacterial colonization not only increases the frequency of exacerbations but also accelerates lung function decline.^11^^,^^124^ As a result, bacterial infections in COPD are not only acute triggers of exacerbations but also long-term contributors to disease progression.

Recent research has provided new insights into the molecular mechanisms behind these differences. Distinct gene expression profiles and biological pathways activated by viral and bacterial exacerbations were identified. In viral exacerbations, proinflammatory responses and mitochondrial damage were observed, whereas bacterial exacerbations triggered pathways related to phagocytosis and TLR signaling, both linked to neutrophil-driven inflammation. Additionally, biomarkers such as interferon-induced transmembrane protein 3 and interferon-stimulated gene 15 were associated with bacterial exacerbations, whereas defensin *α* 3 was identified for viral exacerbations.^125^ Therefore, whether an exacerbation is viral or bacterial is largely shaped by the specific response profile of airway epithelial cells, alongside contributions from immune cell activation.

##### c Type 2 immune responses in chronic obstructive pulmonary disease exacerbations

Inflammation in COPD was initially thought to be primarily driven by macrophages and neutrophils.^126^ However, 20%–40% of patients with COPD exhibit type 2 inflammation,^127^^,^^128^ a characteristic more commonly associated with asthma, involving CD4^+^ T helper type 2 (Th2) cells, type 2 innate lymphoid cells (ILC2s), eosinophils, and other related immune components.^126^^,^^129^ Airway epithelial cells play a key role in initiating type 2 immune responses by releasing alarmins that activate ILC2s and promote eosinophilic inflammation.^130^ Type 2 inflammation is commonly characterized by the increased expression of type 2 cytokines, including IL-4, IL-5, and IL-13, which are predominantly produced by Th2 cells and ILC2s.^131^ This inflammatory profile is closely associated with an increased risk of future exacerbations, particularly in patients with a history of such events.^129^^,^^132^ In patients experiencing acute exacerbations of COPD, there is a pronounced activation and proliferation of Th2 cells in peripheral blood, leading to a significant decrease in the Th1/Th2 ratio and a shift toward a Th2-dominant response.^133^

IL-4 and IL-13 are central to the pathophysiology of type 2 inflammation, signaling through the shared IL-4R*α* receptor found on airway epithelial cells and various immune cells. These cytokines are pivotal in activating and directing eosinophils and other type 2 inflammatory cells to the lungs, primarily through the secretion of eotaxin-3 from epithelial cells.^134^, ^135^, ^136^ Furthermore, IL-4 and IL-13 disrupt the production of IFN-*λ* in response to *Rhinovirus* by inhibiting TLR signaling, which exacerbates viral replication, an essential aspect considering that viral infections frequently trigger COPD exacerbations.^137^ Beyond their roles in inflammation, IL-4 and IL-13 contribute to structural changes in the airways, including airway remodeling, lung tissue damage, and mucus cell hyperplasia, highlighting their broad impact on respiratory health.^138^^,^^139^

Additionally, airway epithelial cells release key mediators such as IL-33 and thymic stromal lymphopoietin (TSLP), which are crucial in exacerbating type 2 immune responses. IL-33, a cytokine released in response to cellular damage, significantly amplifies type 2 inflammation.^140^ A study has shown that IL-33 release from epithelial cells is heightened in mice exposed to cigarette smoke and subsequently infected with influenza, compared with those with viral infections alone, suggesting that IL-33-mediated responses are intensified under these stress conditions.^141^ IL-33 levels are significantly elevated in the plasma and lung tissue of patients with COPD, and higher IL-33 expression is associated not only with greater symptom severity but also with an increased risk of future exacerbations during prospective follow-up.^142^^,^^143^ In addition, an overproduction of TSLP is observed in bronchial epithelial cells after viral infections, underscoring its crucial role in COPD exacerbations.^144^ TSLP acts to further drive the immune response toward a type 2 phenotype, engaging additional immune pathways that contribute to the chronic inflammatory state characteristic of COPD, especially during acute exacerbations.^145^

Airway epithelial cells, therefore, play an important role not only in initiating but also in amplifying type 2 immune responses. Understanding this epithelial-driven inflammation opens up potential therapeutic avenues targeting IL-4, IL-13, TSLP, and IL-33 to reduce inflammation and alleviate exacerbations, ultimately improving outcomes in patient with COPD.

#### 2 Interaction with immune cells

Airway epithelial cells actively regulate immune cell recruitment, activation, and function during COPD exacerbations, thereby shaping the balance between host defense, inflammation, and tissue repair. In response to infection or injury, epithelial cells release chemokines and cytokines that recruit inflammatory cells such as neutrophils, macrophages, and eosinophils to the airways. Although this epithelial-driven immune response is essential for pathogen clearance, its dysregulation during exacerbations promotes epithelial injury, barrier disruption, and impaired recovery.

Neutrophils are among the earliest immune cells recruited in response to lung injury caused by infection, pollutants, and inflammation, and their recruitment during acute exacerbations is primarily driven by chemokines released by airway epithelial cells, such as IL-8.^146^^,^^147^ Excessive neutrophil activation results in the release of neutrophil extracellular traps (NETs).^148^, ^149^, ^150^ Elevated NET levels in both stable and exacerbated COPD promote emphysema progression, airway leukocyte infiltration, and mucus hypersecretion,^148^, ^149^, ^150^ thereby amplifying epithelial injury and barrier dysfunction. Increased NET formation during exacerbations is associated with neutrophil-dominant airway inflammation and greater exacerbation severity in COPD.^151^ Although neutrophils play an essential role in pathogen clearance, uncontrolled neutrophil activity exacerbates epithelial injury and delays repair. Evidence suggests that epithelial damage induced by neutrophil mediators may secondarily initiate repair programs.^152^ However, during COPD exacerbations, this balance frequently shifts toward persistent injury and airway remodeling.^153^^,^^154^

Macrophages interact closely with airway epithelial cells to coordinate immune defense and tissue repair under homeostatic conditions^155^; however, this crosstalk becomes pathologically altered in COPD.^156^, ^157^, ^158^ During acute exacerbations, oxidative stress and sustained inflammatory signaling involving TNF-*α*, IL-1*β*, and IL-6 disrupt epithelial-macrophage communication and granulocyte-macrophage colony–stimulating factor-dependent repair signaling, thereby impairing macrophage-mediated support of epithelial proliferation and differentiation.^118^^,^^159^^,^^160^ At the same time, macrophage-derived C-X-C motif chemokine ligand 1, IL-8, and MMPs promote inflammatory cell recruitment and compromise epithelial barrier integrity, creating a self-reinforcing cycle of inflammatory amplification and defective repair.^69^^,^^161^, ^162^, ^163^ Abnormal polarization and impaired phagocytic capacity further facilitate persistent bacterial colonization, intensifying tissue injury and shifting epithelial-macrophage interactions from a homeostatic regulatory axis toward a driver of sustained inflammation and regenerative failure.^158^^,^^159^^,^^164^, ^165^, ^166^

Eosinophil-epithelial interactions represent an additional layer of immune regulation in COPD exacerbations. Upon recognition of viral or bacterial pathogens through TLRs, airway epithelial cells release chemokines such as regulated on activation normal T cell expressed and secreted, eotaxin, and IL-5, thereby recruiting and activating eosinophils.^167^^,^^168^ Activated eosinophils release cytotoxic granule proteins, including major basic protein and eosinophil cationic protein, as well as ROS, which damage epithelial cells and disrupt barrier integrity and contribute to ECM degradation.^169^, ^170^, ^171^ Injured epithelial cells subsequently release alarmins including IL-33, IL-25, and TSLP, amplifying type 2 inflammatory responses via ILC2 and Th2 pathways.^171^ During acute exacerbations, eosinophil levels in both the sputum and blood are elevated and are closely associated with exacerbation risk and disease progression.^89^^,^^172^, ^173^, ^174^, ^175^, ^176^ Furthermore, eosinophilic COPD represents a distinct inflammatory phenotype characterized by specific inflammatory features and enhanced corticosteroid responsiveness.^177^^,^^178^ These observations suggest that epithelial-eosinophil interactions not only contribute to the initiation and amplification of exacerbations but also influence disease phenotypes and therapeutic responses.^179^, ^180^, ^181^

Overall, dysregulated epithelial control of immune cell responses during COPD exacerbations sustains inflammatory injury, disrupts barrier integrity, and interferes with effective tissue repair. Rather than resolving inflammation, altered epithelial-immune crosstalk reinforces airway remodeling and increases susceptibility to recurrent exacerbations. Figure 1 summarizes how epithelial-driven immune interactions link exacerbation-associated inflammation to persistent epithelial dysfunction and disease progression in COPD.

## IV Role of airway epithelial cells in lung repair

Lung tissue has an intrinsic capacity for repair that is activated after injury to restore epithelial integrity and lung function. This process is initiated by airway epithelial progenitor cells and relies on tightly regulated programs of proliferation, differentiation, and re-establishment of the epithelial barrier.^182^^,^^183^ In the healthy lung, conserved signaling pathways, including Wnt/*β*-catenin, Notch, and Hedgehog, coordinate epithelial regeneration and prevent aberrant remodeling.^184^, ^185^, ^186^, ^187^ During COPD exacerbations, however, infection-driven inflammation and repeated epithelial injury disrupt these repair programs. As a result, epithelial regeneration becomes incomplete and maladaptive, promoting pathological airway remodeling and progressive loss of lung function. Although acute inflammatory signals may transiently support epithelial repair, persistent inflammation characteristic of COPD ultimately impairs epithelial recovery and contributes to long-term structural damage.^16^^,^^45^^,^^156^^,^^188^

### A Cell types in lung repair and regeneration

Lung repair and regeneration depend on the coordinated activity of multiple epithelial progenitor populations that collectively restore barrier integrity and tissue architecture after injury.^189^, ^190^, ^191^ However, in COPD and particularly during acute exacerbations, these repair mechanisms are frequently disrupted. Recurrent injury and chronic inflammation alter the behavior and function of these cells, thereby compromising the lung’s capacity for effective regeneration.^188^

Within the airway epithelium, nonciliated club cells, formerly known as Clara cells, function as facultative progenitors. Under homeostatic conditions, they remain largely quiescent, but after injury, club cells proliferate and differentiate into ciliated or basal cells to restore the mucociliary barrier, subsequently returning to quiescence once homeostasis is re-established.^183^^,^^192^ In COPD, club cell depletion and reduced levels of Club Cell Secretory Protein (CCSP/CC16) are observed, and lower CCSP/CC16 levels correlate with increased disease severity and exacerbation risk, reflecting impaired epithelial regenerative reserve.^193^, ^194^, ^195^

Basal cells constitute another major regenerative population in the airway epithelium. During homeostasis, their activity is limited because of low epithelial turnover. However, with chronic exposure to irritants such as cigarette smoke, airway basal cells are among the earliest affected. A subset of basal progenitors loses regenerative capacity, leading to epithelial injury, insufficient repair, and amplified inflammation.^196^^,^^197^ Among basal-derived progenitors, P63^+^ progenitor cells possess self-renewal and multipotent differentiation capacity and contribute to the repair of lung injury.^198^^,^^199^ Although their function is impaired in COPD, residual P63^+^ cells can still be detected in bronchoscopic samples from patients with stage IV COPD.^200^ After extensive distal lung injury, these progenitor cells can migrate and participate in alveolar regeneration.^201^^,^^202^ These features position P63^+^ progenitor cells as a potential source for regenerative repair.

Although this review primarily focuses on airway epithelial regeneration, alveolar epithelial cells are equally critical for tissue repair.^203^ Alveolar type II (AT2) cells self-renew and differentiate into alveolar type I (AT1) cells to restore the gas exchange surface.^204^^,^^205^ In COPD, despite increased AT2 proliferation, transition toward AT1 differentiation is impaired, with cells persisting in an AT2-AT1 transition state that compromises alveolar repair.^206^^,^^207^ This arrested differentiation compromises effective alveolar repair and contributes to structural remodeling, particularly during exacerbation-associated inflammation.

Fibrocytes are bone marrow–derived mesenchymal progenitor cells that interact closely with airway epithelial cells and contribute to lung repair and structural remodeling. Within lung tissue, fibrocytes can differentiate into fibroblasts and myofibroblasts, thereby participating in epithelial repair and ECM remodeling.^208^^,^^209^ In COPD, chronic inflammation and cigarette smoke exposure induce functional abnormalities in these cells, thereby weakening epithelial regeneration and promoting EMT-associated remodeling.^210^, ^211^, ^212^, ^213^ During acute exacerbations, circulating fibrocyte numbers increase and correlate with disease severity.^214^^,^^215^ Although fibroblasts in COPD exhibit an impaired baseline capacity to support epithelial repair, exacerbation-associated inflammatory fluctuations may transiently modify epithelial–mesenchymal signaling and alter fibroblast-derived repair cues.^188^

Overall, these epithelial and mesenchymal progenitor populations form an integrated regenerative network; however, in COPD, particularly during exacerbations, their coordinated repair capacity is disrupted, leading to defective tissue restoration and progressive structural remodeling.

### B Airway epithelial cells as regulators of lung repair

Lung repair and regeneration after injury is a highly coordinated process driven by epithelial progenitor cells, which are important in restoring tissue integrity and function. This process is governed by intricate signaling pathways that regulate progenitor activation, proliferation, and differentiation.^216^ During COPD exacerbations, these repair mechanisms are often disrupted, leading to impaired regeneration of the airway epithelium and contributing to disease progression.

#### 1 Proliferation and differentiation of epithelial cells

Given the complexity of airway regeneration, epithelial cells exhibit remarkable plasticity, allowing them to change fate in response to damage. In certain contexts, epithelial cells that express markers of one differentiated lineage can switch phenotypes and give rise to alternative cell types. Lineage-tracing experiments suggest that this phenotypic switching may involve dedifferentiation into a multipotent intermediate, followed by redifferentiation into another cell type.^217^ Although this plasticity supports repair under physiological conditions, in COPD, particularly during exacerbations, persistent inflammation, infection, and oxidative stress can impair this adaptive response, leading to aberrant or incomplete regeneration. Disrupted differentiation and altered signaling under exacerbation conditions contribute to sustained epithelial dysfunction, excessive mucus production, and chronic airway remodeling.

In the airways, epithelial renewal is primarily mediated by progenitor populations such as club and basal cells. During repair, basal cells proliferate at the wound edge to compensate for cell loss, a process regulated by soluble factors released by resident and infiltrating inflammatory cells.^218^ Among these factors, members of the EGF and transforming growth factor (TGF) families are necessary in promoting epithelial proliferation via the activation of the epidermal growth factor receptor (EGFR).^15^

Dysregulation of the EGFR pathway, leading to aberrant EGFR signaling, has been linked to the pathogenesis of COPD.^219^ Both in asthma and COPD, sustained EGFR activation is associated with metaplastic and hyperplastic changes in the airway epithelium.^220^^,^^221^ Additionally, compared with healthy individuals, airway epithelial cells in COPD exhibit abnormal EGFR activation, which leads to an increased secretion of IL-8.^222^ Despite these findings, data on EGFR alterations during COPD exacerbations remain limited, highlighting the need for further research to explore its role in disease and exacerbation progression.

MMPs, particularly MMP-9, are essential in activating EGFR, by processing EGF and EGF-such as ligands into their active forms.^15^ Increased MMP-9 activity has been observed in the sputum and serum of patients with COPD, with common irritants such as acrolein, a constituent of cigarette smoke, enhancing MMP-9 expression and activity in airway epithelial cells.^223^, ^224^, ^225^ Chronic cigarette smoke exposure upregulates MMP-9, which in turn amplifies EGFR signaling, potentially leading to aberrant repair responses.^15^ Notably, during COPD exacerbations, increased MMP-9 levels in BALF and sputum indicate that MMP-9-induced EGFR hyperactivity may further impair normal airway regeneration.^102^^,^^226^

Notch signaling is also crucial for epithelial cell proliferation, differentiation, and apoptosis.^227^ The balance between Wnt/*β*-catenin and Notch signaling is crucial in determining cell fate, with Notch inhibition being associated with epithelial differentiation.^228^ Notch signaling plays a role in directing basal stem cell differentiation during airway epithelial repair. Notch activation promotes the differentiation of epithelial progenitors into secretory cell types, such as club and goblet cells. In the absence of Notch signaling, basal cells fail to differentiate in airways. Moreover, in airways, Notch inhibition after injury leads to an increase in ciliated cells at the expense of secretory cells, highlighting the pathway’s context-dependent effects on epithelial cell fate.^229^ The Notch signaling pathway is upregulated in the lung tissue samples from patients with COPD.^230^ Using an air–liquid interface (ALI) culture model, it was shown that exposure to CSE for 7 days activates Notch3 signaling in primary human bronchial epithelial cells (HBECs), which promotes goblet cell differentiation in the airway epithelium, contributing to chronic mucus hypersecretion.^60^ These signaling changes contribute to the repair processes of the lung parenchyma. Elevated Notch1 activity promotes goblet cell metaplasia by downregulating Forkhead Box A2 (FOXA2), a transcription factor essential for the late stages of epithelial cell differentiation.^231^ The loss of FOXA2 results in goblet cell hyperplasia, and during COPD exacerbations, microbial infections, and inflammation further suppress FOXA2 levels, exacerbating goblet cell hyperplasia and metaplasia.^232^ Additionally, SAM pointed domain ETS transcription factor, a key regulator of goblet cell differentiation in chronic lung diseases, is found at elevated levels in the lung tissue of patients with COPD.^233^ Increased SAM pointed domain ETS transcription factor expression is correlated with goblet cell metaplasia and excessive mucus production, which contribute to airway obstruction and worsening respiratory symptoms. Moreover, SAM pointed domain ETS transcription factor overexpression negatively impacts FOXA2 levels, further highlighting the intricate regulatory interplay between these transcription factors in airway epithelial differentiation.^234^ Beyond goblet cell differentiation, Notch-related dysregulation affects ciliary function in COPD through Forkhead Box J1, a key factor in ciliary assembly in airway epithelial cells.^15^ Forkhead Box J1 levels are lower in COPD compared with normal airway epithelial cell culture.^62^

Airway epithelial progenitor cells are essential for lung repair and regeneration through their ability to proliferate and differentiate into specialized epithelial cell types that restore barrier function. In COPD, this regenerative capacity is impaired by chronic inflammation, disrupted signaling, and repeated injury, leading to defective repair and abnormal cell fate decisions. Exacerbations worsen this dysfunction by amplifying inflammation and tissue damage, which may further alter progenitor cell behavior and fate. A better understanding of how these cells respond to injury and inflammation, especially during and after exacerbations, could help identify new treatments to restore epithelial integrity and slow disease progression.

#### 2 Epithelial-to-mesenchymal transition

EMT contributes to airway epithelial repair and regeneration after injury and is characterized by the loss of epithelial markers such as ZO-1 and E-cadherin, together with the acquisition of mesenchymal features, including upregulation of MMPs and increased S100A4 expression.^235^, ^236^, ^237^ Although this process facilitates cell migration and ECM remodeling, its dysregulation in COPD leads to aberrant epithelial repair.^69^^,^^238^^,^^239^ Patients with frequent exacerbations exhibit reduced levels of ZO-1 and E-cadherin, and acute exacerbations are associated with elevated MMP-9 levels in BALF.^66^^,^^226^ Chronic cigarette smoke exposure and recurrent infections disrupt ECM homeostasis, resulting in an imbalance between MMPs and their inhibitors, which further impairs repair capacity. EMT-related signaling is abnormally regulated in COPD, particularly alterations in TGF-*β* and Wnt/*β*-catenin pathways that are linked to defective epithelial regeneration.^240^ Evidence suggests that dynamic imbalance in these pathways diminishes regenerative potential and promotes pathological remodeling.^241^, ^242^, ^243^

**Fig. 2:**
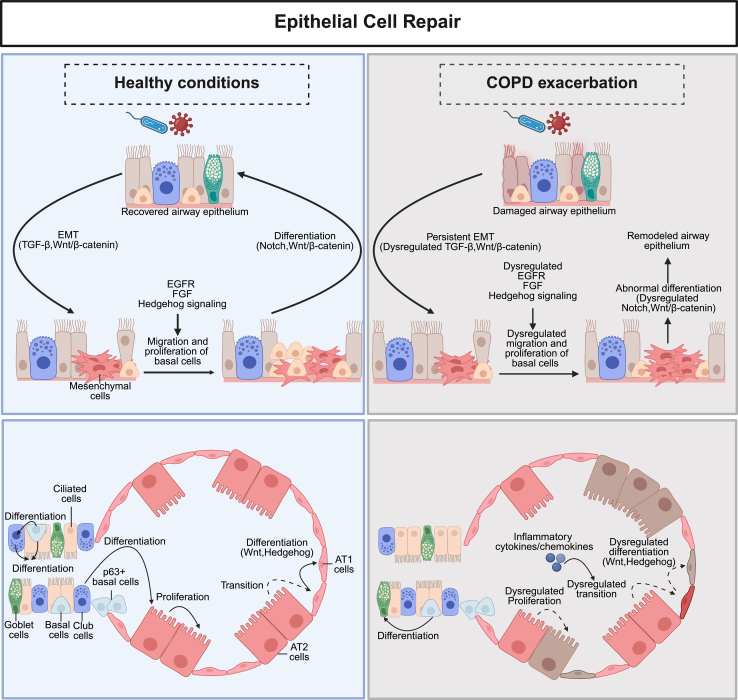
Epithelial repair processes in both healthy conditions and COPD exacerbation. In healthy conditions, epithelial cells contribute to tissue repair via EMT, driven by TGF-*β* and Wnt/*β*-catenin pathways, which facilitate the migration and proliferation of basal cells through EGFR, fibroblast growth factor (FGF), and Hedgehog signaling. Subsequently, these cells differentiate into airway epithelial cells via Notch and Wnt/*β*-catenin pathways. Conversely, during COPD exacerbations, persistent EMT driven by dysregulated pathways results in disrupted cell migration, proliferation, and abnormal differentiation, ultimately leading to a remodeled airway epithelium. In healthy conditions, the alveolar epithelial dynamics involve AT2 cells undergoing proliferation, followed by transition, and differentiation into AT1 cells, facilitated by Wnt and Hedgehog signaling pathways. In contrast, during COPD exacerbations, AT2 cell proliferation becomes dysregulated, the transition to AT1 cells is disrupted, and differentiation is altered, driven by the same signaling pathways but modulated by inflammatory cytokines.

Taken together, airway epithelial repair is fundamentally altered during COPD exacerbations compared with healthy conditions. Figure 2 provides a conceptual summary of the epithelial repair programs described above, contrasting coordinated regeneration in healthy lungs with disrupted repair during exacerbations. Under physiological conditions, epithelial repair is tightly regulated, with controlled EMT, basal cell migration and proliferation, and balanced differentiation governed by EGFR, Notch, Wnt/*β*-catenin, and Hedgehog signaling, leading to restoration of airway and alveolar structure. In contrast, during COPD exacerbations, persistent inflammatory signaling and pathway dysregulation sustain aberrant EMT, impair progenitor cell differentiation, and disrupt AT2-to-AT1 transition, resulting in defective epithelial repair and pathological airway remodeling.

### C The link between inflammation and lung repair in chronic obstructive pulmonary disease exacerbations

Inflammation plays a dual role, necessitating a delicate balance between facilitating repair and preventing further tissue damage. Although excessive inflammation is harmful to lung tissue, inflammatory cells are essential for regeneration, assisting in pathogen clearance and the removal of apoptotic and necrotic debris.^244^ However, during COPD exacerbations, bacterial or viral infections intensify inflammation, disrupting this delicate balance and potentially exacerbating lung injury. Persistent inflammation, combined with oxidative stress and a disrupted protease–antiprotease balance, further impair regenerative mechanisms, leading to defective lung repair and progressive tissue destruction.^36^^,^^245^ Key factors such as NLRP3 inflammasome activation, the formation of tertiary lymphoid structures, and alterations in prostaglandin signaling contribute to these processes, highlighting their significance in both inflammation regulation and disease progression.

A study involving 32 smokers, 65 patients experiencing an acute COPD exacerbation, 50 COPD recovery phase patients, and 30 stable patients with COPD demonstrated that NLRP3 inflammasome activation in both systemic circulation and local airways is strongly associated with acute COPD exacerbations.^246^ Recurrent acute exacerbations in COPD, often triggered by respiratory viruses or bacterial infections, further activate the NLRP3 inflammasome pathway in pulmonary epithelial cells.^247^ Although our review focuses on the airway epithelium, it is worth noting that a recent study using A549 alveolar epithelial cells demonstrated that combined exposure to CSE and lipopolysaccharides (LPS) significantly increased NLRP3 activity compared with CSE alone, highlighting the relevance of this pathway during exacerbation-like conditions in the alveolar compartment as well.^248^ Activation of the nuclear factor-*κ*B signaling pathway upregulates the transcription of NLRP3, pro-IL-1*β*, and pro-IL-18, thereby priming the inflammasome for subsequent activation. Upon assembly, caspase-1 mediates the cleavage and maturation of IL-1*β* and IL-18, which in turn amplify epithelial inflammatory responses and influence lung tissue repair processes.^249^^,^^250^ Moreover, NLRP3 has been shown to interact with the Wnt/*β*-catenin pathway, which regulates epithelial progenitor activation and differentiation during lung regeneration.^251^ Findings from lung stem/progenitor cell–derived organotypic models indicate that NLRP3 plays a role in airway epithelial repair and regeneration. Using murine lung stem/progenitor cell–derived organotypic models, including 2-dimensional ALI and 3-dimensional organoid cultures, experimental evidence has shown that the NLRP3 inflammasome exerts a context-dependent role in airway epithelial repair.^251^ Although early activation of the NLRP3 inflammasome promotes stem/progenitor cell migration and recruitment, aiding tissue repair and functional recovery, prolonged activation has detrimental effects. Persistent NLRP3 activity leads to chronic inflammation, stem/progenitor cell exhaustion, and ultimately, impaired regeneration and abnormal epithelial remodeling. Notably, blocking prolonged NLRP3 activation mitigates excessive epithelial cell death and prevents defective lung repair.^252^

In COPD, chronic inflammation leads to an excessive influx of activated immune cells into the airways, resulting in the continuous release of ROS and proteases. In the airways of patients with COPD, sustained production of ROS, primarily originating from the mitochondrial respiratory chain, contributes to persistent oxidative stress and chronic airway inflammation.^253^^,^^254^ Environmental stressors such as cigarette smoke further elevate mitochondrial ROS levels, leading to mitochondrial dysfunction and the acceleration of cellular senescence, an established pathological feature in COPD.^255^, ^256^, ^257^ Moreover, mitochondrial dysfunction not only contributes to chronic damage but is also closely associated with disease exacerbations. Consistently, mitochondrial alterations, including changes in circulating mitochondrial signals, have been linked to COPD exacerbations, further supporting the association between mitochondrial dysfunction and disease severity.^258^ Repeated cigarette smoke-mediated injury leads to cellular senescence and exhaustion in lung progenitor and stem cells, which compromises proper tissue repair.^259^ The oxidative environment resulting from chronic mitochondrial dysfunction and cigarette smoke exposure aggravates the protease–antiprotease imbalance, thereby impairing lung tissue repair mechanisms in COPD.^260^ Initially, protease levels substantially exceed those of their inhibitors, leading to degradation of essential structural components such as elastin and collagen. This imbalance contributes to the cleavage of enzymes, cytokines, receptors, and immune regulatory proteins, including complement and immunoglobulins.^261^ During exacerbations of COPD, increased levels of ROS lead to elevated levels of proteases and promote the degradation of mucins.^103^^,^^262^ Interestingly, contrary to expectations, in the early stages of acute exacerbations of COPD, despite intensified inflammation, the body temporarily counteracts excessive protease activity by reducing protease levels and increasing antiprotease levels, thereby limiting tissue damage.^54^ However, as exacerbations persist or inflammation further worsens, this protective balance gradually collapses. Although the overall protease levels remain low, the weakening of antiprotease inhibition leads to a relative increase in protease activity, resulting in enhanced proteolytic activity, accelerated ECM degradation, and ultimately more severe epithelial damage.^263^

In COPD, both T and B cells infiltrate lung tissue and contribute to the formation of tertiary lymphoid structures, which are associated with chronic inflammation and disease progression.^189^ Tertiary lymphoid structures are ectopic lymphoid aggregates that resemble secondary lymphoid organs and form in nonlymphoid tissues during chronic inflammation, where they facilitate local antigen presentation and adaptive immune responses.^264^ Tertiary lymphoid structures are observed during lung tissue destruction and emphysema in both humans^265^^,^^266^ and animal models, including mice.^267^, ^268^, ^269^ During COPD exacerbations, T cells may extravasate to sites of inflammation or organized lymphoid tissue.^270^ This heightened immune response may lead to an increase in the number and size of tertiary lymphoid structures, forming more prominent organized inflammatory centers in the lungs. Meanwhile, the cytokine, lymphotoxin *β*, released by CD8^+^ cells and its receptor signaling pathway, play a crucial role in the maintenance of tertiary lymphoid structures and the ongoing damage to lung tissue.^245^ Importantly, blocking the lymphotoxin *β* receptor signaling pathway suppresses inflammation and promotes lung repair.^271^ However, the precise role of tertiary lymphoid structures in lung repair during and after COPD exacerbations remains poorly understood, representing a critical gap in current knowledge.

Lipid mediators, particularly prostaglandins, are involved in resolving inflammation and promoting tissue repair in COPD.^245^^,^^272^ Prostaglandin E2 (PGE2) levels are elevated in the airways of patients with COPD, and higher expression is associated with greater airflow limitation.^273^ Similarly, systemic PGE2 levels correlate with disease severity, while increased PGE2 levels in induced sputum are linked to more severe respiratory symptoms and frequent exacerbations.^272^ PGE2 has been shown to facilitate wound closure in an in vitro epithelial cell culture model (HBEC line 16HBE14o^−^), possibly through the regulation of cell spreading and migration after mechanical injury.^274^ Moreover, PGE2 has been demonstrated to counteract TGF-*β*-induced EMT, thus preserving the epithelial phenotype and supporting effective regeneration.^275^ In addition to its effects on airway epithelium, prostaglandin signaling also plays a key role in alveolar repair. In patients with COPD and cigarette smoke-exposed mice, expression of prostaglandin synthase and the prostaglandin I2 receptor is reduced, impairing these repair mechanisms. Notably, PGE2 and prostaglandin I2 support lung repair by promoting the function of epithelial progenitor cells both in vitro and in vivo.^245^ Collectively, these findings underscore the critical role of prostaglandin signaling in lung epithelial barrier repair and suggest that its dysregulation in COPD may contribute to defective epithelial regeneration and chronic airway remodeling.

Overall, COPD exacerbations intensify inflammation, oxidative stress, and immune cell recruitment, creating a hostile environment that disrupts repair mechanisms and accelerates lung function decline. Identifying strategies to modulate key inflammatory pathways, such as targeting NLRP3, tertiary lymphoid structures, prostaglandin signaling, and oxidative stress regulation, may offer promising approaches for reducing inflammation, promoting lung repair, preventing progressive lung tissue damage, and restoring lung homeostasis after COPD exacerbations.

## V Treatment of chronic obstructive pulmonary disease exacerbations

### A Current therapies to treat/prevent chronic obstructive pulmonary disease exacerbations

Management of acute COPD exacerbations requires a comprehensive strategy that combines pharmacological and supportive interventions to relieve symptoms and prevent complications. Currently, a range of medications and supportive treatments are available for managing COPD exacerbations. Systemic corticosteroids improve lung function and oxygenation during acute episodes, reduce the risk of treatment failure, and shorten the duration of hospital stays.^276^ However, their long-term or repeated use is limited by adverse effects such as fluid retention, hypertension, diabetes mellitus, adrenal suppression, osteoporosis, and an increased risk of fractures.^277^ Long-acting bronchodilators are effective in improving expiratory airflow in stable patients, thus minimizing lung hyperinflation during exacerbations and lowering the exacerbation frequency.^278^

Antibiotics are effective primarily in bacterial COPD exacerbations.^279^ A systematic review of 10 trials involving 917 participants showed that antibiotic therapy for moderate-to-severe bacterial COPD exacerbations reduced mortality by 77%, sputum purulence by 44%, and treatment failure by 53%.^280^ Notably, macrolide antibiotics, particularly azithromycin, represent an exception, as they also possess anti-inflammatory and immunomodulatory properties. These effects can alleviate airway inflammation, enhance antiviral responses, and reduce the frequency of exacerbations, providing additional benefits in COPD management.^281^^,^^282^ Additionally, during COPD exacerbations, noninvasive ventilation enhances gas exchange and acid–base balance, reduces the need for invasive ventilation, and consequently lowers pneumonia risk, hospital stay duration, and mortality.^283^ Moreover, vaccination significantly reduces the incidence of acute exacerbations in patients with COPD.^284^^,^^285^ Among these, influenza vaccination effectively prevents virus-induced exacerbations and significantly reduces the risk of hospitalization from acute respiratory infections in patients with COPD.^286^ In addition, pneumococcal vaccines (such as 13-valent pneumococcal conjugate vaccine and 23-valent pneumococcal polysaccharide vaccine) help reduce bacterial exacerbations associated with *S pneumoniae* infection.^287^

Current COPD treatments mainly relieve symptoms and reduce exacerbation frequency. However, they do not address the underlying impairment in lung repair.^288^ Exacerbations are characterized by periods of intense inflammation and tissue remodeling. Although these episodes contribute to disease progression, they also activate endogenous repair mechanisms, presenting a unique therapeutic opportunity. In healthy lungs, acute inflammatory responses triggered by infections or environmental insults initiate epithelial regeneration, restoring tissue integrity. However, in COPD, chronic inflammation, repeated injury, and an altered microenvironment disrupt these repair processes, leading to incomplete regeneration and a gradual decline in lung function.^10^^,^^289^, ^290^, ^291^ We hypothesize that exacerbations represent a unique window for therapeutic intervention, where repair pathways, although dysregulated, are already activated. By strategically targeting these processes during exacerbations, it may be possible to shift the balance from ongoing tissue damage toward regeneration. Approaches that fine-tune inflammatory resolution to prevent excessive tissue destruction, enhance progenitor epithelial cell function, restore epithelial barrier integrity, or maintain ECM integrity to create a regenerative niche could promote lung repair and structural recovery.

### B Future therapies to treat/prevent chronic obstructive pulmonary disease exacerbations

#### 1 Strategies to enhance airway epithelial barrier function

COPD exacerbations are closely associated with increased airway epithelial barrier permeability and excessive mucus secretion, both of which contribute to disease progression and poor patient outcomes.^45^^,^^292^, ^293^, ^294^ Protein kinase D (PKD) has emerged as a pivotal regulator in maintaining this barrier’s integrity. Notably, PKD3 activation has been implicated in airway barrier dysfunction, particularly during respiratory viral infection.^295^^,^^296^ Inhibiting PKD3 has demonstrated promising results in vitro, enhancing the resistance of airway epithelial cells, potentially through upregulation of CLDN1.^295^ Although CLDN1 has not been directly linked to COPD or exacerbations, the CLDN family plays a critical role in epithelial barrier integrity and may be relevant to COPD pathogenesis. In particular, CLDN4 and CLDN5 have been associated with disease severity and barrier dysfunction during exacerbations, making them promising targets for therapeutic intervention.^71^^,^^72^ In another study, synthetic double-stranded RNA, poly (I:C), induces barrier dysfunction and disassembly of AJC through a PKD-dependent mechanism, and PKD inhibitor attenuates poly (I:C)-induced permeability of cell monolayers.^297^ Additionally, PKD inhibitors have shown efficacy in blocking early replication of human *Rhinovirus*, highlighting their therapeutic potential in virus-induced COPD exacerbation management.^298^

Beyond PKD inhibition, other strategies have been explored to support epithelial barrier function. The A-kinase anchoring protein (AKAP) inhibitor, St-Ht31 peptide, counteracts CSE-induced disruptions in E-cadherin-mediated cell–cell contacts in bronchial epithelial cells, indicating potential therapeutic benefits for COPD.^70^ As epithelial barrier dysfunction caused by cigarette smoke contributes to COPD exacerbations, targeting AKAPs may also represent a promising therapeutic approach during these acute episodes. The histone deacetylase (HDAC) family plays a critical regulatory role in chronic inflammation and tissue repair, and its dysregulated expression has been closely associated with the pathogenesis of COPD. Recent studies have highlighted the crucial role of HDAC6, a cytoplasmic deacetylase involved in regulating cell motility, stress response, and epithelial integrity, in the dysregulation of epithelial function. Notably, CAY10603, a potent small-molecule HDAC6 inhibitor, has demonstrated significant protective effects in both cigarette smoke-exposed mouse models and CSE-induced HBECs models. In vivo, CAY10603 attenuates small airway remodeling by improving alveolar structure, reducing collagen deposition, and reversing cigarette smoke-induced EMT via inhibition of the TGF-*β*1/Smad2/3 signaling pathway. In CSE-stimulated HBECs, CAY10603 significantly suppresses TGF-*β*1 release, restores E-cadherin expression, reduces *α*-smooth muscle actin levels, and upregulates tight junction proteins such as ZO-1 and occludin, thereby preserving epithelial barrier integrity and inhibiting cell migration.^299^ In addition to HDAC6, HDAC3 also plays a key role in maintaining alveolar epithelial barrier integrity, and its deficiency can prevent barrier disruption and acute lung injury by preserving mitochondrial quality control.^300^ Although current evidence mainly focuses on alveolar epithelial cells, HDAC3 inhibitors may also contribute to airway epithelial barrier maintenance, given the shared mechanisms involved in epithelial integrity. Taken together, AKAP and HDAC inhibitors, by targeting epithelial barrier disruption in both the airway and alveolar epithelium, hold promise as therapeutic candidates for treating COPD exacerbations.

Mucociliary clearance is essential for airway protection, with MUC5AC and MUC5B mucins playing key roles.^301^ Thiol derivatives, such as N-acetylcysteine (NAC), erdosteine, and carbocysteine, are commonly regarded as mucolytic agents and have been reported to significantly reduce the risk of exacerbations of COPD.^302^^,^^303^ NAC not only acts as a mucolytic agent but also functions as a mucoregulator, directly inhibiting mucus secretion and the proliferation of goblet cells.^304^ NAC has been shown to effectively inhibit the gene and protein expression of MUC5AC and MUC5B, reduce goblet cell numbers, and suppress excessive mucus secretion in both in vitro systems and COPD animal models.^305^, ^306^, ^307^, ^308^ However, clinical evidence remains mixed: earlier large-scale trials such as the Bronchitis Randomized on NAC Cost-Utility Study failed to show significant benefit in reducing exacerbation rates.^309^ In contrast, a subsequent multicenter, randomized, double-blind, placebo-controlled, parallel-group trial (ChiCTR-TRC-09000460) evaluated the preventive efficacy of NAC in patients with moderate-to-severe COPD and demonstrated that NAC can effectively reduced exacerbation frequency.^310^ In addition, an ongoing double-blind, randomized controlled trial (NCT05706402) is currently investigating the therapeutic efficacy of NAC in patients experiencing acute exacerbations of COPD.^311^ This study may provide important insights into whether NAC can also serve as an effective intervention during exacerbation episodes. These findings suggest that NAC holds potential not only for prevention but also as an interventional treatment during acute exacerbations. Further clinical validation is needed to determine its optimal application in COPD management.

Given the critical role of aberrant Notch3 signaling in mucus dysregulation and airway remodeling, *γ*-secretase inhibitors targeting the Notch pathway have emerged as a potential therapeutic strategy.^57^, ^58^, ^59^ For instance, LY450139 has been shown to reverse Notch-mediated loss of multiciliated cells and restore epithelial structure.^59^ LY450139 has demonstrated good safety and differentiation-promoting effects in both in vitro human nasal epithelial cell cultures and in vivo mouse models, showing dose-dependent increases in multiciliated cell numbers and reductions in IL-13–induced goblet cell hyperplasia.^59^ Therefore, *γ*-secretase inhibitors, as inhibitors of the Notch signaling pathway, hold therapeutic potential to restore epithelial integrity and suppress goblet cell hyperplasia, making them promising candidates for maintenance therapy and prevention of airway remodeling during COPD exacerbation.

Epithelial barrier dysfunction and mucus hypersecretion are central to COPD exacerbations and are closely linked to airway inflammation. These processes interact dynamically, reinforcing each other and contributing to disease severity. Therefore, therapeutic strategies should also focus on modulating the inflammatory response to effectively restore epithelial function.

#### 2 Regulating airway epithelial inflammation

As previously discussed, the NLRP3 inflammasome is an important element of the inflammatory response, showing significant involvement in both stable COPD and its exacerbations.^120^^,^^246^ Targeting the NLRP3 inflammasome pathway has emerged as a promising strategy to mitigate airway inflammation and reduce the frequency of COPD exacerbations. In support of this, preclinical evidence indicates that andrographolide, a natural compound, can suppress NLRP3 inflammasome activation and alleviate airway inflammation during exacerbations.^312^ Selnoflast, an orally available NLRP3 inhibitor, has completed a phase I clinical trial for treating cryopyrin-associated periodic syndrome (NCT04086602)^313^ and was subsequently investigated by Roche for its potential in treating COPD. A Phase Ib randomized, double-blind, placebo-controlled clinical trial was conducted to evaluate the safety, tolerability, and pharmacokinetics of RO7486967 (Selnoflast) in this population. Only one patient was enrolled and completed the study, with no adverse events or clinically significant changes observed. Because of the extremely limited enrollment, no efficacy or pharmacokinetic analyses were performed. The study was terminated early to reassess the development strategy of Selnoflast for COPD treatment. Although clinical development was halted at an early stage, Selnoflast and other NLRP3 inflammasome inhibitors remain promising candidates for future COPD therapies, particularly in alleviating acute airway inflammation, controlling the frequency of COPD exacerbations, and possibly providing long-term maintenance or preventive benefits.

P38 mitogen-activated protein kinase is a regulator of immune and inflammatory responses, closely linked to airway and lung inflammation in COPD. During COPD exacerbations, bacterial and viral triggers enhance p38 mitogen-activated protein kinase activity. This activation increases cytokine production, promotes epithelial apoptosis, and weakens bronchial barrier function, thereby amplifying airway inflammation.^9^^,^^314^, ^315^, ^316^ The p38*α* isoform is a critical node in multiple inflammatory signaling pathways, regulating and activating key proinflammatory mediators.^317^ In patients with COPD, p38*α* activation levels are increased compared with healthy controls and are associated with lung function impairment and the extent of alveolar wall inflammation.^318^ Although p38*α* inhibitors show anti-inflammatory effects in cigarette smoke-exposed murine models,^319^ and reduced cytokine production in alveolar macrophages and bronchial epithelial cells of patients with COPD, long-term treatment with oral p38*α* inhibitors have not significantly improved clinical outcomes in patients with COPD.^320^ This may be due to “inflammatory escape” during prolonged medication use, evidenced by the decline in positive effects on inflammatory markers within weeks despite continuous dosing.^321^, ^322^, ^323^ Therefore, inhibiting p38 may be best suited for the acute, short-term treatment of inflammation-driven disease exacerbations, such as acute exacerbations of COPD. The oral p38*α* inhibitor BCT-197 (acumapimod) holds promise as a treatment for acute exacerbations of COPD (NCT01332097).^324^ The side effects and dose-limiting systemic toxicities of oral p38 inhibitors, such as increased liver enzymes and rashes, necessitate the development of new inhaled p38*α* inhibitors that deliver the drug directly to the lungs to minimize systemic exposure and related side effects.^325^ Inhaled p38*α* inhibitors such as AZD7624 have not shown the expected results in trials for the treatment of COPD and prevention of exacerbations,^326^ but the new inhaled inhibitor CHF6297 demonstrates anti-inflammatory properties and is suitable for use as a dry powder inhaler.^327^ In human peripheral blood mononuclear cells stimulated with LPS, as well as in HBECs (BEAS-2B) stimulated with TNF-*α* or CSE, CHF6297 inhibited IL-8 release with low nanomolar potency.^327^ Furthermore, as a dry powder formulation, CHF6297 demonstrates good lung retention in rats and effectively reduces the infiltration of neutrophils in the airways induced by LPS and cigarette smoke exposure.^327^ These results indicate that CHF6297, as an inhaled therapeutic agent, has potential in managing COPD and other acute inflammatory lung conditions. The potent anti-inflammatory effects of CHF6297 have also been confirmed in another acute pulmonary inflammation model triggered by IL-1*β*, a cytokine that typically elevates during inflammasome activation and acute exacerbations of COPD.^327^ Given the promising but complex role of p38*α* inhibitors in COPD management, particularly the potential of CHF6297 as an inhaled agent with strong anti-inflammatory activity, future research should focus on optimizing short-term dosing strategies and evaluating their efficacy specifically during acute exacerbations.

PDEs are enzymes that regulate the metabolic breakdown of cyclic adenosine monophosphate and cyclic guanosine monophosphate, important secondary messengers involved in numerous cellular functions.^328^ PDEs, by regulating the levels of cyclic adenosine monophosphate and cyclic guanosine monophosphate, play a critical role in the complex interactions among various cell types, inflammatory mediators, and signaling pathways in the pathogenesis of COPD.^328^^,^^329^ Ensifentrine, a novel inhaled PDE3/4 inhibitor, regulates cyclic adenosine monophosphate and cyclic guanosine monophosphate levels in the bronchial tissues of patients with COPD by inhibiting PDE isoenzymes, thereby reducing the recruitment of inflammatory cells and downregulating the inflammatory response.^330^, ^331^, ^332^ Ensifentrine enhances the quality of life for patients with COPD, improves lung function, and reduces exacerbation rates.^333^^,^^334^ Compared with patients with COPD who rely on inhalation maintenance therapy (such as bronchodilators and inhaled corticosteroid) for long-term symptom control, the direct pulmonary administration of ensifentrine enhances the local therapeutic effect in the lungs (ie, bronchodilation and anti-inflammatory effects) through dual inhibition of PDE3/4, while reducing the systemic side effects that traditional oral PDE3 and PDE4 inhibitors might cause.^335^ Ensifentrine has recently completed 2 phase III clinical trials (NCT04535986 and NCT04542057) for the maintenance treatment of COPD.^336^ These 24-week, multicenter, randomized, double-blind, placebo-controlled studies evaluated the efficacy of twice daily 3 mg ensifentrine inhalation in patients aged 70–80 years with severe COPD who had stable symptoms for over a decade. A significant reduction in the annualized rate of moderate-to-severe COPD exacerbations and a delayed time to the first exacerbation within 24 weeks were observed. After these trials, ensifentrine (OHTUVAYRE) received US Food and Drug Administration approval in June 2024 for the maintenance treatment of in adult patients with COPD in the United States.^337^ However, although its efficacy as a maintenance therapy is now established, its therapeutic value during acute exacerbation episodes remains unclear and warrants further investigation.^337^

Inflammation in COPD exacerbation, particularly type 2 inflammation, often involves overactivity of IL-4 and IL-13, which can worsen symptoms and exacerbate disease progression.^126^ By inhibiting IL-4, treatments can effectively reduce this harmful overactivity. Dupilumab is an inhibitor of IL-4R*α*, thereby blocking both IL-4 and IL-13 signaling pathways, which are critical in type 2 inflammation.^140^ Recently, dupilumab has shown promising results in COPD treatment, preventing exacerbations. In 2023, phase III clinical trial, the BOREAS trial (NCT03930732), evaluated the efficacy and safety of dupilumab in patients with moderate-to-severe COPD.^20^ In this study, 939 participants were randomly assigned to receive dupilumab or a placebo. There was a significant reduction in the annual incidence of moderate-to-severe COPD exacerbations and a notable improvement in FEV1 at 12 and 52 weeks compared with placebo. In 2024, the independently conducted NOTUS trial served as a replication of the BOREAS study and confirmed its findings, with similarly positive effects on exacerbation reduction and lung function improvement. Together, these findings establish dupilumab as a promising therapeutic option for reducing exacerbations in patients with COPD with type 2 inflammation. Although dupilumab was approved by the US Food and Drug Administration in 2024 for COPD treatment, its effectiveness during acute exacerbation events remains unstudied, highlighting the need for further research into its role in acute COPD management.^338^

In addition to IL-4, other epithelial-derived cytokines, such as TSLP and IL-33, are involved in type 2 immune responses.^339^ TSLP and IL-33 are overexpressed in airway biopsies from patients with COPD and are associated with disease severity.^340^ A multicenter, randomized, double-blind, placebo-controlled, parallel-group phase IIa trial (NCT04039113) evaluated the efficacy and safety of the anti-TSLP monoclonal antibody tezepelumab as a therapy in patients with moderate-to-severe COPD. Although the study did not show a significant reduction in the rate of moderate or severe exacerbations, it demonstrated improvements in prebronchodilator FEV1. Tezepelumab was well tolerated, with an adverse event profile comparable to placebo.^341^ Building on these findings, an ongoing phase II trial (NCT05507242) is currently investigating the effects of tezepelumab on airway inflammation and epithelial immune responses in patients with COPD. This multicenter, randomized, double-blind, placebo-controlled study has enrolled approximately 80 patients with at least one acute exacerbation in the past 12 months, all receiving standard maintenance therapy. Over a 20-week treatment period, participants receive subcutaneous injections of tezepelumab or placebo, with the aim of evaluating the therapeutic potential of TSLP blockade in modulating airway inflammation and immune responses to exacerbation triggers. However, the results of this trial have not yet been published, and the role of tezepelumab in preventing COPD exacerbations remains under investigation.

Tozorakimab is the first monoclonal antibody developed to target IL-33, and its inhibitory effects have been confirmed in preclinical studies. Tozorakimab can effectively reduce the concentration of eosinophils and inflammatory biomarkers, such as IL-5 and IL-13, in the blood of patients with mild airway obstruction.^342^ In its first human clinical trial (NCT03096795), which included healthy volunteers and patients with mild airway obstruction, the drug demonstrated good safety and tolerability, with linear, time-independent pharmacokinetic properties in serum and a low incidence of antidrug antibodies.^343^ A phase II, multicenter, randomized, double-blind, placebo-controlled study (NCT04631016) designed to assess the efficacy, safety, and tolerability of the anti–IL-33 monoclonal antibody MEDI3506 (tozorakimab) in participants with moderate-to-severe COPD and chronic bronchitis. Preliminary results indicate that tozorakimab may enhance lung function and reduce the frequency of COPD exacerbations, especially in individuals with a history of frequent exacerbations.^344^ Phase III studies (NCT05158387 and NCT05166889) are ongoing to evaluate whether tozorakimab, administered via subcutaneous injection, can reduce the frequency and severity of exacerbations by modulating inflammation. Patients with a history of moderate-to-severe COPD exacerbations are being evaluated for improvements in lung function, exacerbation rates, and quality of life.^345^^,^^346^

Itepekimab, another anti–IL-33 monoclonal antibody, was recently tested in a phase II clinical trial (NCT03546907) for COPD. The results showed that it reduced exacerbation rates and improved lung function in former smokers but not in current smokers, prompting the initiation of ongoing phase III trials.^347^ Two phase III trials, AERIFY-1 (NCT04701983) and AERIFY-2 (NCT04751487), evaluated the efficacy and safety of the anti–IL-33 monoclonal antibody itepekimab in former smokers with moderate-to-severe COPD.^348^ AERIFY-1 met its primary endpoint, demonstrating a 27% reduction in the annualized rate of moderate or severe COPD exacerbations at week 52 compared with placebo. In contrast, AERIFY-2 did not meet this endpoint, despite early improvements at week 24. Overall, these findings indicate that itepekimab has variable efficacy but a consistently favorable safety profile in patients with moderate-to severe COPD.^349^

Astegolimab, a human IgG2 monoclonal antibody, inhibits the IL-33 receptor ST2, is well tolerated, and may help reduce the frequency of COPD exacerbations. It is currently being evaluated in patients with COPD who experience frequent exacerbations (NCT05878769, NCT05037929, and NCT05595642).^350^ Tozorakimab, itepekimab, and astegolimab are currently under investigation as preventive treatments for COPD exacerbations, particularly in patients with a history of frequent exacerbations.

In summary, inflammation constitutes a central mechanism underlying COPD exacerbations, involving both type 1 and type 2 immune responses. Therapeutic strategies targeting key inflammatory pathways have shown promising potential in both preclinical and clinical studies for the prevention and management of exacerbations. These findings underscore the value of precision medicine targeting inflammatory endotypes and support combining anti-inflammatory and epithelial repair strategies to improve clinical outcomes in COPD. Future studies should assess their efficacy during exacerbations and identify optimal timing and patient populations.

#### 3 Boosting lung repair/regeneration

Traditionally, it was believed that the adult human lung lacked regenerative capabilities. However, this notion has been reevaluated because of emerging case reports of lung regeneration after surgical resection.^351^ Although the cellular and molecular mechanisms underlying adult lung regeneration remain partially understood, recent studies employing animal models have identified stem/progenitor cell populations dispersed throughout the lung. These cells are capable of responding to injury and facilitating tissue repair.^352^^,^^353^ Previous studies have shown that cigarette smoke exposure can disrupt the normal differentiation of airway epithelial progenitor cells, impair the wound repair capacity of bronchial progenitors, and induce adverse remodeling toward secretory and squamous phenotypes.^354^^,^^355^ Building on this, our recent findings further demonstrate that in vivo cigarette smoke exposure also impairs the functionality of alveolar epithelial progenitors and reprograms transcriptional pathways critical for cellular homeostasis, proliferation, and inflammation.^245^

These insights have promoted new therapeutic strategies in COPD that aim to boost endogenous lung repair, especially after exacerbations,^356^^,^^357^ with the goal of restoring function and halting disease progression, a notable shift from purely symptom-focused treatments.

##### a Airway basal progenitor cell-based therapies

Airway basal cells are essential epithelial progenitor cells. In COPD, their aberrant differentiation contributes to airway remodeling and barrier dysfunction. Restoring their regenerative capacity may offer a new therapeutic strategy during and after acute exacerbations. A recent study investigated the effects of quercetin, a dietary flavonoid, on airway epithelial regeneration in patients with COPD (ClinicalTrials.gov identifier: NCT03989271). This study demonstrated that airway basal cells derived from patients with COPD regenerated abnormal epithelial structures in vitro, characterized by goblet cell hyperplasia and downregulation of key developmental genes, indicating impaired regenerative programming. However, quercetin treatment significantly improved epithelial polarization in ALI cultures, evidenced by an increased proportion of ciliated cells, reduced goblet cell numbers, and decreased IL-8 secretion. These reparative changes were closely associated with the upregulation of genes involved in epithelial development and differentiation. Importantly, bronchial brushings from patients with COPD who received oral quercetin for 6 months also showed significantly increased expression of key developmental genes, suggesting the translational therapeutic potential of quercetin in vivo.^358^ Overall, this study highlights the therapeutic value of targeting airway basal progenitor cell function and plasticity, particularly during COPD exacerbations and the recovery phase.

In addition, specific type of basal progenitor cells, P63^+^ cells, originate from the airway epithelium and can migrate to alveolar regions under conditions of COPD and other types of lung injury, where they play an important role in tissue repair and regeneration. Their ability to regenerate damaged epithelial structures makes these cells a promising target for therapeutic interventions aimed at alleviating the progressive damage characteristic of COPD. A phase I clinical trial (NCT03188627) evaluated the efficacy and safety of autologous P63^+^ progenitor cell transplantation in 28 patients with stage II to IV COPD. P63^+^ cells were isolated from the bronchial epithelium, cultured for 3–5 weeks, and transplanted back into the lung. Twenty patients completed the study (17 in the intervention group, 3 in the control group). The intervention group showed significant improvements in diffusing capacity of the lungs for carbon monoxide (+18.2%) and a >30 m increase in the 6-minute walk test. No grade 3–5 adverse events were observed, suggesting that this therapy may be a potential COPD treatment strategy.^199^ In a randomized, single-blind, controlled phase I/II trial, the safety and efficacy of autologous p63^+^ progenitor cell transplantation were investigated. A total of 37 patients underwent bronchoscopic airway clearance therapy (B-ACT) or B-ACT combined with p63^+^ progenitor cell therapy. Results indicated that the cell therapy group exhibited a significantly higher improvement in diffusing capacity of the lungs for carbon monoxide levels from baseline to 24 weeks after treatment compared with the control group. Additionally, patients in the cell therapy group demonstrated significant reductions in lung injury area, enhanced quality of life for patients, and improved severity of bronchiectasis between weeks 4 and 12 after treatment. Transcriptomic analysis showed that progenitor cells with higher p63 gene expression achieved better therapeutic outcomes (NCT03655808).^359^ These results highlight the transformative potential of progenitor epithelial cell therapy in addressing the complexities of COPD, offering a novel approach to enhancing lung repair after exacerbations and supporting long-term recovery and maintenance of lung function.

##### b Mesenchymal stem/stromal cell and mesenchymal stem/stromal cells**–**derived extracellular vesicles

Mesenchymal stem/stromal cells (MSCs) hold significant potential for lung regeneration due to their multidirectional differentiation capabilities, immunomodulatory functions, and anti-inflammatory properties. MSCs promote structural repair of the lungs by differentiating into pulmonary epithelial cells, reducing apoptosis, and modulating the inflammatory microenvironment.^360^, ^361^, ^362^, ^363^, ^364^ MSCs exist in a variety of tissues, such as bone marrow, adipose tissue, and umbilical cords. According to the different sources, these cells are respectively named as bone marrow–derived MSCs (BM-MSCs), adipose-derived MSCs (AD-MSCs), and umbilical cords-derived MSCs (UC-MSCs).^365^ The therapeutic potential of MSCs in COPD has been demonstrated through their ability to reduce inflammation, regulate oxidative stress, inhibit apoptosis, and potentially regenerate lung parenchyma.^366^, ^367^, ^368^, ^369^

A preclinical study demonstrated that human BM-MSCs can promote the repair of damaged small airway epithelium through multiple mechanisms in vitro. In this study, green fluorescent protein-labeled BM-MSCs were cocultured with small airway epithelial cells that had been subjected to thermal injury. The results showed that these BM-MSCs not only acquired epithelial-like morphology and expressed epithelial-specific marker genes, but also underwent cell fusion and even nuclear fusion with small airway epithelial cells. These findings suggest that BM-MSCs may contribute to epithelial repair via differentiation, cell fusion, and nuclear fusion, providing important mechanistic insights into their potential application in pulmonary regenerative medicine.^363^

In addition, another study established an in vitro coculture system for tracheal epithelial reconstruction to investigate the cellular and molecular interactions between BM-MSCs and normal human bronchial/tracheal epithelial (NHBE) cells. This system was based on a Transwell insert, with BM-MSCs cultured in the lower chamber and NHBE cells seeded in the upper chamber. Under ALI conditions in the coculture system, NHBE cells gradually differentiated into functional epithelium with ciliated and secretory phenotypes, mimicking the morphological and functional characteristics of the tracheal mucosa. Under coculture conditions, NHBE cells exhibited enhanced and sustained mucin secretion starting from day 18, which remained at a high level through day 25. In contrast, the monoculture group showed relatively low secretion on day 18, a sharp peak on day 21, followed by a significant decline by day 25, indicating limited durability of secretory function.^370^ These findings suggest that coculture with BM-MSCs not only promotes an earlier onset of epithelial secretory activity but also prolongs its maintenance, implying that epithelial–mesenchymal interactions may play a regulatory role in the differentiation and functional maturation of secretory epithelial cells.

The transition to clinical applications presents a critical step forward in assessing the efficacy of MSC therapies in human subjects with COPD. The first trial using MSCs in patients with moderate-to-severe COPD (GOLD II-III) was a prospective, randomized, double-blind, placebo-controlled trial in the United States for BM-MSCs in patients with COPD (ClinicalTrials.gov identifier: NCT00683722)^371^ and aimed to assess the safety and efficacy of MSC treatment and its effect on circulating inflammatory mediators. A total of 62 patients with moderate-to-severe COPD were randomly assigned to receive 4 monthly infusions of either allogeneic MSCs or placebo, followed for 2 years after the initial infusion. The study demonstrated the feasibility of allogeneic MSCs treatment without serious adverse reactions, and significant reductions in C-reactive protein levels among participants with initially high levels, though no significant improvements in clinical symptoms or lung function were observed. Another phase I prospective open-label trial (NCT01306513) evaluated the safety and feasibility of intravenous administration of BM-MSCs. Ten patients with severe COPD scheduled for lung volume reduction surgery (LVRS) were selected. BM-MSCs were administered via intravenous infusion starting 8 weeks postinitial LVRS, with weekly injections over 2 weeks. A second LVRS occurred a month later. Autologous BM-MSC therapy in patients with severe emphysema was found to be feasible and safe. After LVRS and MSC treatment, a threefold increase in the expression of the endothelial marker CD31 was observed, suggesting enhanced responsiveness of microvascular endothelial cells in the most severely affected regions of the lung.^372^

In a single-site, phase I clinical trial (Australian clinical trials identifier: 12614000731695), researchers investigated the in vivo distribution and systemic inflammatory response after intravenous infusion of allogeneic BM-MSCs.^373^ The trial involved 9 patients receiving 2 weekly infusions. The BM-MSCs quickly reached the lungs and were mainly found in the liver 24 hours postinfusion. Despite not significantly improving lung function, the study noted a short-term decrease in C-reactive protein levels, indicating a reduction in systemic inflammation. This transient response may be related to the limited residence time of BM-MSCs in the lungs, which could restrict their therapeutic efficacy.^374^ To overcome this limitation, a phase I, prospective, patient-blinded, randomized, placebo-controlled study (NCT01872624) further investigated the therapeutic potential of endobronchial valve implantation combined with intrabronchial MSC administration in patients with severe COPD. The treatment showed potential in reducing inflammation and improving the quality of life in patients with COPD, although improvements in lung function were limited.^375^ These findings highlight the importance of achieving efficient pulmonary distribution and prolonged retention of therapeutic agents or cells for sustained therapeutic benefits. Local administration routes, such as intratracheal or intrabronchial delivery, could further improve the efficacy of MSC-based treatments.

Compared with bone marrow, adipose tissue contains a higher concentration of MSCs with greater proliferative capability, longer-lasting differentiation potential, and stronger immunomodulatory functions.^376^, ^377^, ^378^, ^379^ A preclinical in vitro study using a Transwell coculture system investigated the effects of AD-MSCs on human small airway epithelial cells. The AD-MSCs significantly enhanced the viability of human small airway epithelial cells, suggesting a protective role against cell death. Coculture with AD-MSCs upregulated the expression of epithelial-specific markers such as mucin 1 and intercellular adhesion molecule 1 (ICAM1), indicating improved epithelial function. These findings suggest the therapeutic potential of AD-MSCs in promoting airway epithelial repair and modulating inflammatory responses.^380^ A case report on a 57-year-old patient with COPD receiving autologous AD-MSCs showed improvements in dyspnea, exercise capacity, and overall quality of life, though lung function improvements were modest.^381^ A phase III, multicenter, randomized, double-blind, placebo-controlled study is proposed to test the clinical safety and efficacy of AD-MSC therapy in patients with COPD.^382^ These findings highlight the therapeutic promise of AD-MSCs, yet larger, well designed randomized clinical trials are required to establish their efficacy, optimal dosing, and long-term safety in COPD treatment.

Compared with BM-MSCs and AD-MSCs, UC-MSCs exhibit superior regulatory capabilities.^383^ UC-MSCs show stronger immunosuppressive effects on allogeneic lymphocytes under the same conditions, have higher proliferation rates, and display greater potential for differentiation because of their more primitive nature.^384^ Therefore, UC-MSCs are a preferable choice for allogeneic stem cell transplantation. In a pilot clinical trial (ISRCTN70443938), researchers explored the use of UC-MSCs as a treatment for COPD.^385^ The study included 20 patients diagnosed with COPD classified as GOLD stage III or IV with high symptom burden and exacerbation risk, in accordance with GOLD 2016. All patients received allogeneic UC-MSCs intravenously (1.5 × 10^6^ cells/kg) and were followed for 6 months. No serious or clinically significant adverse events were observed. Although pulmonary function showed no statistically significant differences, modified Medical Research Council (mMRC) scores, COPD assessment test scores, and exacerbation frequency significantly decreased after treatment, indicating UC-MSCs can improve patients’ quality of life. This was the first clinical trial to use MSCs from umbilical cord tissue to treat patients with COPD. Then, a phase I/II clinical trial conducted in 2020 (NCT04433104) evaluated the safety and efficacy of allogeneic UC-MSC in patients with moderate-to-severe COPD. In this study, 40 patients were randomly assigned to receive UC-MSC infusions (1 × 10^6^ cells/kg) or a placebo. However, no official results have been posted on ClinicalTrials.gov to date.^386^ In an open-label, single-arm study, participants received 4 doses of UC-MSCs (1–2 × 10^6^ cells/kg) via intravenous infusion every 2 weeks.^387^ The study assessed respiratory function, St George’s Respiratory Questionnaire scores, and 6-minute walk tests. The treatment significantly improved patients’ quality of life and slightly enhanced lung function, with an increase in pretreatment to posttreatment FEV1/forced vital capacity ratios. This study used a more frequent dosing interval, demonstrating potential therapeutic benefits despite the small sample size and short follow-up, offering promise for future stem cell-based treatments for COPD. As of 2024, the NCT06491043 clinical trial is still ongoing and recruiting patients, with no results published to date. This phase II trial aims to assess the safety and efficacy of UMC119-06-05 in patients with moderate-to-severe COPD.^388^

Concerns about the risks associated with MSC therapy, such as tumorigenicity and immune responses, have led to exploration of alternative strategies such as using mesenchymal stem cell–derived extracellular vesicles (MSC-EVs) These EVs offer similar therapeutic benefits to MSCs, including safer control of regenerative properties without risks such as tumorigenesis or embolization, and advantages in storage and handling.^389^ MSC-derived EVs mimic many MSC functions by delivering bioactive peptides, proteins, and RNA to injured tissues and promoting regeneration.^390^ MSC-EVs play a crucial role in modulating the progression of inflammation and tissue repair processes.^391^ Moreover, in vivo experiments using mice models of acute lung injury induced by LPS or *Escherichia coli* have consistently demonstrated that intravenous administration of MSC-EVs significantly reduces pulmonary inflammation and edema, and improves disease outcomes. For example, Zhu et al^392^ showed that human MSC-derived microvesicles alleviated LPS-induced lung injury in mice, whereas another study confirmed the therapeutic effects of MSC-EVs in a mouse model of severe pneumonia.^393^ Moreover, in a CSE-induced COPD mouse model, bone marrow mesenchymal stem cells (BMSCs)-derived exosomes alleviated lung damage, potentially through the delivery of miR-30b, which targets Wnt5a to reduce apoptosis in pulmonary microvascular endothelial cells.^394^ Although direct clinical evidence in COPD exacerbations remains limited, the demonstrated ability of MSC-EVs to reduce inflammation, preserve epithelial integrity, and restore alveolar-capillary function in acute lung injury models strongly supports their potential relevance. Further research is needed to establish their therapeutic potential and translational feasibility in COPD exacerbation management.

The findings by Harrell et al^395^ represent a breakthrough in improving lung function of COPD, introducing a product known as “Exosome-Derived Multiallogenic Protein Paracrine Signaling (Exo-d-MAPPS).” This product contains MSC-derived exosomes and immune-regulatory factors (sTNFRI, sTNFRII, IL-1Ra, and sRAGE) that are involved in lung repair and regeneration. Exo-d-MAPPS demonstrated notable efficacy in reducing chronic airway inflammation and enhancing lung function in preclinical and clinical studies.^395^ The mechanism of action appears to involve the anti-inflammatory effects of these soluble mediators, which inhibit the influx of inflammatory leukocytes and promote the proliferation of lung immunosuppressive cells. Changes in lung cellular composition contribute to an anti-inflammatory microenvironment, enhancing tissue repair and regeneration, and improving lung function in animals exposed to cigarette smoke and patients with COPD.^395^ Therefore, Exo-d-MAPPS is considered a potential new therapeutic for treating COPD, and its efficacy should be further explored in large-scale clinical trials.

Together, MSCs (BM-MSCs, AD-MSCs, and UC-MSCs) support airway repair and inflammation resolution, particularly during COPD maintenance and recovery phases, whereas MSC-EVs, including Exo-d-MAPPS, offer therapeutic potential for both acute exacerbation treatment and postexacerbation repair.

##### c Alveolar type II cell-targeted therapies

Although this review primarily focuses on airway epithelial regeneration, the role of AT2 cells in lung repair is equally important. As key progenitor cells responsible for maintaining alveolar integrity and promoting regeneration after injury, AT2 cells represent a promising therapeutic target, particularly in COPD, where both airway and alveolar structures are compromised. Current research efforts are focused on identifying strategies to activate AT2 cell proliferation and differentiation to enhance alveolar repair and regeneration.^190^ A recent study showed that the activation of thyroid hormone receptor *β* directly regulates the expression of key transcription factors, such as Kruppel-like factor 2 and CCAAT-enhancer binding protein *α*, which synergistically drive the differentiation of AT2 cells into functional AT1 cells. This suggests that thyroid hormone receptor *β* agonists could be explored as therapeutic agents to promote alveolar repair.^396^ Moreover, increasing AT2 cell proliferation offers protective effects in rodent fibrosis models, suggesting that therapies aimed at restoring AT2 cells could broadly enhance lung repair.^397^^,^^398^ Dipeptidyl peptidase 4 (DPP4) inhibitors, commonly used for treating type 2 diabetes, can specifically promote the expansion of AT2 cells. This was determined by analyzing the impact of DPP4 inhibition on the pulmonary levels of IL-6 and insulin-like growth factor 1 (IGF-1).^399^ Inhibiting DPP4 activity resulted in an increase in these cytokines, enhancing the proliferation of AT2 cells. Notably, both IL-6 and IGF-1 have been identified as pivotal modulators of AT2 biology, with IL-6 also being recognized for its role in mitigating bleomycin-induced lung fibrosis and serving as a physiological mitogen for AT2.^397^^,^^400^ The disruption of IL-6 signaling exacerbated lung injury caused by bleomycin, indicating its essential role in lung repair processes.^397^^,^^401^ Similarly, IGF-1 facilitates the proliferation of alveolar epithelial cells during lung development, and its receptor IGF-1R is necessary for the proliferation and self-renewal induced by IGF-1 in AT2 cells.^397^^,^^402^^,^^403^ These findings underscore the potential of targeting DPP4 as a novel approach to enhancing alveolar repair, supported by the analysis of human lung atlas datasets, which confirmed the expression of DPP4, IL6R, and IGF-1R predominantly in canonical AT2 cells, highlighting the specificity of this regulatory mechanism in lung epithelial progenitor cells.^404^, ^405^, ^406^ Sitagliptin, a DPP4 inhibitor, effectively reduced terminal alveolar sac enlargement in a murine elastase-induced emphysema model and mitigated lung damage markers in a cigarette smoke-induced COPD mouse model.^399^ Although oral DPP4 inhibitors are highly effective in treating type 2 diabetes, their DPP4 inhibitory effect in the lungs is not sustained, particularly at clinical dosages. Continuous inhibition of DPP4 in the lungs is important for promoting the proliferation of AT2 cells. Therefore, to overcome the limitations of these drugs’ pulmonary exposure, NZ-97, a DPP4 inhibitor specifically targeted to the lungs, was developed. Compared with systemic oral treatments, NZ-97, delivered intratracheally, improved lesions in a LPS-induced lung injury model at lower doses and with less frequent administration. Moreover, higher doses of NZ-97 demonstrated good tolerability without causing significant side effects. Compared with other approved DPP4 inhibitors delivered directly to the lungs via intratracheal routes, NZ-97 has longer retention in the lungs, better distribution, and superior efficacy. Lastly, long-term treatment has not shown adverse changes in alveolar structure, increased inflammation, or cellular proliferation, further confirming the efficacy and safety of NZ-97.^399^ A recent nationwide cohort study showed that DPP4 inhibitor use was associated with a significantly lower risk of mortality, cardiovascular events, respiratory complications, and lung cancer in patients with COPD and type 2 diabetes.^407^ This underscores the role of DPP4 as a central regulator of AT2 expansion and highlights it as a promising therapeutic approach for broadly stimulating regenerative repair in COPD, particularly during the recovery and maintenance phases after epithelial injury associated with exacerbations.

##### d Prostaglandin E2

PGE2 is an important lipid signaling molecule that, beyond its well known roles in inflammation and pain, has increasingly been recognized as a key regulator of tissue repair and regeneration.^408^, ^409^, ^410^ A study investigating the migratory behavior of airway epithelial cells before and after undergoing EMT observed that PGE2 exhibits a dual role. Before EMT, PGE2 and its prostaglandin E2 receptor 2 (EP2) and EP4 receptor agonists promoted epithelial cell migration and wound closure. However, after EMT induced by TGF-*β*1 or a proinflammatory cytokine cocktail, PGE2 and its EP2/EP4 agonists significantly inhibited cell migration, indicating a shift in signaling from promotion to inhibition. These findings reveal the context-dependent regulatory role of PGE2 signaling during airway remodeling, suggesting that selective activation of EP2 and EP4 may help regulate aberrant mesenchymal cell migration and preserve epithelial barrier integrity in COPD.^275^ In a study investigating different lung injury models, including innate immune stimulation (LPS), allergic response (ovalbumin, and exposure to inhaled pollutants (cigarette smoke), mice lacking functional EP4 receptors (Ptger4^−/−^) exhibited heightened airway inflammation. Endogenous PGE2 actively suppresses inflammation through EP4 receptor activation. Further in vitro studies using murine and human monocytes/alveolar macrophages confirmed that PGE2 inhibits cytokine release from LPS-stimulated cells. Notably, this effect was specifically mediated by EP4 receptor activation, as it was replicated by an EP4 agonist but not by EP1 to EP3 agonists, and was abolished by an EP4 receptor antagonist.^411^ Further, EP4 agonists could provide bronchoprotection (similar to EP2 activation in mice) and exert anti-inflammatory effects in humans, making them promising candidates for future drug development.^411^^,^^412^ Based on a transcriptomics-guided drug target discovery strategy using gene signatures from patients with smoking-associated COPD and mice chronically exposed to cigarette smoke, we have previously identified PGE2 as a potential drug target in alveolar epithelial progenitors that support repair. Interestingly, the EP4 rather than EP2 receptor seemed to mediate this protective effect of PGE2. Notably, EP4 agonism has shown beneficial effects in counteracting impaired airway organoid formation caused by cigarette smoke exposure.^245^ Given these promising results, further exploration of EP4 receptors as a target to specifically address defective lung repair in COPD is warranted. Despite its regenerative potential, PGE2 contributes to a self-perpetuating cycle of cellular senescence and chronic inflammation in COPD fibroblasts. Elevated PGE2 levels in COPD fibroblast have been linked to increased senescence-associated inflammation, which exacerbates disease progression. Modulating the PGE2 signaling pathway could therefore represent a novel strategy to counteract senescence and chronic inflammation in COPD.^413^ Beyond inflammation and epithelial repair, PGE2 is also an important regulator of stem cell proliferation and differentiation. In models of LPS-induced acute lung injury, MSCs pretreated with PGE2 exhibited enhanced protective effects, leading to improved lung repair and regeneration. PGE2 plays a complex yet essential role in lung health by balancing inflammation regulation, epithelial protection, and stem cell-mediated repair. Targeting the PGE2 signaling pathway, particularly via EP4 receptor activation, may offer therapeutic benefits in both preventing exacerbation-associated damage and promoting epithelial regeneration in COPD.^414^

Table 1 summarizes emerging therapeutic strategies specifically targeting COPD exacerbations. Current interventions largely provide short-term symptomatic relief but have little impact on restoring epithelial integrity or supporting lung regeneration during these acute events. Emerging strategies targeting barrier function, inflammation, and regeneration show promise but remain early-stage. The therapeutic focus is shifting from controlling inflammation to promoting tissue repair and functional recovery.

**Table 1:** Potential future therapies for COPD exacerbations

Pharmacological Action	Drug	Study Type	Study Detail	Outcome	Reference
Strategies to Enhance Airway Epithelial Barrier Function					
PKD inhibitor	kb-NB142-70 Gö6976	In vitro	16HBE14o	- Enhances barrier integrity by upregulating CLDN1 and regulating apical junctions - Reduces epithelial permeability	Gan et al^295^
	Gö6976	In vitro	Poly (I:C)-exposed 16HBE14o	- Reduces apical junction breakdown - Reduces barrier dysfunction - Attenuates poly (I:C)-induced permeability of cell monolayers	Rezaee et al^297^
AKAP inhibitor	St-Ht31	In vitro	16HBE14o cells stimulated with 1% CSE	- Prevents CSE-induced disruption of functional epithelial barrier - Maintains epithelial barrier integrity via E-cadherin regulation	Oldenburger et al^70^
HDAC6 inhibitor	CAY10603	In vitro In vivo	-HBE cells stimulated with 6% CSE -Cigarette smoke-induced COPD in mice	- Inhibits cigarette smoke-induced small airway remodeling - Regulates barrier dysfunction - Reverses EMT via the TGF-*β*1/Smad2/3 pathway in HBE cells	Zhang et al^299^
HDAC3 inhibitor	RGFP966	In vitro In vivo	-LPS-treated AT2 cells -ALI model in AT2-specific HDAC3 knockout mice	- Alleviates LPS-induced epithelial injury - Reduces inflammation, apoptosis, and oxidative stress - Preserves mitochondrial quality control - Maintains epithelial barrier integrity - Regulates FOXO1–ROCK1 axis in AT2 cells	Li et al^300^
Mucolytic agent	NAC	In vivo	Bleomycin-induced lung injury in rats	- Improves lung lesions - Reduces excessive mucus secretion - Reduces lung TNF-*α* and MPO inflammation markers	Mata et al^304^
		In vivo	Cigarette smoke-induced COPD in rats	- Improves lung function - Inhibits mucus hypersecretion - Reduces MUC5AC expression - Mitigates airway inflammation - Inhibits EGFR/MAPK pathway - Mitigates inflammation	Xu et al^305^
		In vivo	PM_2.5_-induced lung injury in rats	- Inhibits MAPK activation - Reduces oxidative stress - Mitigates inflammation	Ping et al^306^
		In vitro	RSV-infected BEAS-2B	- Inhibits RSV infection - Enhances antioxidant defenses - Mitigates inflammation	Chi et al^307^
		In vitro	RSV-infected primary HBECs	- Inhibits ICAM1 expression and RSV infection - Restores ciliary activity and antioxidant capacity - Reduces mucin secretion	Mata et al^308^
		Clinical trial	Randomized, placebo-controlled trial in 523 patients with COPD	- Shows limited efficacy in preventing lung function decline - Fails to significantly reduce the risk of COPD exacerbations	Decramer et al^309^
		Clinical trial	Prospective, randomized, double-blind, placebo-controlled trial (ChiCTR-TRC-090004600) in patients aged 40–80 y with moderate-to-severe COPD	- Reduces COPD exacerbation rate - Delays recurrent exacerbations - Improves respiratory symptoms - Fails to improve FEV1 or overall lung function - Shows good tolerability - Exhibits side effects similar to placebo	Zheng et al^310^
		Clinical trial	Ongoing double-blind, randomized controlled trial (NCT05706402) in patients with acute exacerbations of COPD	- No results published	NCT05706402
Notch3 inhibitor	*γ*-Secretase inhibitors (LY450139)	In vitro In vivo	-Human nasal epithelial cell -Male and female Foxj1/EGFP mice	- Promotes multiciliated cell expansion - Alleviates IL-13–induced goblet cell hyperplasia - Targets aberrant Notch signaling - Improves epithelial structural and functional remodeling	Vladar et al^59^

Regulating Airway Epithelial Inflammation					
NLRP3 inhibitor	Andrographolide	In vitro In vivo	-THP-1-derived macrophages stimulated with LPS -Elastase/LPS-induced acute exacerbations of COPD model in mice	- Reduces neutrophils, macrophages, and lymphocytes in BALF - Mitigates lung injury - Reduces IL-1*β* levels in BALF and cell supernatant - Demonstrates cellular safety and nontoxicity	Yu et al^312^
	RO7486967 (Selnoflast)	Clinical trial	Phase IB, double-blind, randomized, placebo-controlled trial (BP43098) with parallel groups in patients with COPD	- The study was terminated early	
p38 MAPK inhibitors	SD-282	In vivo	Cigarette smoke-induced COPD model in mice	- Reduces neutrophils and macrophages in BALF - Prevents and reverses pulmonary inflammation - Eliminates edema and mucin, and reduces wall thickening - Reduces IL-6 mRNA expression in the lungs	Medicherla et al^319^
	Acumapimod (BCT-197)	Clinical trial	Phase II, double-blind, randomized, placebo-controlled dose-exploration study (NCT01332297) in patients with moderate or severe acute exacerbations of COPD	- Improves FEV1 in acute exacerbations of COPD - Demonstrates safety without hepatic or dermatologic adverse events	Strâmbu et al^324^
	AZD7624	Clinical trial	30 patients with COPD	- Fails to show any benefit in patients with COPD	Patel et al^326^
	CHF6297	In vitro In vivo	-LPS-induced PBMCs -TNF-*α* or CSE-induced BEAS-2B -Cigarette smoke-induced model in mice -IL-1*β*-induced neutrophilia model in rats	- Inhibits IL-8 and TNF-α release in vitro - Reduces neutrophils in BALF in mice and rats - Decreases neutrophils and IL-6 in BALF in rats	Martucci et al^327^
PDE 3/4 dual inhibitor	Ensifentrine	Clinical trial	Phase III, double-blind, randomized, placebo-controlled multicenter studies (NCT04535986 and NCT04542057) in patients aged 70–80 y with severe COPD	- Improves FEV1 - Improves symptoms - improves quality of life - Reduces the moderate or severe exacerbation rate - Shows good tolerability - Exhibits side effects similar to placebo (FDA-approved in 2024 for COPD)	Anzueto et al^336^
Anti–IL-4R*α* antibody	Dupilumab	Clinical trial	Phase III, double-blind, randomized, placebo-controlled BOREAS trial (NCT03930732) in patients with moderate-to-severe COPD with type 2 inflammation	- Decreases exacerbation frequency - Improves lung function - Improves quality of life - Reduces type 2 inflammation (FDA-approved in 2024 for COPD)	Bhatt et al^20^
Anti-TSLP antibody	Tezepelumab	Clinical trial	A multicenter, randomized, double-blind, placebo-controlled, parallel group, phase IIa study (NCT04039113)	- Fails to significantly reduce moderate/severe exacerbation rate - Improves prebronchodilator FEV1 - Shows good tolerability - Exhibits side effects similar to placebo	Singh et al^341^
		Clinical trial	Ongoing phase II, double-blind, randomized, placebo-controlled dose-exploration study (NCT05507242) in patients with COPD on LABA+LAMA±ICS who have experienced ≥1 exacerbation in the past 12mo	- No results published	NCT05507242
Anti-IL-33 antibody	Tozorakimab	Clinical trial	Phase I, double-blind, randomized, placebo-controlled dose-exploration study (NCT03096795) in participants with and without COPD	- Reduces serum IL-5 and IL-13 in patients with COPD - Shows good tolerability - Shows no treatment-associated serious adverse events	Reid et al^343^
		Clinical trial	A phase II, multicenter, double-blind, randomized, placebo-controlled study (NCT04631016) in patients with moderate to severe COPD and chronic bronchitis	- Improves lung function - Decreases exacerbation frequency	Pandya et al^344^
		Clinical trial	Ongoing phase III, double-blind, randomized, placebo-controlled study (NCT05158387 and NCT05166889) in patients with COPD with a history of COPD exacerbations	- No results published	NCT05158387NCT05166889
	Itepekimab	Clinical trial	Phase II, double-blind, randomized, placebo-controlled study (NCT03546907) in patients with moderate-to-severe COPD	- Reduces exacerbation rate and improves FEV1 in former smokers - Fails to reduce exacerbations in current smokers - Shows good tolerability - Exhibits side effects similar to placebo	Rabe et al^347^
		Clinical trial	Phase III, double-blind, randomized, placebo-controlled studies (NCT04701983) in patients with moderate-to-severeCOPD	- Shows a 27% reduction in annualized moderate/severe exacerbation rate at week 52	Kaltwasser et al^349^
		Clinical trial	Phase III, double-blind, randomized, placebo-controlled studies (NCT04751487) in patients with moderate-to-severeCOPD	- Fails to meet the primary endpoint, though early improvement observed at week 24	Kaltwasser et al^349^
Anti–IL-33R-antibody	Astegolimab	Clinical trial	Ongoing phase III open-label extension study (NCT05878769) in patients with COPD	- No results published	NCT05878769
		Clinical trial	Ongoing phase IIb, double-blind, randomized, placebo-controlled study (NCT05037929) in patients with COPD	- No results published	NCT05037929
		Clinical trial	Ongoing phase III, double-blind, randomized, placebo-controlled, multicenter study (NCT05595642) in patients with COPD	- No results published	NCT05595642

Boosting Lung Repair/Regeneration					
Stem cell therapy	Quercetin	In vitro Clinical trial	-ALI-cultured bronchial basal cells from patients with COPD -Patients with COPD aged 40–80 y (NCT03989271)	- Enhances epithelial polarization and barrier function - Reduces expression of inflammatory cytokines (eg, IL-8) - Modulates multiple genes related to epithelial repair, inflammation, and mucus secretion - Regulates gene expression in patients with COPD	McCluskey et al^358^
	P63^+^ lung progenitor cells	Clinical trial	Single-centered, nonrandomized, concurrent control study (NCT03188627) in patients with COPD	- Improves gas exchange function - Improves quality of life - Boosts exercise capacity - Shows good safety for patients with COPD	Wang et al^199^
	P63^+^ lung progenitor cells	Clinical trial	Phase I/II, randomized, single-blinded study (NCT03655808) for treatment of bronchiectasis	- Improves lung function - Reduces lung injury - Improves quality of life	Yan et al^359^
	BM-MSC	In vitro	Coculture of BM-MSCs with heat-shocked small airway epithelial cells	- Promotes differentiation of BM-MSCs into epithelial-like cells - Restores epithelial monolayer integrity - Expresses key small airway epithelial markers - Promotes tissue regeneration via direct differentiation and cell fusion - Promotes epithelial repair	Spees et al^363^
	BM-MSC	In vitro	ALI coculture of BM-MSC and NHBE cells	- Adheres to NHBE cells and undergo differentiation - Forms a composite tissue structure resembling airway epithelium - Supports epithelial regeneration on decellularized scaffolds	Le Visage et al^370^
	Prochymal(ex vivo cultured human adult mesenchymal stem cells)	Clinical trial	Phase II, double-blinded, randomized, placebo-controlled, multicenter study (NCT00683722) in patients with moderate-to-severe COPD with type 2 inflammation	- Reduces CRP levels early - Shows no significant differences in COPD exacerbation frequency - Exhibits good safety in patients with moderate-to-severe COPD	Weiss et al^371^
	BM-MSC	Clinical trial	Open-label, nonrandomized, nonblinded, prospective clinical trial (NCT01306513) for patients ≥40 y old with end-stage emphysema eligible for lung volume reduction surgery	- Increases CD31+ endothelial cells in alveolar septa - Avoids fibrosis and abnormal proliferation in lung tissue after MSC treatment - Demonstrates safety and feasibility - Causes no infusion-related adverse events	Stolk et al^372^
	BM-MSC	Clinical trial	A single-center, phase I clinical trial (ACTRN12614000731695) in patients with mild to very severe stable COPD	- Reduces hospital admissions for acute exacerbations of COPD - Decreases systemic inflammation - Shows no change in lung function - Shows good safety and tolerability	Armitage et al^373^
	EBV + MSC therapy	Clinical trial	Phase I, prospective, patient-blinded, randomized, placebo-controlled study (NCT01872624) in patients with severe COPD	- Improves quality of life - Reduces inflammation - Shows no significant between-group differences in COPD exacerbation frequency	de Oliveira et al^375^
	AD-MSCs	In vitro	Coculture of AD-MSCs with human small airway epithelial cells	- Enhances viability of small airway epithelial cells - Upregulates epithelial markers (MUC1, ICAM1) - Improves epithelial function - Promotes airway epithelial protection and regeneration	Schmelzer et al^380^
	AD-MSCs	Case report	A 57-y-old COPD man patient	- Improves dyspnea - Improves quality of life - Prevents COPD exacerbations over a 12-mo follow-up	Nguyen et al^381^
	AD-MSCs	Clinical trial	A phase III, double-blind, multicenter, randomized, placebo-controlled study in patients with moderate COPD	- No results published	Yancor et al^382^
	UC-MSCs	Clinical trial	A pilot clinical trial (ISRCTN70443938) in patients with COPD	- Improves dyspnea - Improves quality of life - Decreases exacerbation frequency - Demonstrates safety in patients with moderate-to-severe COPD	Le Thi Bich et al^385^
	UC-MSCs	Clinical trial	A phase I/II trial (NCT04433104) in patients with moderate-to-severe COPD	- No results published	Hoang et al^386^
	UC-MSCs	Clinical study	5 patients with COPD	- Improves lung function - Alleviates the severity of symptoms of COPD - Improves quality of life	Karaoz et al^387^
	UMC119-06-05	Clinical trial	Ongoing phase II, multicenter, double-blind, randomized, placebo-controlled study (NCT06491043) in patients with moderate-to-severe COPD	- No results published	NCT06491043
EV therapy	hUC-MSC-derived EVs	In vivo	Cigarette smoke-induced COPD model in rats	- Mitigates inflammation - Relieves septal thickening - Decreases goblet cell numbers - Reduces the levels of NF-κB subunit p65 in the tissue	Ridzuan et al^391^
	hBM-MSC-derived EVs	In vitro In vivo	- *Escherichia coli* endotoxin-induced acute lung injury in mice - Cytomix (IL-1*β*, TNF*α*, and IFN-*γ*)-induced primary cultures of human alveolar epithelial type II cells	- Reduces lung protein permeability and edema - Mitigates inflammation - Restores cytomix-induced protein permeability in vitro	Zhu et al^392^
	hBM-MSC-derived EVs	In vitro In vivo	- *E coli* pneumonia–induced acute lung injury in mice - Cytomix/LPS-induced alveolar epithelial type II cells	- Decreased lung inflammation, protein permeability, and histological severity in E coli Pneumonia - Improves survival from E coli pneumonia in mice - Reduces inflammation and increases ATP levels in alveolar epithelial type 2 cells	Monsel et al^393^
	mBM-MSC-derived Exos	In vitro In vivo Clinical study	- CSE-induced apoptosis in PMVECs - CSE-induced COPD model in mice - Lung tissue from patients with COPD	- Inhibits CSE-induced PMVEC apoptosis - Upregulates miR-30b and downregulates Wnt5a - Regulates apoptosis by lowering Bax and cleaved caspase-3 while increasing Bcl-2 - Improves emphysema (reduced MLI and DI) - Mimics exosomal effects with miR-30b; reverse them by overexpressing Wnt5a - Exhibits low miR-30b and high Wnt5a expression in patients with COPD	Song et al^394^
	Exo-d-MAPPS	In vivo Clinical study	- Cigarette smoke-induced COPD in mice - 30 patients with COPD	- Attenuates chronic airway inflammation in mice - Attenuates influx of inflammatory macrophages, neutrophils, and NK and NKT in inflamed lungs - Results in the elevation of anti-inflammatory and immunosuppressive IL-10 - Improves lung function - Improves quality of life - Mitigates inflammation - Modulates immune cell function - Enhances tissue repair and regeneration and improved pulmonary function of CS-exposed animals and patients with COPD	Harrell et al^395^
TR*β* agonist	Sobetirome	In vivo	- Bleomycin-induced pulmonary fibrosis in mice - LPS-induced lung injury in mice - Silica-induced lung injury model in mice	- Drives the AT1 cell differentiation - Promotes lung regeneration - Exerts antifibrotic properties	Pan et al^396^
DPP4 inhibitor	NZ-97	In vivo	- Bleomycin-induced lung injury in mice - LPS-induced lung injury in mice	- Improves lung injury - Promotes AT2 cell proliferation - Promotes lung repair - Improves lung fibrosis	Shao et al^399^
PGE2 (EP2, EP4 receptor agonists)	EP2 and EP4 agonists	In vitro	- HBECs - EMT induced by TGF-*β*1/inflammatory cytokine cocktail	Before EMT: - Promotes epithelial cell migration and wound closure After EMT: - Inhibits epithelial cell migration via EP2/EP4 - Regulates aberrant mesenchymal cell behavior - preserves epithelial barrier	Li et al^275^
	ONO-AE1-329	In vitro In vivo	- LPS-induced airway inflammation in mice (WT vs EP1–EP4 knockout mice) - Cigarette smoke-induced COPD model in mice (wild-type vs EP4 knockout (Ptger4−/−)) - LPS-induced murine and human monocyte/macrophage cell	- Shows increased neutrophil levels in EP4 knockout mice compared with WT in LPS-induced airway inflammation - Exhibits enhanced neutrophilic inflammation in EP4 knockout mice compared with WT in CS-induced COPD model - Decreases TNF-*α*, IL-6 protein production in monocytes	Birrell et al^411^
	ONO-AE1-329	Ex vivo	- Guinea pig, murine, monkey, rat, and human isolated airways	- Causes robust airway smooth muscle relaxation; effect blocked by EP4 antagonists in rat - Strong relaxation of airway smooth muscle; not replicated by EP2 agonists in human - Fails to induce significant relaxation; show EP2 dominance in guinea pig, monkey, and mouse	Buckley et al^412^
	CAY10598	In vitro In vivo	- CSE-exposed airway organoid culture - Cigarette smoke-induced COPD model in mice	- Increases number of alveolar-type organoids - Prevents CSE-induced reduction in organoid formation - Increases organoid size in both control and CSE conditions - Prevents disruption of circadian clock and cell cycle/apoptosis signaling - Promotes lung repair and regeneration	Wu et al^245^

Bax, Bcl-2-associated X protein; Bcl-2, B-cell Lymphoma 2; CRP, C-reactive protein; DI, destructive index; EBV, endobronchial valve; FDA, US Food and Drug Administration; FOXO1, Forkhead box O1; ICAM1, intercellular adhesion molecule 1; ICS, inhaled corticosteroid; LABA, long-acting *β*-agonist; LAMA, long-acting muscarinic antagonist; MAPK, mitogen-activated protein kinase; miR, microRNA; MLI, mean linear intercept; MPO, myeloperoxidase; MUC1, mucin 1; NF-*κ*B, nuclear factor-*κ*B; NK, natural killer; NKT, natural killer T cells; PBMC, peripheral blood mononuclear cell; PMVEC, pulmonary microvascular endothelial cells; ROCK1, Rho-associated protein kinase 1; RSV, respiratory syncytial virus; Smad2/3, mothers against decapentaplegic homolog 2/3; Wnt5a, wingless-type MMTV integration site family member 5A; WT, wild-type.

## VI Conclusion and future perspectives

COPD exacerbations are a major driver of disease progression, significantly contributing to morbidity, mortality, and healthcare burden. These episodes, often triggered by infections or environmental insults, lead to acute worsening of respiratory symptoms and lung function. At the center of this pathological cascade is the airway epithelium, which plays a pivotal role in maintaining barrier integrity, regulating immune responses, and initiating tissue repair. However, during exacerbations, epithelial function becomes severely compromised because of chronic inflammation, oxidative stress, and impaired regenerative capacity, resulting in sustained injury and abnormal remodeling.

Despite the critical role of the epithelium, current therapies for COPD exacerbations primarily aim to relieve symptoms and suppress inflammation, with minimal effect on epithelial health or repair. Bronchodilators, corticosteroids, and antibiotics remain the mainstay of treatment but do not restore epithelial function or prevent long-term tissue damage. This highlights a major therapeutic gap: how to preserve and restore epithelial integrity during and after exacerbations. Exacerbations should be viewed not only as episodes of acute damage but also as critical therapeutic windows, during which epithelial progenitor cells are activated and may be particularly responsive to repair-promoting interventions. If we can shift the balance from harmful inflammatory responses toward activation of endogenous repair processes, we may limit structural damage and support functional recovery. Targeting this window with well timed, combination therapies could help prevent maladaptive remodeling and ultimately alter the course of disease progression.

Increasing evidence supports a paradigm shift toward mechanism-based and regenerative strategies that go beyond inflammation control to promote epithelial repair and reinforce barrier function. Multimodal approaches, combining anti-inflammatory agents with compounds that enhance epithelial regeneration, represent a promising direction. Importantly, this may require re-evaluating therapies tested in stable COPD to determine whether they also support epithelial repair mechanisms during exacerbations. Moreover, treatments effective in stable state to prevent exacerbations need not be effective to treat the acute phase of exacerbations, and vice versa. Future efforts should optimize intervention timing and delivery, and shift the therapeutic focus from damage control to functional recovery.

Given the heterogeneity of exacerbation triggers, whether bacterial, viral, or environmental, personalized treatment approaches are essential. Tailoring therapies based on inflammatory endotypes and using biomarkers to guide intervention timing and selection could significantly enhance treatment efficacy and outcomes. However, to realize this potential, we must address the limitations of current preclinical models, which often fail to capture the chronic injury background, recurrent exacerbations, polymicrobial nature of triggers, and the dynamic multicellular interactions within the lung. Future research should prioritize more integrated and translational models, as well as clinical trials designed to evaluate combination therapies during and after exacerbations, with attention to patient stratification.

In summary, advancing our understanding of epithelial responses during COPD exacerbations, and translating this knowledge into targeted therapeutic interventions offers a promising path toward meaningful clinical impact. A shift from symptomatic treatment to mechanism-driven, regeneration-focused strategies may not only improve control of acute episodes but also preserve long-term lung function and improve outcomes for patients living with COPD.

## Conflict of interest

The authors declare no conflicts of interest.

## References

[1] Global strategy for prevention, diagnosis and management of COPD: 2025 report. Global Initiative for Chronic Obstructive Lung Disease. https://goldcopd.org/2025-gold-report/.

[2] Xu X., Huang K., Dong F. (2021). The heterogeneity of inflammatory response and emphysema in chronic obstructive pulmonary disease. Front Physiol.

[3] Milne K.M., Mitchell R.A., Ferguson O.N., Hind A.S., Guenette J.A. (2024). Sex-differences in COPD: from biological mechanisms to therapeutic considerations. Front Med.

[4] Salvi S. (2014). Tobacco smoking and environmental risk factors for chronic obstructive pulmonary disease. Clin Chest Med.

[5] Adeloye D., Song P., Zhu Y., Campbell H., Sheikh A., Rudan I., NIHR RESPIRE Global Respiratory Health Unit (2022). Global, regional, and national prevalence of, and risk factors for, chronic obstructive pulmonary disease (COPD) in 2019: a systematic review and modelling analysis. Lancet Respir Med.

[6] MacLeod M., Papi A., Contoli M. (2021). Chronic obstructive pulmonary disease exacerbation fundamentals: diagnosis, treatment, prevention and disease impact. Respirology.

[7] Gayle A.V., Quint J.K., Fuertes E.I. (2021). Understanding the relationships between environmental factors and exacerbations of COPD. Expert Rev Respir Med.

[8] Li J., Sun S., Tang R. (2016). Major air pollutants and risk of COPD exacerbations: a systematic review and meta-analysis. Int J Chron Obstruct Pulmon Dis.

[9] Wedzicha J.A., Seemungal T.A.R. (2007). COPD exacerbations: defining their cause and prevention. Lancet.

[10] Hillas G., Perlikos F., Tzanakis N. (2016). Acute exacerbation of COPD: is it the “stroke of the lungs”. Int J Chron Obstruct Pulmon Dis.

[11] Love M.E., Proud D. (2022). Respiratory viral and bacterial exacerbations of COPD—the role of the airway epithelium. Cells.

[12] Hiemstra P.S., McCray P.B., Bals R. (2015). The innate immune function of airway epithelial cells in inflammatory lung disease. Eur Respir J.

[13] Parker D., Prince A. (2011). Innate immunity in the respiratory epithelium. Am J Respir Cell Mol Biol.

[14] Bals R., Hiemstra P.S. (2004). Innate immunity in the lung: how epithelial cells fight against respiratory pathogens. Eur Respir J.

[15] Ganesan S., Sajjan U.S. (2013). Repair and remodeling of airway epithelium after injury in chronic obstructive pulmonary disease. Curr Respir Care Rep.

[16] Barnes P.J. (2016). Inflammatory mechanisms in patients with chronic obstructive pulmonary disease. J Allergy Clin Immunol.

[17] Li D., Kortekaas R.K., Douglas K.B.I. (2024). TNF signaling mediates lipopolysaccharide-induced lung epithelial progenitor cell responses in mouse lung organoids. Biomed Pharmacother.

[18] Croasdell Lucchini A., Gachanja N.N., Rossi A.G., Dorward D.A., Lucas C.D. (2021). Epithelial cells and inflammation in pulmonary wound repair. Cells.

[19] Singh D., Lea S., Mathioudakis A.G. (2021). Inhaled phosphodiesterase inhibitors for the treatment of chronic obstructive pulmonary disease. Drugs.

[20] Bhatt S.P., Rabe K.F., Hanania N.A. (2023). Dupilumab for COPD with type 2 inflammation indicated by eosinophil counts. N Engl J Med.

[21] Seemungal T.A., Donaldson G.C., Paul E.A., Bestall J.C., Jeffries D.J., Wedzicha J.A. (1998). Effect of exacerbation on quality of life in patients with chronic obstructive pulmonary disease. Am J Respir Crit Care Med.

[22] Anzueto A. (2010). Impact of exacerbations on COPD. Eur Respir Rev.

[23] Simpson M., Kapfumvuti R., Niranjan S. (2024). Exploring risk factors for all-cause hospital readmissions following chronic obstructive pulmonary disease exacerbation patients discharged on steroid tapers. J Thorac Dis.

[24] Spies R., Potter M., Hollamby R. (2023). Sputum color as a marker for bacteria in acute exacerbations of chronic obstructive pulmonary disease: a systematic review and meta-analysis. Ann Am Thorac Soc.

[25] Anthonisen N.R., Manfreda J., Warren C.P., Hershfield E.S., Harding G.K., Nelson N.A. (1987). Antibiotic therapy in exacerbations of chronic obstructive pulmonary disease. Ann Intern Med.

[26] Crisafulli E., Manco A., Ferrer M. (2020). Pneumonic versus nonpneumonic exacerbations of chronic obstructive pulmonary disease. Semin Respir Crit Care Med.

[27] Couturaud F., Bertoletti L., Pastre J. (2021). Prevalence of pulmonary embolism among patients with COPD hospitalized with acutely worsening respiratory symptoms. JAMA.

[28] Stolz D., Breidthardt T., Christ-Crain M. (2008). Use of B-type natriuretic peptide in the risk stratification of acute exacerbations of COPD. Chest.

[29] Halpin D.M.G., Birk R., Brealey N. (2018). Single-inhaler triple therapy in symptomatic COPD patients: FULFIL subgroup analyses. ERJ Open Res.

[30] Donaldson G.C., Law M., Kowlessar B. (2015). Impact of prolonged exacerbation recovery in chronic obstructive pulmonary disease. Am J Respir Crit Care Med.

[31] Hurst J.R., Donaldson G.C., Quint J.K., Goldring J.J.P., Baghai-Ravary R., Wedzicha J.A. (2009). Temporal clustering of exacerbations in chronic obstructive pulmonary disease. Am J Respir Crit Care Med.

[32] Tan K.S., Lim R.L., Liu J. (2020). Respiratory viral infections in exacerbation of chronic airway inflammatory diseases: novel mechanisms and insights from the upper airway epithelium. Front Cell Dev Biol.

[33] Hewitt R., Farne H., Ritchie A., Luke E., Johnston S.L., Mallia P. (2016). The role of viral infections in exacerbations of chronic obstructive pulmonary disease and asthma. Ther Adv Respir Dis.

[34] Zwaans W.A., Mallia P., Van Winden M.E., Rohde G.G. (2014). The relevance of respiratory viral infections in the exacerbations of chronic obstructive pulmonary disease—a systematic review. J Clin Virol.

[35] Zheng X.-Y., Xu Y.-J., Guan W.-J., Lin L.-F. (2018). Regional, age and respiratory-secretion-specific prevalence of respiratory viruses associated with asthma exacerbation: a literature review. Arch Virol.

[36] Jafarinejad H., Moghoofei M., Mostafaei S., Salimian J., Azimzadeh Jamalkandi S., Ahmadi A. (2017). Worldwide prevalence of viral infection in AECOPD patients: a meta-analysis. Microb Pathog.

[37] Ko F.W., Chan K.P., Hui D.S. (2016). Acute exacerbation of COPD. Respirology.

[38] Bardhan M., Sarkar M., Singh D., Negi R., Sharma S. (2020). Clinical and microbiological profile of patients with acute exacerbation of COPD. Natl J Med Res.

[39] Finney L.J., Ritchie A., Pollard E., Johnston S.L., Mallia P. (2014). Lower airway colonization and inflammatory response in COPD: a focus on *Haemophilus influenzae*. Int J Chronic Obstruct Pulm Dis.

[40] Papi A., Bellettato C.M., Braccioni F. (2006). Infections and airway inflammation in chronic obstructive pulmonary disease severe exacerbations. Am J Respir Crit Care Med.

[41] Wark P.A.B., Tooze M., Powell H., Parsons K. (2013). Viral and bacterial infection in acute asthma and chronic obstructive pulmonary disease increases the risk of readmission. Respirology.

[42] Javorac J., Jevtić M., Živanović D., Ilić M., Bijelović S., Dragić N. (2021). What are the effects of meteorological factors on exacerbations of chronic obstructive pulmonary disease?. Atmosphere.

[43] Barnes P.J., Burney P.G., Silverman E.K. (2015). Chronic obstructive pulmonary disease. Nat Rev Dis Primers.

[44] Crotty Alexander L.E.C., Shin S., Hwang J.H. (2015). Inflammatory diseases of the lung induced by conventional cigarette smoke: a review. Chest.

[45] Raby K.L., Michaeloudes C., Tonkin J., Chung K.F., Bhavsar P.K. (2023). Mechanisms of airway epithelial injury and abnormal repair in asthma and COPD. Front Immunol.

[46] Ganesan S., Comstock A.T., Sajjan U.S. (2013). Barrier function of airway tract epithelium. Tissue Barriers.

[47] Corcoran T.E. (2024). New path for understanding mucociliary clearance. Thorax.

[48] Abrami M., Biasin A., Tescione F. (2024). Mucus structure, viscoelastic properties, and composition in chronic respiratory diseases. Int J Mol Sci.

[49] Nazir A., Clementius B. (2025). Airway mucus hypersecretion in chronic obstructive pulmonary disease patients: from basic pathophysiology to rehabilitation approaches. Surabaya Phys Med Rehabilitation J.

[50] Han S., Mallampalli R.K. (2015). The role of surfactant in lung disease and host defense against pulmonary infections. Ann Am Thorac Soc.

[51] Wang X., Hao Y., Yin Y. (2024). Lianhua Qingke preserves mucociliary clearance in rat with acute exacerbation of chronic obstructive pulmonary disease by maintaining ciliated cells proportion and protecting structural integrity and beat function of cilia. Int J Chron Obstruct Pulmon Dis.

[52] Bowler R.P. (2012). Surfactant protein D as a biomarker for chronic obstructive pulmonary disease. COPD.

[53] Singanayagam A., Footitt J., Marczynski M. (2022). Airway mucins promote immunopathology in virus-exacerbated chronic obstructive pulmonary disease. J Clin Invest.

[54] Chillappagari S., Preuss J., Licht S. (2015). Altered protease and antiprotease balance during a COPD exacerbation contributes to mucus obstruction. Respir Res.

[55] Meldrum O.W., Chotirmall S.H. (2021). Mucus, microbiomes and pulmonary disease. Biomedicines.

[56] Simet S.M., Sisson J.H., Pavlik J.A. (2010). Long-term cigarette smoke exposure in a mouse model of ciliated epithelial cell function. Am J Respir Cell Mol Biol.

[57] Rock J.R., Gao X., Xue Y., Randell S.H., Kong Y.Y., Hogan B.L.M. (2011). Notch-dependent differentiation of adult airway basal stem cells. Cell Stem Cell.

[58] Tsao P.N., Vasconcelos M., Izvolsky K.I., Qian J., Lu J., Cardoso W.V. (2009). Notch signaling controls the balance of ciliated and secretory cell fates in developing airways. Development.

[59] Vladar E.K., Kunimoto K., Rojas-Hernandez L.S. (2023). Notch signaling inactivation by small molecule γ-secretase inhibitors restores the multiciliated cell population in the airway epithelium. Am J Physiol Lung Cell Mol Physiol.

[60] Bodas M., Moore A.R., Subramaniyan B. (2021). Cigarette smoke activates notch3 to promote goblet cell differentiation in human airway epithelial cells. Am J Respir Cell Mol Biol.

[61] Zong D., Ouyang R., Li J., Chen Y., Chen P. (2016). Notch signaling in lung diseases: focus on Notch1 and notch3. Ther Adv Respir Dis.

[62] Jing Y., Gimenes J.A., Mishra R. (2019). Notch3 contributes to rhinovirus-induced goblet cell hyperplasia in COPD airway epithelial cells. Thorax.

[63] Gao N., Rezaee F. (2022). Airway epithelial cell junctions as targets for pathogens and antimicrobial therapy. Pharmaceutics.

[64] Hou W., Hu S., Li C. (2019). Cigarette smoke induced lung barrier dysfunction, EMT, and tissue remodeling: a possible link between COPD and lung cancer. BioMed Res Int.

[65] Carlier F.M., Detry B., Lecocq M. (2024). The memory of airway epithelium damage in smokers and COPD patients. Life Sci Alliance.

[66] Ye R., Wang C., Sun P., Bai S., Zhao L. (2021). AGR3 regulates airway epithelial junctions in patients with frequent exacerbations of COPD. Front Pharmacol.

[67] Nishida K., Brune K.A., Putcha N. (2017). Cigarette smoke disrupts monolayer integrity by altering epithelial cell-cell adhesion and cortical tension. Am J Physiol Lung Cell Mol Physiol.

[68] Heijink I.H., Noordhoek J.A., Timens W., van Oosterhout A.J.M., Postma D.S. (2014). Abnormalities in airway epithelial junction formation in chronic obstructive pulmonary disease. Am J Respir Crit Care Med.

[69] Aghapour M., Raee P., Moghaddam S.J., Hiemstra P.S., Heijink I.H. (2018). Airway epithelial barrier dysfunction in chronic obstructive pulmonary disease: role of cigarette smoke exposure. Am J Respir Cell Mol Biol.

[70] Oldenburger A., Poppinga W.J., Kos F. (2014). A-kinase anchoring proteins contribute to loss of E-cadherin and bronchial epithelial barrier by cigarette smoke. Am J Physiol Cell Physiol.

[71] Kim B.G., Lee P.H., Lee S.H. (2018). Impact of the endothelial tight junction protein Claudin-5 on clinical profiles of patients with COPD. Allergy Asthma Immunol Res.

[72] Park S., Lee P.H., Baek A.R. (2021). Association of the tight junction protein Claudin-4 with lung function and exacerbations in chronic obstructive pulmonary disease. Int J Chron Obstruct Pulmon Dis.

[73] Bals R., Hiemstra P.S. (2006). Antimicrobial peptides in COPD—basic biology and therapeutic applications. Curr Drug Targets.

[74] Lecaille F., Lalmanach G., Andrault P.M. (2016). Antimicrobial proteins and peptides in human lung diseases: a friend and foe partnership with host proteases. Biochimie.

[75] Beasley V., Joshi P.V., Singanayagam A., Molyneaux P.L., Johnston S.L., Mallia P. (2012). Lung microbiology and exacerbations in COPD. Int J Chron Obstruct Pulmon Dis.

[76] Mallia P., Footitt J., Sotero R. (2012). Rhinovirus infection induces degradation of antimicrobial peptides and secondary bacterial infection in chronic obstructive pulmonary disease. Am J Respir Crit Care Med.

[77] Parameswaran G.I., Wrona C.T., Murphy T.F., Sethi S. (2009). *Moraxella catarrhalis* acquisition, airway inflammation and protease-antiprotease balance in chronic obstructive pulmonary disease. BMC Infect Dis.

[78] Persson L.J.P., Aanerud M., Hardie J.A. (2017). Antimicrobial peptide levels are linked to airway inflammation, bacterial colonisation and exacerbations in chronic obstructive pulmonary disease. Eur Respir J.

[79] Arnason J.W., Murphy J.C., Kooi C. (2017). Human *β*-defensin-2 production upon viral and bacterial co-infection is attenuated in COPD. PLoS One.

[80] Nida, Lone K.P. (2018). Plasma surfactant protein-A levels in apparently healthy smokers, stable and exacerbation COPD patients. Pak J Med Sci.

[81] Shakoori T.A., Sin D.D., Ghafoor F., Bashir S., Bokhari S.N.H. (2009). Serum surfactant protein D during acute exacerbations of chronic obstructive pulmonary disease. Dis Markers.

[82] Tkacova R., McWilliams A., Lam S., Sin D.D. (2010). Integrating lung and plasma expression of pneumo-proteins in developing biomarkers in COPD: a case study of surfactant protein D. Med Sci Monit.

[83] Winkler C., Atochina-Vasserman E.N., Holz O. (2011). Comprehensive characterisation of pulmonary and serum surfactant protein D in COPD. Respir Res.

[84] Sin D.D., Pahlavan P.S., Man S.F.P. (2008). Surfactant protein D: a lung specific biomarker in COPD?. Ther Adv Respir Dis.

[85] Parameswaran G.I., Sethi S., Murphy T.F. (2011). Effects of bacterial infection on airway antimicrobial peptides and proteins in COPD. Chest.

[86] Brusselle G.G., Joos G.F., Bracke K.R. (2011). New insights into the immunology of chronic obstructive pulmonary disease. Lancet.

[87] Hartl D., Tirouvanziam R., Laval J. (2018). Innate immunity of the lung: from basic mechanisms to translational medicine. J Innate Immun.

[88] Pizzichini E., Pizzichini M.M., Gibson P. (1998). Sputum eosinophilia predicts benefit from prednisone in smokers with chronic obstructive bronchitis. Am J Respir Crit Care Med.

[89] Saha S., Brightling C.E. (2006). Eosinophilic airway inflammation in COPD. Int J Chron Obstruct Pulmon Dis.

[90] Leigh R., Pizzichini M.M., Morris M.M., Maltais F., Hargreave F.E., Pizzichini E. (2006). Stable COPD: predicting benefit from high-dose inhaled corticosteroid treatment. Eur Respir J.

[91] Singh D., Kolsum U., Brightling C.E., Locantore N., Agusti A., Tal-Singer R., ECLIPSE investigators (2014). Eosinophilic inflammation in COPD: prevalence and clinical characteristics. Eur Respir J.

[92] Oishi K., Matsunaga K., Shirai T., Hirai K., Gon Y. (2020). Role of Type2 inflammatory biomarkers in chronic obstructive pulmonary disease. J Clin Med.

[93] Le J., Kulatheepan Y., Jeyaseelan S. (2023). Role of toll-like receptors and nod-like receptors in acute lung infection. Front Immunol.

[94] Guo-Parke H., Linden D., Weldon S., Kidney J.C., Taggart C.C. (2022). Deciphering respiratory-virus-associated interferon signaling in COPD airway epithelium. Medicina (Kaunas).

[95] Pelaia C., Vatrella A., Gallelli L. (2021). Role of p38 mitogen-activated protein kinase in asthma and COPD: pathogenic aspects and potential targeted therapies. Drug Des Devel Ther.

[96] Qi Y.J., Sun X.J., Wang Z. (2020). Richness of sputum microbiome in acute exacerbations of eosinophilic chronic obstructive pulmonary disease. Chin Med J (Engl).

[97] Guo-Parke H., Linden D., Weldon S., Kidney J.C., Taggart C.C. (2020). Mechanisms of virus-induced airway immunity dysfunction in the pathogenesis of COPD disease, progression, and exacerbation. Front Immunol.

[98] Nadigel J., Audusseau S., Baglole C.J., Eidelman D.H., Hamid Q. (2013). IL-8 production in response to cigarette smoke is decreased in epithelial cells from COPD patients. Pulm Pharmacol Ther.

[99] Dong Y., Dong Y., Zhu C. (2024). Targeting CCL2-CCR2 signaling pathway alleviates macrophage dysfunction in COPD via PI3K-AKT axis. Cell Commun Signal.

[100] Christopoulou M.E., Papakonstantinou E., Stolz D. (2023). Matrix metalloproteinases in chronic obstructive pulmonary disease. Int J Mol Sci.

[101] Boukhenouna S., Wilson M.A., Bahmed K., Kosmider B. (2018). Reactive oxygen species in chronic obstructive pulmonary disease. Oxid Med Cell Longev.

[102] Mercer P.F., Shute J.K., Bhowmik A., Donaldson G.C., Wedzicha J.A., Warner J.A. (2005). MMP-9, TIMP-1 and inflammatory cells in sputum from COPD patients during exacerbation. Respir Res.

[103] Vaitkus M., Lavinskiene S., Barkauskiene D., Bieksiene K., Jeroch J., Sakalauskas R. (2013). Reactive oxygen species in peripheral blood and sputum neutrophils during bacterial and nonbacterial acute exacerbation of chronic obstructive pulmonary disease. Inflammation.

[104] Pettersen C.A., Adler K.B. (2002). Airways inflammation and COPD: epithelial-neutrophil interactions. Chest.

[105] Russell R.J., Boulet L.P., Brightling C.E. (2024). The airway epithelium: an orchestrator of inflammation, a key structural barrier and a therapeutic target in severe asthma. Eur Respir J.

[106] Suri C., Pande B., Sahithi L.S., Sahu T., Verma H.K. (2024). Interplay between lung diseases and viral infections: a comprehensive review. Microorganisms.

[107] Lugade A.A., Bogner P.N., Thatcher T.H., Sime P.J., Phipps R.P., Thanavala Y. (2014). Cigarette smoke exposure exacerbates lung inflammation and compromises immunity to bacterial infection. J Immunol.

[108] Mallia P., Message S.D., Gielen V. (2011). Experimental rhinovirus infection as a human model of chronic obstructive pulmonary disease exacerbation. Am J Respir Crit Care Med.

[109] Singanayagam A., Loo S.L., Calderazzo M. (2019). Antiviral immunity is impaired in COPD patients with frequent exacerbations. Am J Physiol Lung Cell Mol Physiol.

[110] Wu W., Patel K.B., Booth J.L., Zhang W., Metcalf J.P. (2011). Cigarette smoke extract suppresses the RIG-I-initiated innate immune response to influenza virus in the human lung. Am J Physiol Lung Cell Mol Physiol.

[111] Gaajetaan G.R., Geelen T.H., Vernooy J.H. (2013). Interferon-*β* induces a long-lasting antiviral state in human respiratory epithelial cells. J Infect.

[112] Baines K.J., Hsu A.C.Y., Tooze M., Gunawardhana L.P., Gibson P.G., Wark P.A.B. (2013). Novel immune genes associated with excessive inflammatory and antiviral responses to rhinovirus in COPD. Respir Res.

[113] Pianigiani T., Paggi I., Cooper G.E., Staples K.J., McDonnell M., Bergantini L. (2025). Natural killer cells in the lung: novel insight and future challenge in the airway diseases. ERJ Open Res.

[114] Chen J., Wang X., Schmalen A. (2023). Antiviral CD8+ T-cell immune responses are impaired by cigarette smoke and in COPD. Eur Respir J.

[115] Allie S.R., Randall T.D. (2017). Pulmonary immunity to viruses. Clin Sci.

[116] Schneider D., Ganesan S., Comstock A.T. (2010). Increased cytokine response of rhinovirus-infected airway epithelial cells in chronic obstructive pulmonary disease. Am J Respir Crit Care Med.

[117] Vareille M., Kieninger E., Edwards M.R., Regamey N. (2011). The airway epithelium: soldier in the fight against respiratory viruses. Clin Microbiol Rev.

[118] Vlahos R., Bozinovski S., Hamilton J.A., Anderson G.P. (2006). Therapeutic potential of treating chronic obstructive pulmonary disease (COPD) by neutralising granulocyte macrophage-colony stimulating factor (GM-CSF). Pharmacol Ther.

[119] Chakrabarti A., Mar J.S., Choy D.F. (2021). High serum granulocyte-colony stimulating factor characterises neutrophilic COPD exacerbations associated with dysbiosis. ERJ Open Res.

[120] Faner R., Sobradillo P., Noguera A. (2016). The inflammasome pathway in stable COPD and acute exacerbations. ERJ Open Res.

[121] Ji S., Dai M.Y., Huang Y. (2022). Influenza A virus triggers acute exacerbation of chronic obstructive pulmonary disease by increasing proinflammatory cytokines secretion via NLRP3 inflammasome activation. J Inflamm.

[122] Mallia P., Webber J., Gill S.K. (2018). Role of airway glucose in bacterial infections in patients with chronic obstructive pulmonary disease. J Allergy Clin Immunol.

[123] Dickson R.P., Erb-Downward J.R., Martinez F.J., Huffnagle G.B. (2016). The microbiome and the respiratory tract. Annu Rev Physiol.

[124] Sethi S., Murphy T.F. (2008). Infection in the pathogenesis and course of chronic obstructive pulmonary disease. N Engl J Med.

[125] Bello-Perez M., García-Pachón E., Gonzalo-Jimenez N. (2025). Gene expression profiles reveal distinct mechanisms driving chronic obstructive pulmonary disease exacerbations. Int J Mol Sci.

[126] Barnes P.J. (2018). Targeting cytokines to treat asthma and chronic obstructive pulmonary disease. Nat Rev Immunol.

[127] Yousuf A., Ibrahim W., Greening N.J., Brightling C.E. (2019). T2 biologics for chronic obstructive pulmonary disease. J Allergy Clin Immunol Pract.

[128] David B., Bafadhel M., Koenderman L., De Soyza A. (2021). Eosinophilic inflammation in COPD: from an inflammatory marker to a treatable trait. Thorax.

[129] Singh D., Agusti A., Martinez F.J. (2022). Blood eosinophils and chronic obstructive pulmonary disease: a Global Initiative for Chronic Obstructive Lung Disease science committee 2022 review. Am J Respir Crit Care Med.

[130] Rossi G.A., Ballarini S., Salvati P., Sacco O., Colin A.A. (2022). Alarmins and innate lymphoid cells 2 activation: a common pathogenetic link connecting respiratory syncytial virus bronchiolitis and later wheezing/asthma?. Pediatr Allergy Immunol.

[131] Gandhi N.A., Bennett B.L., Graham N.M.H., Pirozzi G., Stahl N., Yancopoulos G.D. (2016). Targeting key proximal drivers of type 2 inflammation in disease. Nat Rev Drug Discov.

[132] Singh D., Bafadhel M., Brightling C.E. (2020). Blood eosinophil counts in clinical trials for chronic obstructive pulmonary disease. Am J Respir Crit Care Med.

[133] Tsoumakidou M., Tzanakis N., Chrysofakis G., Kyriakou D., Siafakas N.M. (2005). Changes in sputum T-lymphocyte subpopulations at the onset of severe exacerbations of chronic obstructive pulmonary disease. Respir Med.

[134] Ghebre M.A., Pang P.H., Diver S. (2018). Biological exacerbation clusters demonstrate asthma and chronic obstructive pulmonary disease overlap with distinct mediator and microbiome profiles. J Allergy Clin Immunol.

[135] Christenson S.A., Steiling K., van den Berge M. (2015). Asthma-COPD overlap. Clinical relevance of genomic signatures of type 2 inflammation in chronic obstructive pulmonary disease. Am J Respir Crit Care Med.

[136] George L., Taylor A.R., Esteve-Codina A. (2020). Blood eosinophil count and airway epithelial transcriptome relationships in COPD versus asthma. Allergy.

[137] Contoli M., Ito K., Padovani A. (2015). Th2 cytokines impair innate immune responses to rhinovirus in respiratory epithelial cells. Allergy.

[138] Bagnasco D., Ferrando M., Varricchi G., Passalacqua G., Canonica G.W. (2016). A critical evaluation of anti-IL-13 and anti-IL-4 strategies in severe asthma. Int Arch Allergy Immunol.

[139] Allen J.E. (2023). IL-4 and IL-13: regulators and effectors of wound repair. Annu Rev Immunol.

[140] Varricchi G., Poto R. (2024). Towards precision medicine in COPD: targeting type 2 cytokines and alarmins. Eur J Intern Med.

[141] Kearley J., Silver J.S., Sanden C. (2015). Cigarette smoke silences innate lymphoid cell function and facilitates an exacerbated type I interleukin-33-dependent response to infection. Immunity.

[142] Joo H., Park S.J., Min K.H., Rhee C.K. (2021). Association between plasma interleukin-33 level and acute exacerbation of chronic obstructive pulmonary disease. BMC Pulm Med.

[143] Rhee C.K., Min K.H., Yoo K.H.Y., Jung K.-S. (2020). High interleukin-33 is associated with acute exacerbation in patients with chronic obstructive pulmonary disease. Eur Respir J.

[144] Calvén J., Yudina Y., Hallgren O. (2012). Viral stimuli trigger exaggerated thymic stromal lymphopoietin expression by chronic obstructive pulmonary disease epithelium: role of endosomal TLR3 and cytosolic RIG-I-like helicases. J Innate Immun.

[145] Nedeva D., Kowal K., Mihaicuta S. (2023). Epithelial alarmins: a new target to treat chronic respiratory diseases. Expert Rev Respir Med.

[146] Yoshida M., Arzili R., Nikolić M.Z. (2025). Immune-epithelial cell interactions in lung development, homeostasis and disease. Int J Biochem Cell Biol.

[147] Zhang J., Bai C. (2018). The significance of serum interleukin-8 in acute exacerbations of chronic obstructive pulmonary disease. Tanaffos.

[148] Grabcanovic-Musija F., Obermayer A., Stoiber W. (2015). Neutrophil extracellular trap (NET) formation characterises stable and exacerbated COPD and correlates with airflow limitation. Respir Res.

[149] Dicker A.J., Crichton M.L., Pumphrey E.G. (2018). Neutrophil extracellular traps are associated with disease severity and microbiota diversity in patients with chronic obstructive pulmonary disease. J Allergy Clin Immunol.

[150] Pedersen F., Marwitz S., Holz O. (2015). Neutrophil extracellular trap formation and extracellular DNA in sputum of stable COPD patients. Respir Med.

[151] Lonergan M., Dicker A.J., Crichton M.L. (2020). Blood neutrophil counts are associated with exacerbation frequency and mortality in COPD. Respir Res.

[152] Rizo-Téllez S.A., Filep J.G. (2024). Beyond host defense and tissue injury: the emerging role of neutrophils in tissue repair. Am J Physiol Cell Physiol.

[153] Pouwels S.D., van Geffen W.H., Jonker M.R., Kerstjens H.A.M., Nawijn M.C., Heijink I.H. (2017). Increased neutrophil expression of pattern recognition receptors during COPD exacerbations. Respirology.

[154] Zhou K., Wen Q., Zuo Y., Bai G., Sun R. (2025). Pathogenic cell in COPD: mechanisms of airway remodeling, immune dysregulation, and therapeutic implications. Int J Chron Obstruct Pulmon Dis.

[155] Hou F., Xiao K., Tang L., Xie L. (2021). Diversity of macrophages in lung homeostasis and diseases. Front Immunol.

[156] Chung K.F., Adcock I.M. (2008). Multifaceted mechanisms in COPD: inflammation, immunity, and tissue repair and destruction. Eur Respir J.

[157] Puttur F., Gregory L.G., Lloyd C.M. (2019). Airway macrophages as the guardians of tissue repair in the lung. Immunol Cell Biol.

[158] Feng H., Zheng R. (2023). Cigarette smoke prevents M1 polarization of alveolar macrophages by suppressing NLRP3. Life Sci.

[159] Xu M.M., Kang J.Y., Wang Q.Y. (2024). Melatonin improves influenza virus infection-induced acute exacerbation of COPD by suppressing macrophage M1 polarization and apoptosis. Respir Res.

[160] Chen Y., Li F., Hua M., Liang M., Song C. (2023). Role of GM-CSF in lung balance and disease. Front Immunol.

[161] Barnes P.J. (2014). Cellular and molecular mechanisms of chronic obstructive pulmonary disease. Clin Chest Med.

[162] Barnes P.J. (2004). Alveolar macrophages as orchestrators of COPD. COPD.

[163] Churg A., Zhou S., Wright J.L. (2011). Matrix metalloproteinases in COPD. Eur Respir J.

[164] Bazzan E., Turato G., Tinè M. (2017). Dual polarization of human alveolar macrophages progressively increases with smoking and COPD severity. Respir Res.

[165] He S., Tian R., Zhang X. (2023). PPARγ inhibits small airway remodeling through mediating the polarization homeostasis of alveolar macrophages in COPD. Clin Immunol.

[166] Eapen M.S., Hansbro P.M., McAlinden K. (2017). Abnormal M1/M2 macrophage phenotype profiles in the small airway wall and lumen in smokers and chronic obstructive pulmonary disease (COPD). Sci Rep.

[167] Gevaert P., Wong K., Millette L.A., Carr T.F. (2022). The role of IgE in upper and lower airway disease: more than just allergy. Clin Rev Allergy Immunol.

[168] McClure R., Massari P. (2014). TLR-dependent human mucosal epithelial cell responses to microbial pathogens. Front Immunol.

[169] Malik A., Batra J.K. (2012). Antimicrobial activity of human eosinophil granule proteins: involvement in host defence against pathogens. Crit Rev Microbiol.

[170] Lee Y.L., Heriyanto D.S., Yuliani F.S. (2024). Eosinophilic inflammation: a key player in COPD pathogenesis and progression. Ann Med.

[171] Bel E.H., Ten Brinke A. (2017). New anti-eosinophil drugs for asthma and COPD: targeting the trait. Chest.

[172] Bafadhel M., McKenna S., Terry S. (2011). Acute exacerbations of chronic obstructive pulmonary disease: identification of biologic clusters and their biomarkers. Am J Respir Crit Care Med.

[173] Vedel-Krogh S., Nielsen S.F., Lange P., Vestbo J., Nordestgaard B.G. (2016). Blood eosinophils and exacerbations in chronic obstructive pulmonary disease. The Copenhagen general population study. Am J Respir Crit Care Med.

[174] Price D., Rigazio A., Postma D. (2014). Blood eosinophilia and the number of exacerbations in COPD patients. Eur Respir J.

[175] Bathoorn E., Liesker J.J.W., Postma D.S. (2009). Change in inflammation in out-patient COPD patients from stable phase to a subsequent exacerbation. Int J Chron Obstruct Pulmon Dis.

[176] Bafadhel M., McKenna S., Terry S. (2012). Blood eosinophils to direct corticosteroid treatment of exacerbations of chronic obstructive pulmonary disease: a randomized placebo-controlled trial. Am J Respir Crit Care Med.

[177] George L., Brightling C.E. (2016). Eosinophilic airway inflammation: role in asthma and chronic obstructive pulmonary disease. Ther Adv Chronic Dis.

[178] Agustí A., Celli B.R., Criner G.J. (2023). Global Initiative for Chronic Obstructive Lung Disease 2023 report: GOLD executive summary. Eur Respir J.

[179] Romero-Linares A., Álvarez-Muro L., Hammadi A. (2025). Short term exacerbation risk and exhaled nitric oxide in COPD. Respir Med.

[180] Antus B., Barta I. (2022). Blood eosinophils and exhaled nitric oxide: surrogate biomarkers of airway eosinophilia in stable COPD and exacerbation. Biomedicines.

[181] Xu X., Zhou L., Tong Z. (2023). The relationship of fractional exhaled nitric oxide in patients with AECOPD. Int J Chron Obstruct Pulmon Dis.

[182] Kotton D.N., Morrisey E.E. (2014). Lung regeneration: mechanisms, applications and emerging stem cell populations. Nat Med.

[183] Crosby L.M., Waters C.M. (2010). Epithelial repair mechanisms in the lung. Am J Physiol Lung Cell Mol Physiol.

[184] Akram K.M., Patel N., Spiteri M.A., Forsyth N.R. (2016). Lung regeneration: endogenous and exogenous stem cell mediated therapeutic approaches. Int J Mol Sci.

[185] Wang C., Cassandras M., Peng T. (2019). The role of hedgehog signaling in adult lung regeneration and maintenance. J Dev Biol.

[186] Farooq M., Khan A.W., Kim M.S., Choi S. (2021). The role of fibroblast growth factor (FGF) signaling in tissue repair and regeneration. Cells.

[187] Varet J., Douglas S.K., Gilmartin L. (2010). VEGF in the lung: a role for novel isoforms. Am J Physiol Lung Cell Mol Physiol.

[188] Kortekaas R.K., Geillinger-Kästle K.E., Fuentes-Mateos R. (2024). The disruptive effects of COPD exacerbation-associated factors on epithelial repair responses. Front Immunol.

[189] Kapellos T.S., Conlon T.M., Yildirim A.Ö., Lehmann M. (2023). The impact of the immune system on lung injury and regeneration in COPD. Eur Respir J.

[190] Chen Y., Li Z., Ji G., Wang S., Mo C., Ding B.S. (2024). Lung regeneration: diverse cell types and the therapeutic potential. MedComm (2020).

[191] Gong Z., Li Q., Shi J. (2022). Lung fibroblasts facilitate pre-metastatic niche formation by remodeling the local immune microenvironment. Immunity.

[192] Zheng D., Soh B.-S., Yin L. (2017). Differentiation of club cells to alveolar epithelial cells in vitro. Sci Rep.

[193] Barnes P.J. (2015). Club cells, their secretory protein, and COPD. Chest.

[194] Hu X., Xu J., Li P., Zheng H. (2023). Correlation of serum Clara cell secretory Protein 16, plasma fibrinogen and serum amyloid A with the severity of acute exacerbated COPD and their combination in prognosis assessment. Int J Chron Obstruct Pulmon Dis.

[195] Braido F., Riccio A.M., Guerra L. (2007). Clara cell 16 protein in COPD sputum: a marker of small airways damage?. Respir Med.

[196] Sohal S.S. (2018). Airway basal cell reprogramming and epithelial-mesenchymal transition: a potential key to understanding early chronic obstructive pulmonary disease. Am J Respir Crit Care Med.

[197] Shaykhiev R. (2021). Airway basal cells in chronic obstructive pulmonary disease: a continuum or a dead end?. Am J Respir Cell Mol Biol.

[198] Lv Z., Liu Z., Liu K. (2024). Alveolar regeneration by airway secretory-cell-derived p63+ progenitors. Cell Stem Cell.

[199] Wang Y., Meng Z., Liu M. (2024). Autologous transplantation of P63+ lung progenitor cells for chronic obstructive pulmonary disease therapy. Sci Transl Med.

[200] Wijk S.C., Prabhala P., Michaliková B. (2021). Human primary airway basal cells display a continuum of molecular phases from health to disease in chronic obstructive pulmonary disease. Am J Respir Cell Mol Biol.

[201] Zuo W., Zhang T., Wu D.Z. (2015). p63(+)Krt5(+) distal airway stem cells are essential for lung regeneration. Nature.

[202] Fernanda de Mello Costa M., Weiner A.I., Vaughan A.E. (2020). Basal-like progenitor cells: a review of dysplastic alveolar regeneration and remodeling in lung repair. Stem Cell Rep.

[203] Olajuyin A.M., Zhang X., Ji H.L. (2019). Alveolar type 2 progenitor cells for lung injury repair. Cell Death Discov.

[204] Zhang J., Liu Y. (2024). Epithelial stem cells and niches in lung alveolar regeneration and diseases. Chin Med J Pulm Crit Care Med.

[205] Meng X., Cui G., Peng G. (2023). Lung development and regeneration: newly defined cell types and progenitor status. Cell Regen.

[206] Cheng Z., Bartel S., Nijnatten J.L.V. (2022). Transcriptome analysis indicates more AT2-to-AT1 transition in COPD. Eur Respir J.

[207] Yu H., Lin Y., Zhong Y. (2022). Impaired AT2 to AT1 cell transition in PM2.5-induced mouse model of chronic obstructive pulmonary disease. Respir Res.

[208] Darby I.A., Laverdet B., Bonté F., Desmoulière A. (2014). Fibroblasts and myofibroblasts in wound healing. Clin Cosmet Investig Dermatol.

[209] Grigorieva O., Basalova N., Vigovskiy M. (2023). Novel potential markers of myofibroblast differentiation revealed by single-cell RNA sequencing analysis of mesenchymal stromal cells in profibrotic and adipogenic conditions. Biomedicines.

[210] Dupin I., Contin-Bordes C., Berger P. (2018). Fibrocytes in asthma and chronic obstructive pulmonary disease: variations on the same theme. Am J Respir Cell Mol Biol.

[211] Reilkoff R.A., Bucala R., Herzog E.L. (2011). Fibrocytes: emerging effector cells in chronic inflammation. Nat Rev Immunol.

[212] van Dijk E.M., Menzen M.H., Spanjer A.I.R., Middag L.D.C., Brandsma C.A.A., Gosens R. (2016). Noncanonical WNT-5B signaling induces inflammatory responses in human lung fibroblasts. Am J Physiol Lung Cell Mol Physiol.

[213] La Mensa A. Impact of cigarette smoke on inflammasome-dependent responses in human lung fibroblasts. https://iris.unipa.it/retrieve/.

[214] Dupin I., Allard B., Ozier A. (2016). Blood fibrocytes are recruited during acute exacerbations of chronic obstructive pulmonary disease through a CXCR4-dependent pathway. J Allergy Clin Immunol.

[215] Kaddah S., Selim S., Rashed L., Noaman M. (2014). Circulating fibrocytes are an indicator of severity and exacerbation in chronic obstructive pulmonary disease. Egypt J Chest Dis Tuberc.

[216] Yang J., Li Y., Huang Y., Chen H., Sui P. (2024). Unlocking lung regeneration: insights into progenitor cell dynamics and metabolic control. Cell Regen.

[217] Zacharias W.J., Frank D.B., Zepp J.A. (2018). Regeneration of the lung alveolus by an evolutionarily conserved epithelial progenitor. Nature.

[218] Ghosh M., Miller Y.E., Nakachi I. (2018). Exhaustion of airway basal progenitor cells in early and established chronic obstructive pulmonary disease. Am J Respir Crit Care Med.

[219] Vallath S., Hynds R.E., Succony L., Janes S.M., Giangreco A. (2014). Targeting EGFR signalling in chronic lung disease: therapeutic challenges and opportunities. Eur Respir J.

[220] Holgate S.T. (2011). The sentinel role of the airway epithelium in asthma pathogenesis. Immunol Rev.

[221] Woodruff P.G. (2011). Novel outcomes and end points: biomarkers in chronic obstructive pulmonary disease clinical trials. Proc Am Thorac Soc.

[222] Ganesan S., Unger B.L., Comstock A.T. (2013). Aberrantly activated EGFR contributes to enhanced IL-8 expression in COPD airways epithelial cells via regulation of nuclear FoxO3A. Thorax.

[223] Deshmukh H.S., Shaver C., Case L.M. (2008). Acrolein-activated matrix metalloproteinase 9 contributes to persistent mucin production. Am J Respir Cell Mol Biol.

[224] Gilowska I., Kasper Ł., Bogacz K. (2018). Impact of matrix metalloproteinase 9 on COPD development in polish patients: genetic polymorphism, protein level, and their relationship with lung function. BioMed Res Int.

[225] Wang Y., Shen Y., Li K. (2009). Role of matrix metalloproteinase-9 in lipopolysaccharide-induced mucin production in human airway epithelial cells. Arch Biochem Biophys.

[226] Papakonstantinou E., Karakiulakis G., Batzios S. (2015). Acute exacerbations of COPD are associated with significant activation of matrix metalloproteinase 9 irrespectively of airway obstruction, emphysema and infection. Respir Res.

[227] Parambath S., Selvraj N.R., Venugopal P., Aradhya R. (2024). Notch signaling: an emerging paradigm in the pathogenesis of reproductive disorders and diverse pathological conditions. Int J Mol Sci.

[228] Tsao P.N., Matsuoka C., Wei S.C. (2016). Epithelial Notch signaling regulates lung alveolar morphogenesis and airway epithelial integrity. Proc Natl Acad Sci U S A.

[229] Whitsett J.A., Kalinichenko V.V. (2011). Notch and basal cells take center stage during airway epithelial regeneration. Cell Stem Cell.

[230] Di Stefano A., Gnemmi I., Rosani U. (2024). Upregulation of Notch signaling and cell-differentiation inhibitory transcription factors in stable chronic obstructive pulmonary disease patients. Int J Mol Sci.

[231] Boucherat O., Chakir J., Jeannotte L. (2012). The loss of Hoxa5 function promotes Notch-dependent goblet cell metaplasia in lung airways. Biol Open.

[232] Tao Y., Sun Y., Wu B. (2021). Overexpression of FOXA2 attenuates cigarette smoke-induced cellular senescence and lung inflammation through inhibition of the p38 and ERK1/2 MAPK pathways. Int Immunopharmacol.

[233] Song J., Heijink I.H., Kistemaker L.E.M. (2017). Aberrant DNA methylation and expression of SPDEF and FOXA2 in airway epithelium of patients with COPD. Clin Epigenet.

[234] Chen G., Korfhagen T.R., Xu Y. (2009). SPDEF is required for mouse pulmonary goblet cell differentiation and regulates a network of genes associated with mucus production. J Clin Invest.

[235] Dongre A., Weinberg R.A. (2019). New insights into the mechanisms of epithelial-mesenchymal transition and implications for cancer. Nat Rev Mol Cell Biol.

[236] Sohal S.S., Walters E.H. (2013). Epithelial mesenchymal transition (EMT) in small airways of COPD patients. Thorax.

[237] Sohal S.S., Reid D., Soltani A. (2011). Evaluation of epithelial mesenchymal transition in patients with chronic obstructive pulmonary disease. Respir Res.

[238] Kalluri R., Weinberg R.A. (2009). The basics of epithelial-mesenchymal transition. J Clin Invest.

[239] Su X., Wu W., Zhu Z., Lin X., Zeng Y. (2022). The effects of epithelial-mesenchymal transitions in COPD induced by cigarette smoke: an update. Respir Res.

[240] Nowrin K., Sohal S.S., Peterson G., Patel R., Walters E.H. (2014). Epithelial-mesenchymal transition as a fundamental underlying pathogenic process in COPD airways: fibrosis, remodeling and cancer. Expert Rev Respir Med.

[241] He S., Sun S., Lu J. (2021). The effects of the miR-21/SMAD7/TGF-*β* pathway on Th17 cell differentiation in COPD. Sci Rep.

[242] Liu X., Sun S., He S., Xie L. (2025). Smad7 ameliorate small airway remodeling in COPD by modulating epithelial-mesenchymal transition. Tob Induc Dis.

[243] Gao J., Zhan B. (2012). The effects of Ang-1, IL-8 and TGF-*β*1 on the pathogenesis of COPD. Mol Med Rep.

[244] Yamada M., Fujino N., Ichinose M. (2016). Inflammatory responses in the initiation of lung repair and regeneration: their role in stimulating lung resident stem cells. Inflam Regen.

[245] Wu X., Bos I.S.T., Conlon T.M. (2022). A transcriptomics-guided drug target discovery strategy identifies receptor ligands for lung regeneration. Sci Adv.

[246] Wang H., Lv C., Wang S., Ying H., Weng Y., Yu W. (2018). NLRP3 inflammasome involves in the acute exacerbation of patients with chronic obstructive pulmonary disease. Inflammation.

[247] Yang W., Ni H., Wang H., Gu H. (2015). NLRP3 inflammasome is essential for the development of chronic obstructive pulmonary disease. Int J Clin Exp Pathol.

[248] Nachmias N., Langier S., Brzezinski R.Y. (2019). NLRP3 inflammasome activity is upregulated in an in-vitro model of COPD exacerbation. PLoS One.

[249] Markelić I., Hlapčić I., Čeri A. (2022). Activation of NLRP3 inflammasome in stable chronic obstructive pulmonary disease. Sci Rep.

[250] Sayan M., Mossman B.T. (2016). The NLRP3 inflammasome in pathogenic particle and fibre-associated lung inflammation and diseases. Part Fibre Toxicol.

[251] Zhou H., Zhang Q., Huang W. (2023). NLRP3 inflammasome mediates silica-induced lung epithelial injury and aberrant regeneration in lung stem/progenitor cell-derived organotypic models. Int J Biol Sci.

[252] Zhou H., Zhang Q., Liu C. (2024). NLRP3 inflammasome mediates abnormal epithelial regeneration and distal lung remodeling in silica-induced lung fibrosis. Int J Mol Med.

[253] Wiegman C.H., Michaeloudes C., Haji G. (2015). Oxidative stress-induced mitochondrial dysfunction drives inflammation and airway smooth muscle remodeling in patients with chronic obstructive pulmonary disease. J Allergy Clin Immunol.

[254] Genova M.L., Lenaz G. (2015). The interplay between respiratory supercomplexes and ROS in aging. Antioxid Redox Signal.

[255] Barnes P.J. (2020). Oxidative stress-based therapeutics in COPD. Redox Biol.

[256] Miwa S., Kashyap S., Chini E., von Zglinicki T. (2022). Mitochondrial dysfunction in cell senescence and aging. J Clin Invest.

[257] Araya J., Kuwano K. (2022). Cellular senescence-an aging hallmark in chronic obstructive pulmonary disease pathogenesis. Respir Investig.

[258] Amado C.A., Martín-Audera P., Agüero J. (2023). Alterations in circulating mitochondrial signals at hospital admission for COPD exacerbation. Chron Respir Dis.

[259] Aoshiba K., Nagai A. (2009). Senescence hypothesis for the pathogenetic mechanism of chronic obstructive pulmonary disease. Proc Am Thorac Soc.

[260] van Eeden S.F., Sin D.D. (2013). Oxidative stress in chronic obstructive pulmonary disease: a lung and systemic process. Can Respir J.

[261] Sapey E., Bafadhel M., Bolton C.E., Wilkinson T., Hurst J.R., Quint J.K. (2019). Building toolkits for COPD exacerbations: lessons from the past and present. Thorax.

[262] Henke M.O., John G., Rheineck C., Chillappagari S., Naehrlich L., Rubin B.K. (2011). Serine proteases degrade airway mucins in cystic fibrosis. Infect Immun.

[263] Mallia-Milanes B., Dufour A., Philp C. (2018). TAILS proteomics reveals dynamic changes in airway proteolysis controlling protease activity and innate immunity during COPD exacerbations. Am J Physiol Lung Cell Mol Physiol.

[264] Rossi A., Belmonte B., Carnevale S. (2022). Stromal and immune cell dynamics in tumor associated tertiary lymphoid structures and anti-tumor immune responses. Front Cell Dev Biol.

[265] Faner R., Cruz T., Casserras T. (2016). Network analysis of lung transcriptomics reveals a distinct B-cell signature in emphysema. Am J Respir Crit Care Med.

[266] Sullivan J.L., Bagevalu B., Glass C. (2019). B cell-adaptive immune profile in emphysema-predominant chronic obstructive pulmonary disease. Am J Respir Crit Care Med.

[267] Bracke K.R., Verhamme F.M., Seys L.J.M. (2013). Role of CXCL13 in cigarette smoke-induced lymphoid follicle formation and chronic obstructive pulmonary disease. Am J Respir Crit Care Med.

[268] Jia J., Conlon T.M., Sarker R.S. (2018). Cholesterol metabolism promotes B-cell positioning during immune pathogenesis of chronic obstructive pulmonary disease. EMBO Mol Med.

[269] van der Strate B.W.A., Postma D.S., Brandsma C.A. (2006). Cigarette smoke-induced emphysema: a role for the B cell?. Am J Respir Crit Care Med.

[270] Freeman C.M., Martinez C.H., Todt J.C. (2015). Acute exacerbations of chronic obstructive pulmonary disease are associated with decreased CD4+ & CD8+ T cells and increased growth & differentiation factor-15 (GDF-15) in peripheral blood. Respir Res.

[271] Conlon T.M., John-Schuster G., Heide D. (2020). Inhibition of LTβR signalling activates WNT-induced regeneration in lung. Nature.

[272] Tejwani V., Villabona-Rueda A.F., Khare P. (2023). Airway and systemic prostaglandin E2 association with COPD symptoms and macrophage phenotype. Chronic Obstr Pulm Dis.

[273] Chen Y., Chen P., Hanaoka M., Droma Y., Kubo K. (2008). Enhanced levels of prostaglandin E2 and matrix metalloproteinase-2 correlate with the severity of airflow limitation in stable COPD. Respirology.

[274] Savla U., Appel H.J., Sporn P.H., Waters C.M. (2001). Prostaglandin E(2) regulates wound closure in airway epithelium. Am J Physiol Lung Cell Mol Physiol.

[275] Li Y.J., Kanaji N., Wang X.Q. (2015). Prostaglandin E2 switches from a stimulator to an inhibitor of cell migration after epithelial-to-mesenchymal transition. Prostaglandins Other Lipid Mediat.

[276] Koarai A., Yamada M., Ichikawa T., Fujino N., Sugiura H. (2024). Treatment with systemic corticosteroid versus placebo for exacerbations of COPD: a systematic review and meta-analysis. Respir Investig.

[277] Walters J.A.E., Tan D.J., White C.J., Gibson P.G., Wood-Baker R., Walters E.H. (2014). Systemic corticosteroids for acute exacerbations of chronic obstructive pulmonary disease. Cochrane Database Syst Rev.

[278] Aaron S.D. (2014). Management and prevention of exacerbations of COPD. BMJ.

[279] Rosenwasser Y., Berger I., Loewy Z.G. (2022). Therapeutic approaches for chronic obstructive pulmonary disease (COPD) exacerbations. Pathogens.

[280] Ram F.S., Rodriguez-Roisin R., Granados-Navarrete A., Garcia-Aymerich J., Barnes N.C. (2006). Antibiotics for exacerbations of chronic obstructive pulmonary disease. Cochrane Database Syst Rev.

[281] Menzel M., Akbarshahi H., Bjermer L., Uller L. (2016). Azithromycin induces anti-viral effects in cultured bronchial epithelial cells from COPD patients. Sci Rep.

[282] Cuevas E., Huertas D., Montón C. (2023). Systemic and functional effects of continuous azithromycin treatment in patients with severe chronic obstructive pulmonary disease and frequent exacerbations. Front Med.

[283] Ram F.S., Picot J., Lightowler J., Wedzicha J.A. (2004). Non-invasive positive pressure ventilation for treatment of respiratory failure due to exacerbations of chronic obstructive pulmonary disease. Cochrane Database Syst Rev.

[284] Walters J.A., Tang J.N.Q., Poole P., Wood-Baker R. (2017). Pneumococcal vaccines for preventing pneumonia in chronic obstructive pulmonary disease. Cochrane Database Syst Rev.

[285] Kim S.H., Lee H., Kim M.J. (2025). Effects of vaccination on acute exacerbation of chronic obstructive pulmonary disease: a nationwide population-based cohort study. Tuberc Respir Dis.

[286] Bao W., Li Y., Wang T. (2021). Effects of influenza vaccination on clinical outcomes of chronic obstructive pulmonary disease: a systematic review and meta-analysis. Ageing Res Rev.

[287] Clark Z., Maurer D.M. (2018). Pneumococcal vaccines in chronic obstructive pulmonary disease. Am Fam Physician.

[288] Vogelmeier C.F., Criner G.J., Martinez F.J. (2017). Global strategy for the diagnosis, management, and prevention of chronic obstructive lung disease 2017 report. GOLD executive summary. Am J Respir Crit Care Med.

[289] Kumar P.A., Hu Y., Yamamoto Y. (2011). Distal airway stem cells yield alveoli in vitro and during lung regeneration following H1N1 influenza infection. Cell.

[290] Vaughan A.E., Brumwell A.N., Xi Y. (2015). Lineage-negative progenitors mobilize to regenerate lung epithelium after major injury. Nature.

[291] Ray S., Chiba N., Yao C. (2016). Rare SOX2+ airway progenitor cells generate KRT5+ cells that repopulate damaged alveolar parenchyma following influenza virus infection. Stem Cell Rep.

[292] Sun N., Ogulur I., Mitamura Y. (2024). The epithelial barrier theory and its associated diseases. Allergy.

[293] Burgel P.-R., Nesme-Meyer P., Chanez P. (2009). Cough and sputum production are associated with frequent exacerbations and hospitalizations in COPD subjects. Chest.

[294] Hansel T.T., Barnes P.J. (2009). New drugs for exacerbations of chronic obstructive pulmonary disease. Lancet.

[295] Gan H., Wang G., Hao Q., Wang Q.J., Tang H. (2013). Protein kinase D promotes airway epithelial barrier dysfunction and permeability through down-regulation of claudin-1. J Biol Chem.

[296] Rezaee F., DeSando S.A., Ivanov A.I. (2013). Sustained protein kinase D activation mediates respiratory syncytial virus-induced airway barrier disruption. J Virol.

[297] Rezaee F., Meednu N., Emo J.A. (2011). Polyinosinic:polycytidylic acid induces protein kinase D-dependent disassembly of apical junctions and barrier dysfunction in airway epithelial cells. J Allergy Clin Immunol.

[298] Guedán A., Swieboda D., Charles M. (2017). Investigation of the role of protein kinase D in human rhinovirus replication. J Virol.

[299] Zhang Q., Yan L., Lu Y. (2024). HDAC6-selective inhibitor CAY10603 ameliorates cigarette smoke-induced small airway remodeling by regulating epithelial barrier dysfunction and reversing. Respir Res.

[300] Li N., Liu B., Xiong R., Li G., Wang B., Geng Q. (2023). HDAC3 deficiency protects against acute lung injury by maintaining epithelial barrier integrity through preserving mitochondrial quality control. Redox Biol.

[301] Bertuccio F.R., Baio N., Montini S. (2024). Potential new inflammatory markers in bronchiectasis: a literature review. Curr Issues Mol Biol.

[302] Shah B.K., Singh B., Wang Y., Xie S., Wang C. (2023). Mucus hypersecretion in chronic obstructive pulmonary disease and its treatment. Mediators Inflamm.

[303] Rogliani P., Matera M.G., Page C., Puxeddu E., Cazzola M., Calzetta L. (2019). Efficacy and safety profile of mucolytic/antioxidant agents in chronic obstructive pulmonary disease: a comparative analysis across erdosteine, carbocysteine, and N-acetylcysteine. Respir Res.

[304] Mata M., Ruíz A., Cerdá M. (2003). Oral N-acetylcysteine reduces bleomycin-induced lung damage and mucin Muc5ac expression in rats. Eur Respir J.

[305] Xu K., Ma J., Lu R. (2023). Effective-compound combination of Bufei Yishen formula III combined with ER suppress airway mucus hypersecretion in COPD rats: via EGFR/MAPK signaling. Biosci Rep.

[306] Ping F., Cao Q., Lin H., Han S.Z. (2019). Antagonistic effects of N-acetylcysteine on mitogen-activated protein kinase pathway activation, oxidative stress and inflammatory responses in rats with PM2.5 induced lung injuries. Chin Med Sci J.

[307] Chi L., Shan Y., Cui Z. (2022). N-acetyl-L-cysteine protects airway epithelial cells during respiratory syncytial virus infection against mucin synthesis, oxidative stress, and inflammatory response and inhibits HSPA6 expression. Anal Cell Pathol.

[308] Mata M., Sarrion I., Armengot M. (2012). Respiratory syncytial virus inhibits ciliagenesis in differentiated normal human bronchial epithelial cells: effectiveness of N-acetylcysteine. PLoS One.

[309] Decramer M., Rutten-van Mölken M., Dekhuijzen P.N.R. (2005). Effects of N-acetylcysteine on outcomes in chronic obstructive pulmonary disease (Bronchitis Randomized on NAC Cost-Utility Study, BRONCUS): a randomised placebo-controlled trial. Lancet.

[310] Zheng J.P., Wen F.Q., Bai C.X. (2014). Twice daily N-acetylcysteine 600 mg for exacerbations of chronic obstructive pulmonary disease (Pantheon): a randomised, double-blind placebo-controlled trial. Lancet Respir Med.

[311] Kwok W.C., Chan S.K.S., Chiang K.Y., Ho C.M.J. (2024). A double-blind randomized controlled trial of N-acetylcysteine (NAC) for the treatment of acute exacerbation of chronic obstructive pulmonary disease. Respirol Case Rep.

[312] Yu Y., Miao T.W., Xiao W. (2024). Andrographolide attenuates NLRP3 inflammasome activation and airway inflammation in exacerbation of chronic obstructive pulmonary disease. Drug Des Devel Ther.

[313] Klughammer B., Piali L., Nica A. (2023). A randomized, double-blind phase 1b study evaluating the safety, tolerability, pharmacokinetics and pharmacodynamics of the NLRP3 inhibitor selnoflast in patients with moderate to severe active ulcerative colitis. Clin Transl Med.

[314] Watanabe T., Jono H., Han J., Lim D.J., Li J.D. (2004). Synergistic activation of NF-κB by nontypeable *Haemophilus influenzae* and tumor necrosis factor *α*. Proc Natl Acad Sci U S A.

[315] Gallelli L., Pelaia G., Fratto D. (2010). Effects of budesonide on P38 MAPK activation, apoptosis and IL-8 secretion, induced by TNF-α and *Haemophilus influenzae* in human bronchial epithelial cells. Int J Immunopathol Pharmacol.

[316] Chung K.F. (2005). Inflammatory mediators in chronic obstructive pulmonary disease. Curr Drug Targets Inflamm Allergy.

[317] Dominguez C., Powers D.A., Tamayo N. (2005). p38 MAP kinase inhibitors: many are made, but few are chosen. Curr Opin Drug Discov Devel.

[318] Gaffey K., Reynolds S., Plumb J., Kaur M., Singh D. (2013). Increased phosphorylated p38 mitogen-activated protein kinase in COPD lungs. Eur Respir J.

[319] Medicherla S., Fitzgerald M.F., Spicer D. (2008). p38α-selective mitogen-activated protein kinase inhibitor SD-282 reduces inflammation in a subchronic model of tobacco smoke-induced airway inflammation. J Pharmacol Exp Ther.

[320] Ahmadi A., Ahrari S., Salimian J. (2023). p38 MAPK signaling in chronic obstructive pulmonary disease pathogenesis and inhibitor therapeutics. Cell Commun Signal.

[321] Chung K.F. (2011). p38 mitogen-activated protein kinase pathways in asthma and COPD. Chest.

[322] De Buck S., Hueber W., Vitaliti A. (2015). Population PK-PD model for tolerance evaluation to the p38 MAP kinase inhibitor BCT197. CPT Pharmacometrics Syst Pharmacol.

[323] Sverzellati N., Lynch D., Pistolesi M. (2014). Physiologic and quantitative computed tomography differences between centrilobular and panlobular emphysema in COPD. J COPD F.

[324] Strâmbu I.R., Kobalava Z.D., Magnusson B.P., MacKinnon A., Parkin J.M. (2019). Phase II study of single/repeated doses of Acumapimod (BCT197) to treat acute exacerbations of COPD. Copd.

[325] Pascoe S., Costa M., Marks-Konczalik J., McKie E., Yang S., Scherbovsky P.S. (2017). Biological effects of p38 MAPK inhibitor losmapimod does not translate to clinical benefits in COPD. Respir Med.

[326] Patel N.R., Cunoosamy D.M., Fagerås M. (2018). The development of AZD7624 for prevention of exacerbations in COPD: a randomized controlled trial. Int J Chronic Obstruct Pulm Dis.

[327] Martucci C., Allen A.D., Moretto N. (2024). CHF6297: a novel potent and selective p38 MAPK inhibitor with robust anti-inflammatory activity and suitable for inhaled pulmonary administration as dry powder. Front Pharmacol.

[328] Stolfa I., Page C. (2023). Phosphodiesterase inhibitors and lung diseases. Adv Pharmacol.

[329] Matera M.G., Rogliani P., Calzetta L., Cazzola M. (2014). Phosphodiesterase inhibitors for chronic obstructive pulmonary disease: what does the future hold?. Drugs.

[330] Cazzola M., Calzetta L., Rogliani P., Matera M.G. (2024). The need for inhaled phosphodiesterase inhibitors in chronic obstructive pulmonary disease. Expert Rev Clin Pharmacol.

[331] Abbott-Banner K.H., Page C.P. (2014). Dual PDE3/4 and PDE4 inhibitors: novel treatments for COPD and other inflammatory airway diseases. Basic Clin Pharmacol Toxicol.

[332] Franciosi L.G., Diamant Z., Banner K.H. (2013). Efficacy and safety of RPL554, a dual PDE3 and PDE4 inhibitor, in healthy volunteers and in patients with asthma or chronic obstructive pulmonary disease: findings from four clinical trials. Lancet Respir Med.

[333] Martin C., Burgel P.R., Roche N. (2021). Inhaled dual phosphodiesterase 3/4 inhibitors for the treatment of patients with COPD: a short review. Int J Chron Obstruct Pulmon Dis.

[334] Calzetta L., Rogliani P. (2024). Ensifentrine approval: a milestone in the treatment of COPD. Pulm Pharmacol Ther.

[335] Donohue J.F., Rheault T., MacDonald-Berko M., Bengtsson T., Rickard K. (2023). Ensifentrine as a novel, inhaled treatment for patients with COPD. Int J Chron Obstruct Pulmon Dis.

[336] Anzueto A., Barjaktarevic I.Z., Siler T.M. (2023). Ensifentrine, a Novel phosphodiesterase 3 and 4 Inhibitor for the Treatment of chronic obstructive pulmonary disease: randomized, Double-Blind, Placebo-controlled, Multicenter Phase III Trials (the ENHANCE Trials). Am J Respir Crit Care Med.

[337] Keam S.J. (2024). Ensifentrine: first approval. Drugs.

[338] Giembycz M.A., Field S.K. (2010). Roflumilast: first phosphodiesterase 4 inhibitor approved for treatment of COPD. Drug Des Devel Ther.

[339] Varricchi G., Pecoraro A., Marone G. (2018). Thymic stromal lymphopoietin isoforms, inflammatory disorders, and cancer. Front Immunol.

[340] Ying S., O’Connor B., Ratoff J. (2008). Expression and cellular provenance of thymic stromal lymphopoietin and chemokines in patients with severe asthma and chronic obstructive pulmonary disease. J Immunol.

[341] Singh D., Brightling C.E., Rabe K.F. (2025). Efficacy and safety of tezepelumab versus placebo in adults with moderate to very severe chronic obstructive pulmonary disease (COURSE): a randomised, placebo-controlled, phase 2a trial. Lancet Respir Med.

[342] England E., Rees D.G., Scott I.C. (2023). Tozorakimab (MEDI3506): an anti-IL-33 antibody that inhibits IL-33 signalling via ST2 and RAGE/EGFR to reduce inflammation and epithelial dysfunction. Sci Rep.

[343] Reid F., Singh D., Albayaty M. (2024). A randomized Phase I study of the anti-interleukin-33 antibody tozorakimab in healthy adults and patients with chronic obstructive pulmonary disease. Clin Pharmacol Ther.

[344] Pandya H., Guller P., Reid F. (2024). Late Breaking Abstract-FRONTIER-4: a phase 2a study to investigate tozorakimab (anti-IL-33 mAb) in COPD. Eur Respir Soc.

[345] Cazzola M., Hanania N.A., Page C.P., Matera M.G. (2023). Novel anti-inflammatory approaches to COPD. Int J Chron Obstruct Pulmon Dis.

[346] Riera-Martínez L., Cànaves-Gómez L., Iglesias A., Martin-Medina A., Cosío B.G. (2023). The role of IL-33/ST2 in COPD and its future as an antibody therapy. Int J Mol Sci.

[347] Rabe K.F., Celli B.R., Wechsler M.E. (2021). Safety and efficacy of itepekimab in patients with moderate-to-severe COPD: a genetic association study and randomised, double-blind, phase 2a trial. Lancet Respir Med.

[348] Rabe K.F., Martinez F.J., Bhatt S.P. (2024). AERIFY-1/2: two phase 3, randomised, controlled trials of itepekimab in former smokers with moderate-to-severe COPD. ERJ Open Res.

[349] Kaltwasser J. Itepekimab shows mixed results in phase 3 COPD trials. https://www.ajmc.com/view/itepekimab-shows-mixed-results-in-phase-3-copd-trials.

[350] Crescioli S., Kaplon H., Wang L., Visweswaraiah J., Kapoor V., Reichert J.M. (2025). Antibodies to watch in 2025. MAbs.

[351] Butler J.P., Loring S.H., Patz S., Tsuda A., Yablonskiy D.A., Mentzer S.J. (2012). Evidence for adult lung growth in humans. N Engl J Med.

[352] Hogan B.L.M., Barkauskas C.E., Chapman H.A. (2014). Repair and regeneration of the respiratory system: complexity, plasticity, and mechanisms of lung stem cell function. Cell Stem Cell.

[353] Rock J., Königshoff M. (2012). Endogenous lung regeneration: potential and limitations. Am J Respir Crit Care Med.

[354] Gindele J.A., Kiechle T., Benediktus K. (2020). Intermittent exposure to whole cigarette smoke alters the differentiation of primary small airway epithelial cells in the air-liquid interface culture. Sci Rep.

[355] Di Vincenzo S., Ninaber D.K., Cipollina C., Ferraro M., Hiemstra P.S., Pace E. (2022). Cigarette smoke impairs airway epithelial wound repair: role of modulation of epithelial-mesenchymal transition processes and Notch-1 signaling. Antioxidants.

[356] Barnes P.J., Anderson G.P., Fagerås M., Belvisi M.G. (2021). Chronic lung diseases: prospects for regeneration and repair. Eur Respir Rev.

[357] Ng-Blichfeldt J.P., Gosens R., Dean C., Griffiths M., Hind M. (2019). Regenerative pharmacology for COPD: breathing new life into old lungs. Thorax.

[358] McCluskey E.S., Liu N., Pandey A., Marchetti N., Kelsen S.G., Sajjan U.S. (2024). Quercetin improves epithelial regeneration from airway basal cells of COPD patients. Respir Res.

[359] Yan J., Zhang W., Feng Y. (2024). Autologous transplantation of P63+ lung progenitor cells in patients with bronchiectasis: a randomized, single-blind, controlled trial. Cell Rep Med.

[360] Kim Y.S., Kim J.Y., Cho R., Shin D.M., Lee S.W., Oh Y.M. (2017). Adipose stem cell-derived nanovesicles inhibit emphysema primarily via an FGF2-dependent pathway. Exp Mol Med.

[361] D’Agostino B., Sullo N., Siniscalco D., De Angelis A., Rossi F. (2010). Mesenchymal stem cell therapy for the treatment of chronic obstructive pulmonary disease. Expert Opin Biol Ther.

[362] Wang G., Bunnell B.A., Painter R.G. (2005). Adult stem cells from bone marrow stroma differentiate into airway epithelial cells: potential therapy for cystic fibrosis. Proc Natl Acad Sci U S A.

[363] Spees J.L., Olson S.D., Ylostalo J. (2003). Differentiation, cell fusion, and nuclear fusion during ex vivo repair of epithelium by human adult stem cells from bone marrow stroma. Proc Natl Acad Sci U S A.

[364] Mendez J.J., Ghaedi M., Steinbacher D., Niklason L.E. (2014). Epithelial cell differentiation of human mesenchymal stromal cells in decellularized lung scaffolds. Tissue Eng Part A.

[365] Sui B.D., Zheng C.X., Li M., Jin Y., Hu C.H. (2020). Epigenetic regulation of mesenchymal stem cell homeostasis. Trends Cell Biol.

[366] Jin Z., Pan X., Zhou K. (2015). Biological effects and mechanisms of action of mesenchymal stem cell therapy in chronic obstructive pulmonary disease. J Int Med Res.

[367] Kim Y.S., Kim J.Y., Huh J.W., Lee S.W., Choi S.J., Oh Y.M. (2015). The therapeutic effects of optimal dose of mesenchymal stem cells in a murine model of an elastase induced-emphysema. Tuberc Respir Dis.

[368] Chen Y., Zhang F., Wang D. (2020). Mesenchymal stem cells attenuate diabetic lung fibrosis via adjusting Sirt3-mediated stress responses in rats. Oxid Med Cell Longev.

[369] Tzouvelekis A., Laurent G., Bouros D. (2013). Stem cell therapy in chronic obstructive pulmonary disease. Seeking the Prometheus effect. Curr Drug Targets.

[370] Le Visage C., Dunham B., Flint P., Leong K.W. (2004). Coculture of mesenchymal stem cells and respiratory epithelial cells to engineer a human composite respiratory mucosa. Tissue Eng.

[371] Weiss D.J., Casaburi R., Flannery R., LeRoux-Williams M., Tashkin D.P. (2013). A placebo-controlled, randomized trial of mesenchymal stem cells in COPD. Chest.

[372] Stolk J., Broekman W., Mauad T. (2016). A phase I study for intravenous autologous mesenchymal stromal cell administration to patients with severe emphysema. Qjm.

[373] Armitage J., Tan D.B.A., Troedson R. (2018). Mesenchymal stromal cell infusion modulates systemic immunological responses in stable COPD patients: a phase I pilot study. Eur Respir J.

[374] Eggenhofer E., Benseler V., Kroemer A. (2012). Mesenchymal stem cells are short-lived and do not migrate beyond the lungs after intravenous infusion. Front Immunol.

[375] de Oliveira H.G., Cruz F.F., Antunes M.A. (2016). Combined bone marrow-derived mesenchymal stromal cell therapy and one-way endobronchial valve placement in patients with pulmonary emphysema: a phase I clinical trial. Stem Cells Transl Med.

[376] Watanabe H., Tsuchiya T., Shimoyama K. (2018). Adipose-derived mesenchymal stem cells attenuate rejection in a rat lung transplantation model. J Surg Res.

[377] Zuk P.A., Zhu M., Mizuno H. (2001). Multilineage cells from human adipose tissue: implications for cell-based therapies. Tissue Eng.

[378] Kern S., Eichler H., Stoeve J., Klüter H., Bieback K. (2006). Comparative analysis of mesenchymal stem cells from bone marrow, umbilical cord blood, or adipose tissue. Stem Cells.

[379] Melief S.M., Zwaginga J.J., Fibbe W.E., Roelofs H. (2013). Adipose tissue-derived multipotent stromal cells have a higher immunomodulatory capacity than their bone marrow-derived counterparts. Stem Cells Transl Med.

[380] Schmelzer E., Miceli V., Chinnici C.M., Bertani A., Gerlach J.C. (2020). Effects of mesenchymal stem cell coculture on human lung small airway epithelial cells. BioMed Res Int.

[381] Nguyen T.T., Phan P.T., Nguyen B.H. (2021). Autologous adipose-derived stem cells therapy in COPD treatment: a case report. Respirol Case Rep.

[382] Monterroso Yancor B., Basegoda Curiel W.S., Lemus-Martin R. (2024). Adipose-derived stem cells therapy for moderate chronic obstructive pulmonary disease in adult subjects: a phase III randomized, multicenter trial: RESPIRE protocol. Princ Pract Clin Res.

[383] Kanno Y., Mitsui T., Sano H., Kitta T., Moriya K., Nonomura K. (2014). Contribution of bone marrow-derived mesenchymal stem cells to the morphological changes in the bladder after partial outlet obstruction: a preliminary study. Int J Urol.

[384] Taléns-Visconti R., Bonora A., Jover R. (2006). Hepatogenic differentiation of human mesenchymal stem cells from adipose tissue in comparison with bone marrow mesenchymal stem cells. World J Gastroenterol.

[385] Le Thi Bich P., Nguyen Thi H., Dang Ngo Chau H. (2020). Allogeneic umbilical cord-derived mesenchymal stem cell transplantation for treating chronic obstructive pulmonary disease: a pilot clinical study. Stem Cell Res Ther.

[386] Hoang D.M., Nguyen K.T., Nguyen A.H., Nguyen B.N., Nguyen L.T. (2021). Allogeneic human umbilical cord-derived mesenchymal stem/stromal cells for chronic obstructive pulmonary disease (COPD): study protocol for a matched case-control, phase I/II trial. BMJ Open.

[387] Karaoz E., Kalemci S., Ece F. (2020). Improving effects of mesenchymal stem cells on symptoms of chronic obstructive pulmonary disease. Bratisl Lek Listy.

[388] Vu S.-P., Veit K., Sadikot R.T. (2025). Molecular approaches to treating chronic obstructive pulmonary disease: current perspectives and future directions. Int J Mol Sci.

[389] Shi M.M., Yang Q.Y., Monsel A. (2021). Preclinical efficacy and clinical safety of clinical-grade nebulized allogenic adipose mesenchymal stromal cells-derived extracellular vesicles. J Extracell Vesicles.

[390] Nikfarjam S., Rezaie J., Zolbanin N.M., Jafari R. (2020). Mesenchymal stem cell derived-exosomes: a modern approach in translational medicine. J Transl Med.

[391] Ridzuan N., Zakaria N., Widera D. (2021). Human umbilical cord mesenchymal stem cell-derived extracellular vesicles ameliorate airway inflammation in a rat model of chronic obstructive pulmonary disease (COPD). Stem Cell Res Ther.

[392] Zhu Y.G., Feng X.M., Abbott J. (2014). Human mesenchymal stem cell microvesicles for treatment of Escherichia coli endotoxin-induced acute lung injury in mice. Stem Cells.

[393] Monsel A., Zhu Y.G., Gennai S. (2015). Therapeutic effects of human mesenchymal stem cell-derived microvesicles in severe pneumonia in mice. Am J Respir Crit Care Med.

[394] Song Q., Zhou A., Cheng W. (2025). Bone marrow mesenchymal stem cells-derived exosomes inhibit apoptosis of pulmonary microvascular endothelial cells in COPD mice through miR-30b/Wnt5a pathway. Int J Nanomedicine.

[395] Harrell C.R., Miloradovic D., Sadikot R. (2020). Molecular and cellular mechanisms responsible for beneficial effects of mesenchymal stem cell-derived product “exo-d-MAPPS” in attenuation of chronic airway inflammation. Anal Cell Pathol.

[396] Pan X., Wang L., Yang J. (2024). TRβ activation confers AT2-to-AT1 cell differentiation and anti-fibrosis during lung repair via KLF2 and CEBPA. Nat Commun.

[397] Liang J., Zhang Y., Xie T. (2016). Hyaluronan and TLR4 promote surfactant-protein-C-positive alveolar progenitor cell renewal and prevent severe pulmonary fibrosis in mice. Nat Med.

[398] Nabhan A.N., Webster J.D., Adams J.J. (2023). Targeted alveolar regeneration with Frizzled-specific agonists. Cell.

[399] Shao S., Zhang N., Specht G.P. (2024). Pharmacological expansion of type 2 alveolar epithelial cells promotes regenerative lower airway repair. Proc Natl Acad Sci U S A.

[400] Zepp J.A., Zacharias W.J., Frank D.B. (2017). Distinct mesenchymal lineages and niches promote epithelial self-renewal and Myofibrogenesis in the lung. Cell.

[401] Paris A.J., Hayer K.E., Oved J.H. (2020). STAT3-BDNF-TrkB signalling promotes alveolar epithelial regeneration after lung injury. Nat Cell Biol.

[402] Li J., Masood A., Yi M. (2013). The IGF-I/IGF-R1 pathway regulates postnatal lung growth and is a nonspecific regulator of alveologenesis in the neonatal rat. Am J Physiol Lung Cell Mol Physiol.

[403] Treutlein B., Brownfield D.G., Wu A.R. (2014). Reconstructing lineage hierarchies of the distal lung epithelium using single-cell RNA-seq. Nature.

[404] Basil M.C., Cardenas-Diaz F.L., Kathiriya J.J. (2022). Human distal airways contain a multipotent secretory cell that can regenerate alveoli. Nature.

[405] Kadur Lakshminarasimha Murthy P., Sontake V., Tata A. (2022). Human distal lung maps and lineage hierarchies reveal a bipotent progenitor. Nature.

[406] Uhlén M., Fagerberg L., Hallström B.M. (2015). Proteomics. Tissue-based map of the human proteome. Science.

[407] Yen F.-S., Wei J.C.-C., Ko F.-S. (2025). Association of DPP-4 inhibitors with respiratory and cardiovascular complications in patients with COPD: a nationwide cohort study. ERJ Open Res.

[408] Zhang S., Liu Y., Zhang X. (2018). Prostaglandin E2 hydrogel improves cutaneous wound healing via M2 macrophages polarization. Theranostics.

[409] Cao X., Duan L., Hou H. (2020). IGF-1C hydrogel improves the therapeutic effects of MSCs on colitis in mice through PGE2-mediated M2 macrophage polarization. Theranostics.

[410] Dackor R.T., Cheng J., Voltz J.W. (2011). Prostaglandin E_2_ protects murine lungs from bleomycin-induced pulmonary fibrosis and lung dysfunction. Am J Physiol Lung Cell Mol Physiol.

[411] Birrell M.A., Maher S.A., Dekkak B. (2015). Anti-inflammatory effects of PGE2 in the lung: role of the EP4 receptor subtype. Thorax.

[412] Buckley J., Birrell M.A., Maher S.A., Nials A.T., Clarke D.L., Belvisi M.G. (December 2011). EP4 receptor as a new target for bronchodilator therapy. Thorax.

[413] Dagouassat M., Gagliolo J.M., Chrusciel S. (2013). The cyclooxygenase-2-prostaglandin E2 pathway maintains senescence of chronic obstructive pulmonary disease fibroblasts. Am J Respir Crit Care Med.

[414] Hezam K., Wang C., Fu E. (2023). Superior protective effects of PGE2 priming mesenchymal stem cells against LPS-induced acute lung injury (ALI) through macrophage immunomodulation. Stem Cell Res Ther.

